# Pharmacological Management of Cancer Pain: Advances in Treatment Strategies and Drug Delivery Systems

**DOI:** 10.3390/pharmaceutics18010006

**Published:** 2025-12-20

**Authors:** Xueying Yang, Rong Zhang, Aijia Wang, Dan Zhang, Jiangxue Cheng, Bingtao Zhai, Dongyan Guo

**Affiliations:** 1 School of Pharmacy, Shaanxi University of Chinese Medicine, Xi’an 712046, China; 18429079077@163.com (X.Y.); 18031945864@163.com (R.Z.); 15091611352@163.com (A.W.); 18392739884@163.com (D.Z.); cjx511@sntcm.edu.cn (J.C.); 2 Shaanxi Province Key Laboratory of New Drugs and Chinese Medicine Foundation Research, Shaanxi University of Chinese Medicine, Xi’an 712046, China

**Keywords:** cancer pain, drug mechanisms, integrated traditional Chinese and Western medicine treatment, delivery systems

## Abstract

Cancer pain seriously damages the quality of life of patients, and its management urgently needs new strategies with both efficacy and safety. This review deeply analyzes the clinical limitations of WHO’s third-order analgesic strategy in cancer pain management, especially emphasizes the unique value of integrated traditional Chinese and Western medicine in synergy and reduction in adverse reactions, and summarizes the network interaction of related drugs through the regulation of multi-target analgesic mechanisms such as inflammatory factors, ion channels, neurotransmitters, and even glial cells and osteoclast activity in the tumor microenvironment. Building on this foundation, the article systematically analyzed the clinical advantages and limitations of drug delivery systems (DDS): oral sustained and controlled drug delivery system, mucosal drug delivery system (MDDS), transdermal drug delivery system (TDDS), and intrathecal targeted drug delivery (ITDD) in the treatment of cancer pain for the first time. The development prospects of new DDS: microneedles, disposable intrathecal drug delivery, and nano-drug delivery system (NDDS) in cancer pain were summarized in detail. Looking ahead, research into the analgesic mechanisms of drugs holds promise for providing a theoretical foundation for cancer pain management. Collaborative strategies integrating Chinese and Western medicine, coupled with precision delivery technologies, are expected to advance more efficient and safer pain control, offering new approaches and methods for achieving superior pain management outcomes.

## 1. Introduction

Cancer pain is one of the most common and distressing symptoms experienced by cancer patients, presenting a significant clinical challenge in management. Statistics indicate that 60% of patients undergoing anticancer treatment will experience pain, with 50% suffering moderate to severe pain and 30% enduring excruciating, unbearable pain [1]. Furthermore, 33% to 40% of survivors who completed curative treatment continue to experience chronic pain [2]. The etiology of cancer pain is complex and multifactorial, encompassing pain directly caused by the tumor, pain resulting from tumor infiltration and vascular obstruction, pain arising from tumor obstruction of hollow or solid organ lumens, and pain triggered by mucosal ulceration and infection [3]. Pain may also occur during diagnostic procedures such as gastroscopy, colonoscopy, or esophagoscopy, when instruments damage peripheral receptors. Additionally, pain associated with cancer treatments-including postoperative pain, chemotherapy-induced pain, drug tolerance, and post-radiotherapy pain-presents significant challenges. Pain unrelated to the cancer itself or its diagnosis/treatment, as well as psychologically induced pain, also holds considerable importance [4]. Consequently, cancer pain remains a major clinical challenge. Its high prevalence, complexity, and profound impact on patients’ quality of life make effective management particularly difficult [5].

The World Health Organization (WHO) three-step ladder approach to cancer pain management has established the foundation for standardized global cancer pain care. For patients with mild cancer pain, non-opioid medications are prioritized, while weak opioids are initiated for moderate cancer pain, escalating to strong opioids as pain worsens [6]. For neuropathic cancer pain, adjunctive medications such as antiepileptics, tricyclic antidepressants (TCAs), and N-methyl-D-aspartate (NMDA) antagonists are required [7]. However, these approaches may be accompanied by numerous adverse reactions [8] and ofen demonstrate poor efficacy in advanced stages. In recent years, traditional Chinese medicine (TCM) has demonstrated unique advantages in treating cancer pain [9]. Formulas such as Peony and Licorice Decoction, Huachansu Capsules, Xihuang Pills, and Compound Kushen Injection can be combined with Western analgesics for cancer pain management. These combinations reduce adverse reactions while enhancing pain relief [10], offering novel approaches and combination strategies for cancer pain management. Ultimately, the efficacy of these interventions depends on precise targeting of the complex mechanisms underlying cancer pain.

Research indicates that the pathophysiological mechanisms of cancer pain involve multiple parallel and interacting pathways, including the release of inflammatory mediators, abnormal activation of ion channels, bone destruction, central sensitization, and glial cell activation [11]. Accordingly, existing drugs exert their effects through various mechanisms, such as inhibiting inflammatory factors, regulating ion channels, suppressing osteoclast activity, or modulating central sensitization. For example, nonsteroidal anti-inflammatory drugs (NSAIDs) [12], corticosteroids [13], and many traditional Chinese medicines suppress inflammatory factors [14]; anticonvulsants, local anesthetics, and Peony and Licorice Decoction modulate ion channels; bisphosphonates [15] and Huachansu inhibit osteoclast activation; while NMDA receptor antagonists like ketamine and TCAs target central sensitization [16]. Additionally, drugs such as Huachansu suppress glial cell activation [17]. These findings provide valuable insights for developing more synergistic treatment strategies.

Beyond this, drug delivery systems (DDS) have demonstrated significant potential in managing cancer pain. Oral controlled-release DDS enable prolonged, controlled analgesic effects while reducing dosing frequency [18]. Mucosal DDS provide rapid pain relief, making them particularly suitable for breakthrough cancer pain (BTP) [19]. Transdermal DDS maintain sustained drug release, offering long-lasting and consistent efficacy [20]. Intrathecal DDS minimize systemic side effects and provide a rapid onset of action [21]. Furthermore, the emergence of novel DDS—including implantable devices, targeted small molecules, nanoparticles (NPs), microspheres, and liposomes—has opened new avenues for cancer pain management.

Given the complex pathophysiological mechanisms underlying cancer pain, existing three-tier pain management guidelines fail to meet clinical demands. Current research predominantly focuses on the pathogenesis of cancer pain, the role of individual targets in cancer pain, and the clinical application of analgesics within the three-tier framework. However, no systematic review has yet been conducted on the development of integrated Chinese and Western medicine approaches or DDS in cancer pain management. Therefore, this paper searched databases including the Cochrane Library, PubMed, SCI, CNKI, and X-MOL using keywords such as “cancer pain,” “cancer pain treatment drugs,” “combination of Chinese and Western medicine,” “cancer pain mechanisms,” and “DDS.” It reviews advances in the three-step analgesic approach for cancer pain treatment, identifies the unique advantages of integrated Chinese and Western medicine therapy, and summarizes the mechanisms of action of the aforementioned drugs in cancer pain management. Furthermore, the potential of DDS in cancer pain management is emphasized, and the application prospects of novel DDS in cancer pain are discussed. This aims to provide new strategies and insights with clear guidance for future research and clinical practice in cancer pain treatment.

## 2. Drug Management of Cancer Pain

### 2.1. WHO Three-Step Pain Management

In 1986, the WHO published the Pain Management Guidelines for Cancer and recommended the global implementation of this protocol. Clinical practice has demonstrated that standardized application of the three-step approach can effectively control 80% to 90% of cancer-related pain, significantly improving patients’ quality of life [22]. See Table 1 for details.

The core of pharmacological treatment involves escalating medication based on pain intensity. The first step employs non-opioid medications for mild cancer pain, including acetaminophen (APAP) and NSAIDs. Since APAP alone has limited efficacy in relieving cancer pain [23], it is often combined with weak opioid medications. NSAIDs are the preferred treatment for mild pain. Commonly used NSAIDs include ibuprofen, diclofenac, celecoxib, etoricoxib, and loxoprofen. They are particularly effective in the presence of inflammation but carry long-term side effects such as gastrointestinal ulcers, gastrointestinal bleeding, platelet dysfunction, liver and kidney damage, and cardiac toxicity [24]. When NSAIDs or APAP reach their dosage limits or their adverse effects become intolerable, switching to other types of analgesics is recommended. Considering the potential risks of long-term use, cancer pain may be managed with either monotherapy or combination therapy with opioids based on individual circumstances [25].

The second step involves the use of weak opioids, such as tramadol and buprenorphine, for managing moderate pain; however, repeated or prolonged use may cause nausea, dizziness, respiratory depression, and mild dependence [26,27]. The third step prioritizes potent opioids, including morphine, oxycodone, fentanyl, hydromorphone, and methadone, which can be administered orally, intravenously, or subcutaneously for dose titration [28]. In palliative care, a direct transition from non-opioid analgesics to opioids is increasingly preferred. Additionally, opioid tolerance developed during long-term use reduces analgesic efficacy, necessitating dosage escalation by clinicians. This escalation can lead to addiction and physical dependence, representing a significant limitation to their sustained clinical use [29]. Moreover, prolonged opioid use may cause adverse reactions such as constipation, intractable vomiting, and respiratory depression. These side effects not only diminish patients’ quality of life but also compromise treatment efficacy. Therefore, developing strategies to reduce opioid dosages while maintaining effective pain management is crucial in clinical practice.

Additionally, adjunctive medications are often combined for patients with neuropathic cancer pain, including antiepileptic drugs (gabapentin, pregabalin), TCAs, local anesthetics (lidocaine, ropivacaine), bisphosphonates, corticosteroids (dexamethasone, prednisone), alpha-2 adrenergic agonists (clonidine, dexmedetomidine, tizanidine), and NMDA receptor antagonists (ketamine). These agents provide multimodal analgesia while reducing opioid dependence and adverse effects [30].

**Table 1:** WHO third-order pain medications.

Classification of Drugs	Marketed Drugs	Initial Time to Market	Clinical Use for Cancer pain	Common Medication Dosage Forms and Dosages	Short-Term Side Effects	Long-Term Side Effects
APAP	Paracetamol tablets [31]	1955 (US)	Mild pain	Tablets, Capsules, Suppositories, Suspensions; 650 mg/4 h or 1 g/6 h; ≤4 g/d	Overdose may cause acute liver toxicity	Hepatotoxic
NSAIDs	Ibuprofen [32]	1969 (UK)	Mild to moderate cancer pain with inflammation	Tablets, Granules; ≤3200 mg/d	GI irritation, nausea, abdominal pain	GI irritation; liver, kidney damage
	Diclofenac sodium tablets [33]	1988 (US)	Bone metastasis pain, Postoperative mild to moderate pain, Cancerous arthritis pain	Tablets, Suppositories, Topical Emulsions, Transdermal Patches; ≤150 mg/qd	GI discomfort, headache; local skin reactions (topical use)	GI tract; kidneys, cardiovascular risks
	Aspirin enteric-coated tablets [34]	1889 (Germany)	Mild pain accompanied by fever and bone metastases	Tablets, Suppositories; 30–60 mg/tid	GI irritation, tinnitus, bleeding tendency	GI bleeding; Reye’s syndrome risk (children/adolescents); Salicylate poisoning
	Naproxen tablets [35]	1976 (US)	Chronic and acute pain	Tablets, Suspensions; ≤1500 mg/d	GI discomfort, dizziness, drowsiness	Risk of cardiovascular thrombosis; GI ulcers, and bleeding
	Celecoxib [36]	1998 (US)	Osteoarthritis/RA Pain, Visceral Pain	Capsules; ≤200 mg/bid	Cardiovascular and GI risks	Cardiovascular thrombosis; renal impairment
	Meloxicam tablets [37]	1996 (European)	Bone metastasis pain, Chronic cancer pain from soft tissue tumors	Tablets, Suppositories; 7.5 mg/qd	Indigestion, nausea, abdominal pain	Risk of nephrotoxicity and bone marrow suppression
	Indomethacin tablets [38]	1965 (US)	Moderate cancer pain	Tablets, Suppositories; 25 mg/bid	Significant GI reactions, headache, dizziness	GI ulcers, central nervous system toxicity, bone marrow suppression
	Ketorolac injection [39]	1990 (Italy)	Moderate to severe acute cancer pain, Preferred for short-term control	Injections, Tablets; 15–30 mg/6 h, ≤5 d	High risk of GI bleeding; injection site pain; renal impairment	Long-term use can easily lead to severe gastrointestinal bleeding and acute renal failure.
Weak opioids	Tramadol [40]	1977 (Germany)	Moderate to severe acute and chronic pain	Tablets, Capsules, Injections; ≤400 mg/d	Nausea, vomiting, dizziness, drowsiness	Tolerance and Dependence
	Buprenorphine injection [41]	1981 (US)	Chronic Cancer Pain	Injections, Oral Dispersible Tablets, Transdermal patch; ≤900 μg/d	Nausea, drowsiness, constipation; risk of respiratory depression	Physical dependence; withdrawal symptoms occurring after discontinuation
Strong opioids	Morphine tablets [42]	1827 (Germany)	Moderate to severe cancer pain, Breakthrough Pain, Postoperative Pain	Tablets 15–60 mg/d, Injections 15–40 mg/d	Nausea, vomiting, constipation, drowsiness, respiratory depression	Tolerance, physical and psychological dependence
	Oxycodone extended-release tablets [43]	1995 (US)	Moderate to severe cancer pain, Neuropathic pain	Tablets; ≤40 mg/d	Constipation, nausea, dizziness	Tolerance, Dependence
	Fentanyl injections [44]	1968 (US)	Persistent cancer pain, Breakthrough pain	Transdermal Patches 12–100 mg/q 72 h, Injections 0.05–0.1 mg/dose, Lozenges, Nasal Sprays; -	Dizziness, blurred vision, nausea, vomiting, hypotension	Respiratory depression, asphyxia, muscle rigidity, and tachycardia
	Hydromorphone hydrochloride injection [45]	1972 (US)	Moderate to severe cancer pain	Injection, Tablet, Capsule; ≥8 mg/d	Dizziness, nausea, sweating	Respiratory depression and apnea
	Methadone tablets [46]	1947 (Germany)	Refractory Cancer Pain	Tablets, Injections; ≤45 mg/d	Risk of QT interval prolongation, complex drug interactions, accumulation and toxicity risks	Long half-life, respiratory depression, complex long-term management, high potential for addiction
Anticonvulsants	Gabapentin capsules [47]	1993 (US)	Neuropathic Cancer Pain	Capsule, Tablet, Oral Liquid; 900–3600 mg/d	Dizziness, drowsiness, fatigue, ataxia	Weight gain, peripheral edema
	Pregabalin capsules [48]	2004 (US)	Neuropathic Cancer Pain	Capsules, Oral Liquid; ≤600 mg/d	Dizziness, drowsiness, blurred vision	Weight gain, peripheral edema
tricyclic antidepressants	Duloxetine capsules [49]	2004 (US)	Neuropathic cancer pain with depression	Enteric-coated capsules; ≤120 mg/d	Nausea, dry mouth, insomnia, constipation; may increase suicide risk in the early stages	Liver and kidney damage, discontinuation syndrome
	Amitriptyline tablets [50]	1961 (US)	Neuropathic cancer pain	Tablets; 50–150 mg/qn	Dry mouth, constipation, blurred vision, drowsiness	Cardiac toxicity, weight gain, cognitive effects
Local anesthetic	Lidocaine injection [51]	1948 (US)	Localized cancer pain	Injections, Patches, Gels; ≤200 mg/dose	Local skin reactions, systemic toxicity	Skin allergy or intolerance, systemic toxicity
	Ropivacaine injection [52]	1996 (US)	Acute pain	Injections; 20–40 mg/d	Hypotension, nausea, vomiting, bradycardia, abnormal sensations	Risk of localized tissue toxicity or nerve damage
Bisphosphonates	Zoledronic acid powder injection [53]	2001 (US)	Bone Metastasis Cancer Pain	Injections; 4 mg, IV ≥15 min, 3–4 dose/weeks	Fever, bone pain, hypocalcemia, nephrotoxicity	Mandibular necrosis, atypical femoral fracture
The glucocorticoid hormone	Dexamethasone tablets [54]	1958 (US)	Acute nerve compression pain, intracranial hypertension pain	Tablets 0.75–3 mg/bid, Injections IV 2–20 mg/dose	Mood swings, elevated blood sugar, insomnia, indigestion	Cushing’s syndrome, osteoporosis, immunosuppression, muscle atrophy, cataracts
	Prednisone tablets [55]	1955 (US)	Chronic cancer pain with inflammation	Tablets 10–60 mg/d	Fluid retention, hypertension, and hyperglycemia are more pronounced	Adrenal cortex suppression
α-2 adrenergic agonists	Clonidine tablets [56]	1974 (US)	Adjuvant Therapy for Refractory Cancer Pain	Tablets 0.1–0.3 mg/bid, Transdermal Patches 0.1–0.3 mg/d	Dry mouth, drowsiness, dizziness, hypotension, bradycardia	Long-term use leads to tolerance
	Dexmedetomidine injection [57]	1999 (US)	Sedation and Analgesia for Cancer Pain in the Intensive Care Unit	Injections 0.2–0.7 μg/kg/h	Hypotension, bradycardia	Tolerance
	Tizanidine tablets [58]	1996 (US)	Spasmodic cancer pain	Tablets 2–4 mg/tid, ≤36 mg/d	Drowsiness, dry mouth, dizziness, low blood pressure, risk of liver toxicity	Hepatotoxicity and nephrotoxicity, tolerability
NMDA receptor antagonist	Ketamine injection [59]	1970 (US)	Refractory cancer pain	Injections, Nasal Sprays;	Psychiatric symptoms, hypertension, tachycardia, nausea	Neurotoxicity, cognitive impairment

APAP: Acetaminophen; bid: Bis in die (twice a day); d: Day; GI: Gastrointestinal; h: Hour; IV: Intravenous; mg: Milligram; NMDA: N-Methyl-D-Aspartate; NSAIDs: Non-Steroidal Anti-Inflammatory Drugs; qd: Quaque die (once a day); q: Every (e.g., q 72 h); RA: Rheumatoid Arthritis; tid: Ter in die (three times a day).

### 2.2. Clinical Application of Combined Chinese and Western Medicine in the Treatment of Cancer Pain

In standardized cancer pain management, while Western pharmaceutical analgesic regimens based on the WHO three-step ladder have significantly improved patient outcomes. However, patients with moderate to severe cancer pain often encounter challenges such as opioid dose dependence and adverse reactions. Therefore, it is crucial to identify proactive and effective cancer pain treatment strategies that minimize the side effects associated with Western medications. In recent years, the emergence of integrated Chinese and Western medicine therapies have emerged as effective options for relieving cancer pain. These approaches provide comprehensive benefits, including analgesia, anti-inflammation, antitumor activity, and immune modulation. Within comprehensive cancer pain management, they demonstrate long-term efficacy by enhancing therapeutic outcomes while reducing toxicity. Specific details are provided in Table 2.

Among these, the Kidney-Nourishing Bone-Strengthening Formula and Kidney-Nourishing Pain-Relieving Granules are derived from Liuwei Dihuang Wan, with modifications incorporating herbs that resolve stasis and detoxify. These formulas have the effects of nourishing the kidneys and replenishing marrow, strengthening tendons and bones, and resolving stasis and detoxifying. Clinically, they are frequently used to treat somatic cancer pain and bone metastasis-related cancer pain [84]. Song Hongli [60,61] found that combining the Yishen Gukang Formula with hydrocodone bitartrate extended-release tablets demonstrated superior efficacy in alleviating pain compared to Western medicine alone in patients with moderate-to-severe cancer-related somatic pain presenting with kidney deficiency and blood stasis syndrome. Furthermore, the Yishen Gukang Formula significantly reduced the frequency of breakthrough pain episodes and the required dosage of OxyContin, thereby mitigating adverse reactions such as dizziness, constipation, and nausea associated with high-dose opioid use. Zhang Qinglin [62] found that combining Yishen Qutong Granules with hydrocodone bitartrate extended-release tablets significantly reduced Numerical Rating Scale (NRS) scores, decreased the frequency of breakthrough pain episodes, and lowered OxyContin dosage in patients with moderate-to-severe kidney deficiency with toxin-stasis syndrome and bone metastasis cancer pain. The treatment demonstrated good safety.

Xuefu Zhuyu Tang, derived from Wang Qingren’s Medical Forest Corrections, is effective in promoting blood circulation, removing blood stasis, regulating qi, and alleviating pain. Xu Zhengyin [66] found that combining Xuefu Zhuyu Tang with hydrocodone bitartrate extended-release tablets significantly reduced NRS scores, cancer pain frequency, and adverse reactions (nausea, vomiting, constipation, anorexia) compared to the control group in patients with cancer pain caused by qi deficiency and blood stasis syndrome. Zheng Qiao [65] reported that combining Blood Residence Stasis-Resolving Decoction with standardized pain management reduced opioid consumption and improved clinical efficacy in patients with advanced lung cancer experiencing cancer pain caused by blood stasis obstructing the collaterals. Zhang Hongsheng [64] treated patients with liver cancer pain due to blood stasis obstruction syndrome using a combination of Blood Residence Stasis-Resolving Decoction and morphine hydrochloride sustained-release tablets. The treatment group demonstrated significantly higher overall pain relief rates and lower NRS scores compared to the control group. Levels of prostaglandin E2 (PGE2) and nitric oxide (NO) were significantly reduced, confirming the definite efficacy of this formula in treating cancer pain.

Yuanhu Zhitong Tablets are a commonly used oral analgesics in clinical practice, containing two Chinese herbal medicines with analgesic properties: Angelica dahurica and Corydalis yanhusuo. Although their analgesic potency is weaker than that of opioids, they offer convenient administration, fewer adverse reactions, and reliable safety [85]. Tan Dan [63] combined Yuanhu Zhitong Tablets with strong opioid medications to manage moderate-to-severe postoperative pain in lung cancer patients. The study demonstrated significant analgesic efficacy with fewer adverse reactions, enhancing patient tolerance and improving treatment compliance.

The bone pain patch has warming yang properties, dispels cold, unblocks meridians, and relieves pain. It is clinically used to treat cancer pain caused by bone metastases. Hou Zhaolin [67] combined the bone pain patch with oxycodone hydrochloride extended-release tablets to treat bone metastasis cancer pain characterized by a yin-cold stagnation pattern. This combination significantly reduced NRS scores, demonstrated superior efficacy compared to oxycodone hydrochloride alone, and showed no significant skin toxicity reactions, indicating a high level of safety.

The primary active ingredient in Aconitine Capsules is aconitine, which exhibits potent analgesic effects. Li Fang [83] demonstrated that combining Aconitine Capsules with morphine sulfate sustained-release tablets in patients with moderate-to-severe cancer pain reduced the required dosage of morphine sulfate sustained-release tablets and decreased the incidence of adverse reactions.

Yanghe Decoction, derived from the Complete Collection of Surgical Diagnosis and Treatment, has the effects of yang to stop bleeding and dispelling cold to unblock stagnation. It is commonly used clinically to treat lung cancer bone metastases. Zhang Chunmei [69] found that Yanghe Decoction synergistically enhances the efficacy of zoledronic acid, thereby more effectively alleviating pain caused by bone metastases. Feng Lei [68] demonstrated that combining Yanghe Decoction with zoledronic acid effectively reduces pain from bone metastases, improves clinical outcomes, enhances patients’ quality of life, and lowers TCM syndrome scores, while maintaining good safety. These findings suggest that Yanghe Decoction can serve as an adjunctive therapy for patients with bone metastases from breast cancer exhibiting the yang deficiency and cold stagnation pattern.

The Peony and Licorice Decoction, first documented in the Treatise on Cold Damage Diseases, plays a positive role in harmonizing qi and blood, soothing the liver, and relieving pain. Feng Xiaofei [70] demonstrated that combining Peony and Licorice Decoction with extended-release hydrocodone bitartrate tablets in patients with moderate-to-severe cancer pain improved pain relief rates, reduced pain intensity and breakthrough pain episodes, decreased adverse reactions, and enhanced patient prognosis. This combination may serve as a standard regimen for managing moderate-to-severe cancer pain. In particular, among patients with ovarian cancer pain, this combination therapy reduced the average daily hydrocodone dose by 24.69 mg, accelerated the average onset time by 13.26 min, prolonged analgesic duration by 2.11 h, and significantly lowered levels of pain-related factors, including serotonin (5-HT), PGE2, substance P, tumor necrosis factor-alpha (TNF-α), interleukin-1β (IL-1β), and IL-6 [71].

Similarly, Xihuang Pills—a pure herbal anticancer medication derived from the ancient Xihuang formula—possess analgesic, antipyretic, detoxifying, blood-activating, stasis-resolving, and swelling-reducing properties. They are widely used to treat various mid-to-late-stage malignant tumors. Hong You [72] combined Xihuang Pills with extended-release hydrocodone hydrochloride tablets to treat 80 patients experiencing moderate-to-severe cancer pain from lung, esophageal, liver, and rectal cancers. This combined therapy increased the overall pain relief rate by 11.67% and reduced the incidence of adverse reactions by 27.15%. Among lung cancer pain patients, the treatment efficacy rate in the observation group was 14% higher than in the control group. At 1 h, 12 h, 1 week, and 2 weeks post-administration, the NRS scores in the observation group were consistently lower than those in the control group. The incidence rates of adverse reactions such as constipation, dizziness or drowsiness, nausea or vomiting, and urinary difficulty were comparable between the two groups [73]. Thus, combining Xihuang Pills with hydrocodone bitartrate extended-release tablets enhances analgesic efficacy and improves quality of life in patients with moderate-to-severe cancer pain. Yan Qingyuan’s [73] study demonstrates that this combination therapy in lung cancer pain patients achieves synergistic, rapid pain relief without increasing adverse reactions, advancing the development of integrated Chinese and Western medicine approaches for cancer pain management.

Additionally, huachansu, the primary active component extracted from the entire skin of the Chinese toad Bufo bufo gargarizans Cantor, has been widely used in patients with mid-to-late-stage cancer pain, demonstrating particularly pronounced efficacy for bone metastasis-related pain [86]. Yangsen [77] found that combining Huachansu capsules with fentanyl transdermal patches significantly alleviated pain in patients with moderate to severe bone metastases without increasing adverse reactions. Feng Zhang [78] observed that co-administering Huachansu capsules with zoledronic acid injections improved bone density and calcium and phosphorus metabolism, reduced pain symptoms, and enhanced safety in prostate cancer patients with bone metastases. Fang Yun [74] combined Huachansu capsules with hydrocodone bitartrate extended-release tablets for cancer pain management, observing enhanced efficacy, reduced adverse reactions, and improved quality of life. Zhang Yi [75] demonstrated that Huachansu capsules combined with hydrocodone bitartrate extended-release tablets alleviated pain severity and reduced adverse reaction rates in patients with moderate-to-severe cancer pain, indicating Huachansu as an effective adjunctive Chinese herbal medicine for improving cancer pain symptoms. Dong Xueshan [76] found that combining morphine sulfate sustained-release tablets with Huachansu capsules significantly improved pain management outcomes and quality of life while reducing the average daily dosage of morphine sulfate sustained-release tablets.

Compound Kushen Injection is a pure herbal preparation primarily derived from Sophora flavescens, known for its hemostatic and analgesic properties. Xiang Pei [79] demonstrated that combining Compound Kushen Injection with standardized pain management significantly enhanced analgesic efficacy in patients with severe cancer pain while reducing opioid analgesic dosage and adverse reactions. Zhou Li [80] showed that adding Compound Kushen Injection to hydrocodone bitartrate extended-release tablets and pregabalin for neuropathic cancer pain alleviated patient discomfort. The analgesic effect improved with combined therapy, and the addition of Compound Kushen Injection further controlled pain while decreasing adverse events. Miao Jidong [81] found that combining Compound Kushen Injection with sustained-release morphine hydrochloride tablets for moderate-to-severe cancer pain provided enhanced analgesia and reduced adverse event rates. Zhang Jingjing [82] reported that co-administering Compound Kushen Injection with fentanyl transdermal patches significantly decreased gastrointestinal reactions without causing hepatic or renal damage. This combination also reduced the release of inflammatory factors, effectively alleviating cancer pain.

## 3. Therapeutic Mechanisms of Drugs

**Figure 1:**
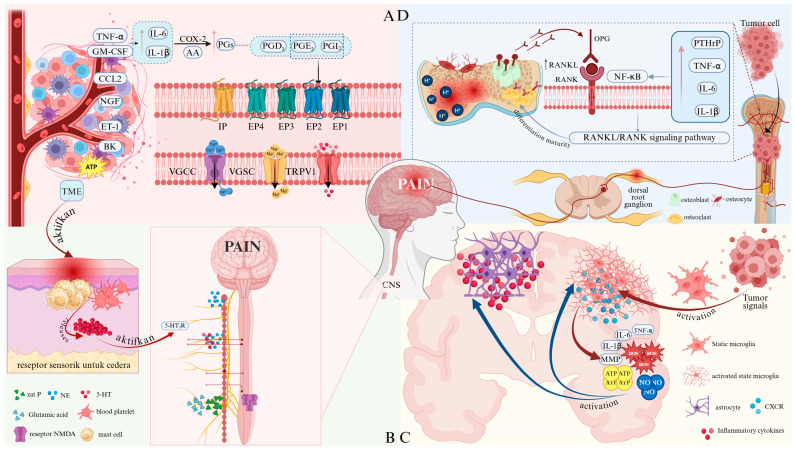
Mechanisms of Cancer Pain Development. (**A**) The complex ecosystem surrounding tumor cells collectively forms the TME, which releases inflammatory mediators such as NGF, TNF-α, GM-GSF, IL-6, IL-1β, CCL2, ATP, and ET-1. These mediators convert the COX-2 substrate AA to PGs, thereby modulating EP and IP receptors on the cell membrane and regulating TRPV1, VGSC, and VGCC ion channels. (**B**) The TME activates the release of 5-HT from mast cells and platelets in peripheral injury receptors and activates 5-HT_3_R in the CNS, which produces pain mediators such as NE, 5-HT, SP, Glu, and activates NMDA receptors. (**C**) Tumor signals activate microglia in the brain to release inflammatory factors including NO, ATP, MMP, and ROS, which in turn activate microglia and astrocytes to release inflammatory cytokines, forming a positive feedback loop. (**D**) When tumors metastasize to the bone, PTHrP, TNF-α, IL-1β, and IL-6 are released, which stimulate RANK ligand RANKL synthesis on osteoclasts, and RANKL competitively binds to OPG on osteoblasts, activating the RANKL/RANK pathway. This induces osteoclast differentiation and maturation, leading to bone destruction and the release of large amounts of H^+^ ions, acidifying the bone microenvironment. Pain signals are transmitted from the DRG to the brain, resulting in pain perception. “Created in BioRender. Ying, L. (2025) https://BioRender.com/d04hkrx”. 5-HT: 5-Hydroxytryptamine; 5-HT_3_R: 5-Hydroxytryptamine receptor 3; AA: Arachidonic Acid; ATP: Adenosine Triphosphate; CCL2: C-C Motif Chemokine Ligand 2; CNS: Central Nervous System; COX-2: Cyclooxygenase-2; DRG: Dorsal Root Ganglia; EP: Prostaglandin E receptor; ET-1: Endothelin-1; Glu: Glutamate; IL-1β: Interleukin-1 beta; IL-6: Interleukin-6; IP: Prostaglandin I2 receptor; MMP: Matrix Metalloproteinases; NE: Norepinephrine; NGF: Nerve Growth Factor; NMDA: N-Methyl-D-Aspartate; NO: Nitric Oxide; OPG: Osteoprotegerin; PGs: Prostaglandins; PTHrP: Parathyroid Hormone-related Protein; RANK: Receptor Activator of Nuclear Factor Kappa-B; RANKL: RANK Ligand; ROS: Reactive Oxygen Species; SP: Substance P; GM-GSF: Granulocyte-macrophage colony-stimulating factor; TME: Tumor Microenvironment; TNF-α: Tumor Necrosis Factor alpha; TRPV1: Transient Receptor Potential Vanilloid 1; VGCC: Voltage-Gated Calcium Channels; VGSC: Voltage-Gated Sodium Channels.

Although the three-step analgesic ladder and integrated Chinese and Western medicine therapies have established well-defined application protocols in the clinical management of cancer pain, their efficacy fundamentally depends on intervening in the complex pathological mechanisms underlying this condition. Cancer pain, a multifaceted symptom associated with tumors, involves multiple pathways, including the release of inflammatory factors, regulation of ion channels, activation of osteoclasts, and release of pain mediators (Figure 1). Current pharmacological treatments achieve therapeutic effects by targeting these key pathways. For specific details, see Table 3.

### 3.1. Regulation Inflammatory Factors in the Tumor Microenvironment

#### 3.1.1. Direct Inhibition of Inflammatory Factors

The origin of the cancer pain loop lies within the tumor tissue itself and its surrounding microenvironment (TME). Tumor cells, immune cells (such as macrophages and T cells), and tumor-associated fibroblasts continuously release large quantities of pro-pain mediators, including protons (H^+^), inflammatory cytokines, adenosine triphosphate (ATP), endothelin-1 (ET-1), and others. These substances persistently act on peripheral nociceptors, lowering their activation threshold and enhancing their responsiveness. This process leads to peripheral sensitization and the generation of cancer pain [112,113,114].

IL-6, a key member of the interleukin family, is secreted by macrophages, fibroblasts, T lymphocytes, and other cells, playing a crucial role in various inflammatory responses and diseases [115]. Evidence indicates that IL-6 enhances Toll-like receptor (TLR)-mediated cytokine production, suggesting its involvement in exacerbating inflammatory responses associated with cancer pain. IL-6 can also be self-induced in certain tumor cells, promoting their growth [116]. The release of IL-1β activates multiple signaling pathways, thereby enhancing pain perception. Furthermore, IL-1β upregulates macrophage expression of cyclooxygenase-2 (COX-2), amplifying prostaglandin (PG) synthesis [117] and inducing severe pain. Granulocyte-macrophage colony-stimulating factor (GM-CSF) contributes to increased IL-1β secretion [118]. TNF-α stimulates the release of IL-1β and IL-6, activates nociceptors, and exerts pro-nociceptive effects [119]. C-C chemokine ligand 2 (CCL2) promotes immune cell infiltration into the tumor microenvironment (TME), exacerbating inflammation and cancer pain perception [120]. The P2X3 receptor (purinergic receptor P2X, ligand-gated ion channel, 3) is highly and selectively expressed in small- and medium-diameter sensory neurons, mediating pain signal transmission [121]. Nerve growth factor (NGF) activates its high-affinity homologous receptor TrkA (tyrosine kinase receptor A), triggering intracellular signaling cascades that modulate the TME innervated by sensory and sympathetic nerve fibers, thereby inducing cancer pain [122]. ET-1 directly activates nociceptors and potentiates the pain-inducing effects of other noxious substances, including capsaicin, formaldehyde, and arachidonic acid (AA). Additionally, endothelial cells produce ATP, which sensitizes ET-1 receptors; released ATP activates P2X2/3 receptors on nociceptors, inducing pain generation [123]. Bradykinin (BK) increases nociceptor sensitivity, promotes cancer pain progression, and interacts with other inflammatory mediators to exacerbate pain [124].

**Figure 2:**
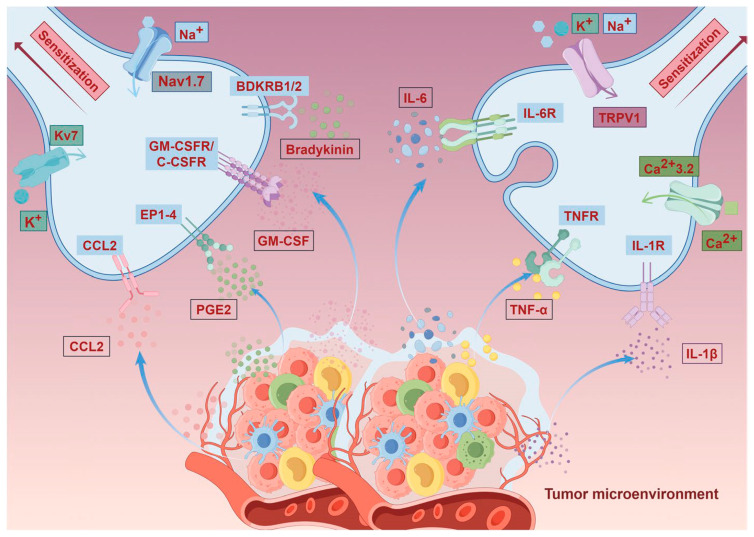
The tumor microenvironment constitutes a dynamic cellular ecosystem that secretes multiple algogenic mediators that collectively induce peripheral sensitization of tumor-innervating sensory neurons. Pro-nociceptive factors including CCL2, PGE2, GM-CSF, IL-6, TNF-α and IL-1β bind to their cognate receptors, triggering downstream phosphorylation events that lower neuronal activation thresholds. This results in enhanced signal transduction through downstream pathways. Concurrently, Nav1.7 undergoes phosphorylation-induced activation while Kv7 experiences functional downregulation. These changes synergize with TRPV1 sensitization and Ca^2+^ 3.2 activation to generate ectopic action potentials. The resultant neuronal hyperexcitability manifests clinically as spontaneous pain and mechanical hyperalgesia. Reproduced with permission from reference. *Molecular biomedicine* vol. 6,1 45. 28 June 2025 [129]. Creative Commons Attribution 4.0 International License. Copyright © 2025. The Author(s).

In summary, the interactions among these inflammatory mediators—IL-6, IL-1β, TNF-α, CCL2, PGE2, GM-CSF, and BK—highlight their synergistic role in cancer pain mechanisms (Figure 2). Targeting these mediators may open new avenues for therapeutic interventions to alleviate pain in cancer patients. For instance, NSAIDs reduce inflammatory factors released by neutrophils and macrophages, such as IL-6 and TNF-α, thereby suppressing local inflammatory responses and alleviating inflammation-induced pain [88]. Hydrocodone decreases TNF-α and IL-1β levels, relieving cancer pain [125]. Amitriptyline reduces peripheral TNF-α production, mitigating inflammatory pain [96]. Lidocaine selectively inhibits P2X7 receptor expression to exert analgesic effects [126]. The Peony and Licorice Decoction may alleviate inflammatory pain by suppressing TNF-α expression and reducing leukocyte infiltration [127]. Huachansu inhibits the release of pro-pain mediators such as TNF-α, IL-1β, IL-6, and CCL2, thereby suppressing bone cancer pain (BCP) [128]. These findings demonstrate that direct inhibition of pain mediators effectively alleviates cancer pain.

#### 3.1.2. Inhibition of COX Conversion to PGs

In anti-injury strategies, inhibiting COX reduces the conversion of its substrate, AA, into PGs. Inflammatory stimuli-induced overexpression of COX-2 leads to increased PGs in tumor regions. Among various PGs, PGE2, PGD2, and prostacyclin (PGI2) have been most extensively studied in nociception, with PGE2 typically associated with the induction of hyperalgesia. PGE2 and PGI2 activate EP and IP receptors on nociception, enhancing the activity of vanilloid transient receptor potential vanilloid 1 (TRPV1) channels and sodium channels (Nav1.9) through PKA-dependent phosphorylation, leading to mechanical and thermal hyperalgesia [88]. In contrast to PGE2 and PGI2, PGD2 modulates nociception through both peripheral and central mechanisms, either enhancing or reducing pain perception. Therefore, regulating the release of AA in the TME can effectively alleviate the onset of pain.

Among known therapeutic agents, APAP and NSAIDs reduce pain sensitization in inflammatory pain by inhibiting COX-1 and COX-2 in peripheral tissues, thereby decreasing the conversion of AA to PGs, thromboxanes, and PGI2. This occurs through reduced PGs synthesis [87]. Celecoxib, as a COX-2 inhibitor, exerts a direct targeted effect. APAP selectively inhibits COX-1 and COX-2 in the central nervous system (CNS), reducing central PGs synthesis and diminishing pain signal transmission [92]. Corticosteroids primarily suppress inflammation and edema by inhibiting phospholipase A2, reducing PGs and leukotrienes in peripheral tissues, thereby alleviating pain associated with tumor compression [93]. The boswellic acid components in Xihuangwan downregulate COX-2 expression, decreasing the conversion of AA to PGs [102], thereby exerting analgesic effects.

### 3.2. Regulation of Ion Channels

Ion channels are specialized proteins located on membranes or organelles that form highly selective pores in the phospholipid bilayer, allowing passively transported charged ions of appropriate size to pass through. Widely distributed across various tissues and organs, they participate in numerous physiological and pathological processes, serving as critical components for neuronal excitability and neural signal transmission. They have now emerged as potential therapeutic targets for treating multiple diseases, including tumors [130]. Dysfunction of voltage-gated ion channels frequently disrupts their function, leading to excessive activation of the nervous system and consequently causing persistent cancer pain.

#### 3.2.1. Regulation of TRPV1

Somatic neurons involved in pain transmission reside in dorsal root ganglia (DRG), where TRPV1 is primarily expressed in nociceptors and sensory neurons. As a non-selective cation channel belonging to the transient receptor potential (TRP) family, TRPV1 functions as a nociceptor activated by noxious heat, protons, and capsaicin [131]. According to current research, TRPV1 plays a paradoxical role in the inflammatory process. Increased inflammatory mediators induce hyperalgesia to heat and abnormal mechanical pain while affecting TRPV1 [132]. Current research indicates that TRPV1 plays a paradoxical role in the inflammatory process. Elevated inflammatory mediators induce hyperalgesia and abnormal mechanical pain while simultaneously affecting TRPV1 [132]. Direct mechanisms include binding to the receptor to activate downstream kinases that phosphorylate TRPA1 or activating TRPA1 through the production of lipid mediators [133]. Consequently, persistent inflammation within the TME serves as a key driver for the generation and maintenance of TRPA1-dependent pain signaling. Furthermore, TRPA1 activation induces neuronal depolarization and promotes the release of pain-related signaling peptides like substance P and calcitonin gene-related peptide (CGRP). These substances not only transmit pain signals to the CNS but also exert paracrine effects on surrounding cells [134]. Research indicates that modulating the function of TRPA1 channels can prevent the onset of pain symptoms. For instance, opioids inhibit TRPV1-mediated signaling pathways, promote the nuclear translocation of β-inhibin 2, and exert analgesic effects [135]. The Peony and Licorice Decoction suppresses cancer pain by downregulating TRPV1 channels and reducing the expression levels of TNF-α and IL-1β [106]. Bulleyaconitine A inhibits TRPV1-mediated SNARE-dependent exocytosis on cell surfaces and reduces TRPV1 expression in peripheral nerve axons [109].

#### 3.2.2. Regulation of Sodium Ion Channels

Pain signals are typically transmitted to the CNS via action potentials, whose rapid rise is mediated by voltage-gated sodium channels (VGSCs). Among the nine identified pore-forming α subunits (Nav1.1–1.9), the Nav1.7 and Nav1.8 subunits are key molecules involved in peripheral pain processing and increased pain sensitivity associated with inflammation and tissue injury [136]. Research indicates that, High concentrations of fluoxetine (SSRI) and venlafaxine (SNRI) in TCAs block Na^+^ channels, reducing abnormal neuronal discharge [96]. Anticonvulsants such as phenytoin, lamotrigine, carbamazepine, and oxcarbazepine also decrease ectopic nerve impulses by inhibiting Na^+^ channels. Local anesthetics block VGSCs to halt action potential conduction [99]. Lidocaine reduces the peak current of Na^+^ channels and accelerates the inactivation process, thereby decreasing neuronal excitability and preventing or alleviating pain perception [137]. Bulleyaconitine A can block VGSC-mediated ectopic discharges in neuropathic pain, exerting analgesic effects [110]. Compound Kushen injection alleviates cancer-related bone pain by reducing Nav1.7 expression in the spinal cord and DRG [107].

#### 3.2.3. Regulation of Calcium Ion Channels

Voltage-gated calcium channels (VGCCs) play a pivotal role in pain signaling within the dorsal spinal cord. Calcium influx, primarily mediated by VGCCs in neuronal membranes, is crucial for pain signal transmission, with increased calcium currents enhancing excitability [138]. Research indicates that, gabapentin and pregabalin reduce α2-δ1 VGCC activity in the spinal cord and CNS [139]. They exert anti-hyperalgesic effects by binding to the Cavα2δ subunits of L-, N-, and P/Q-type VGCCs, thereby modulating presynaptic calcium influx and subsequent calcium-mediated glutamate release, which reduces neurotransmitters involved in pain processing [98]. Huachansu exerts analgesic effects by modulating L-VGCC current properties in rat DRG cells [128].

### 3.3. Regulation of Neurotransmitters

**Figure 3:**
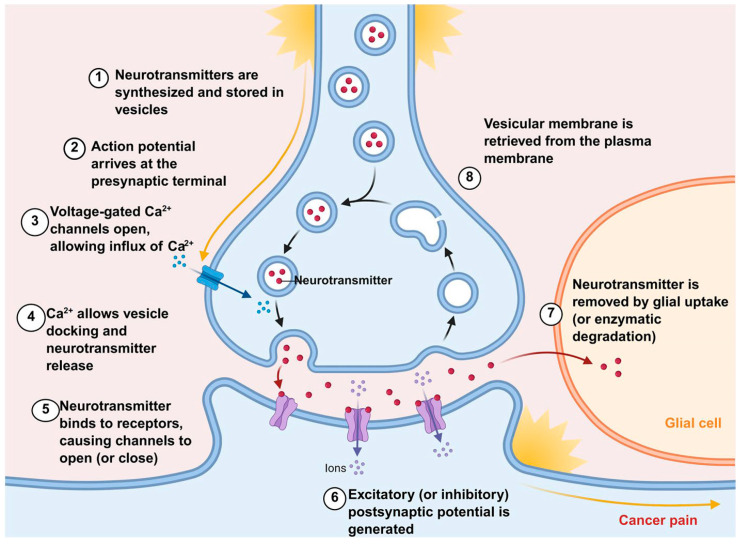
The development of central sensitization in cancer pain is largely dependent on synaptic communication within the CNS. This process, which includes synaptic plasticity and neurotransmitter release, can lead to heightened cancer pain perception when there is prolonged or excessive activation. In cancer pain, these mechanisms are often dysregulated, resulting in abnormal presynaptic glutamate release and postsynaptic receptor hypersensitivity. Consequently, sustained neuronal hyperexcitability occurs, ultimately leading to chronic cancer pain states. Reproduced with permission from reference. *Molecular biomedicine* vol. 6,1 45. 28 June 2025 [129]. Creative Commons Attribution 4.0 International License. Copyright © 2025. The Author(s).

The importance of neurotransmitters in cancer pain is a complex field of research, playing a crucial role in regulating pain pathways. Various neurotransmitters, including 5-HT, dopamine, and glutamate, are implicated in modulating pain perception and the development of cancer-related pain syndromes (Figure 3).

#### 3.3.1. Regulation of NMDA Receptors

Glutamate is the primary excitatory neurotransmitter in the CNS. Research indicates that elevated glutamate levels in the TME sensitize pain pathways, contributing to cancer-associated chronic pain conditions [140]. The NMDA receptor, a glutamate receptor, serves as the primary excitatory neurotransmitter in the human brain. Extensive research indicates that activation of NMDA receptors in the CNS plays a crucial role in the generation and maintenance of neuropathic pain, effectively alleviating pain. When tumor-related peripheral signals persistently input, synaptic efficacy in spinal dorsal horn neurons increases, sensitivity in superficial dorsal horn neurons rises, and the ratio between nociception-specific neurons and wide dynamic range (WDR) neurons shifts. This activates NMDA receptors, prolonging response time by 4–5 times [141]. It is evident that modulating NMDA receptors can alleviate pain. For example, Ketamine, as a non-competitive NMDA receptor antagonist, possesses the ability to block NMDA receptors. It reduces pain impulse transmission by decreasing central neuronal excitability, inhibits glial cell activation, and reduces neuroinflammation [94]. Opioid use alters neurotransmitter release, which helps alleviate cancer pain [129].

#### 3.3.2. Regulation of 5-HT and NE

5-HT is a monoamine widely distributed throughout the nervous system. As a neurotransmitter, it participates in pain regulation and can influence the efficacy of analgesics. Upon peripheral tissue injury or inflammation, platelets and mast cells release 5-HT, which can either alleviate or exacerbate pain [142]. 5-HT release activates 5-HT_3_R, inducing descending facilitation in the rostral ventromedial nucleus (RVM) of the medulla, thereby transmitting nociceptive stimuli and promoting central sensitization and the formation of nociceptive stimuli [143]. Upregulation of 5-HT input into RVM maintains TRPV1 sensitization [144], leading to mechanical hypersensitivity. The interaction between 5-HT and norepinephrine (NE) further complicates cancer pain. NE may be involved in the intrinsic control of pain, with its main sources being the peripheral sympathetic nerves and the central NE brainstem nuclei A1-A7. In the spinal cord, NE released from descending pathways inhibits pain by suppressing α-2A adrenergic receptors at the central terminals of primary afferent nociceptors (presynaptic inhibition), directly acting on pain-transmitting neurons via α-2 adrenergic effects (postsynaptic inhibition), and activating inhibitory interneurons mediated by α-1 adrenergic receptors [145].

Research indicates that TCAs block presynaptic reuptake of 5-HT and NE, activating descending inhibitory pathways from the brainstem to the spinal dorsal horn to suppress pain signal transmission [95]. α-2 adrenergic agonists stimulate brainstem regions, enhance downward inhibitory pathways, inhibit the transmission of injurious signals by WDR neurons in the posterior horn of the spinal cord, promote endorphin release, inhibit substance P release, activate the 5-HT pathway, and promote the release of enkephalins, which directly inhibit pain signaling [97]. Opioids inhibit pain signal transmission by activating central and peripheral μ, κ, and δ receptors, suppressing substance P and glutamate release from primary afferent neurons in the dorsal spinal horn, reducing postsynaptic neuronal excitation, activating the periaqueductal gray (PAG) and RVM, promoting 5-HT and NE release, and inhibiting ascending pain signals [90]. Ketamine interrupts cholinergic transmission and inhibits 5-HT and NE uptake, exerting an analgesic effect [15]. Aconitine tablets activate descending pain inhibitory systems via 5-HT and NE. They also stimulate the release of enkephalins from spinal dorsal horn microglia through the Gs/cAMP/PKA/p28β/CREB signaling pathway. These enkephalins act on k-opioid receptors on postsynaptic membrane neurons to produce analgesic effects [146]. It is evident that inhibiting 5-HT and NE can significantly improve cancer pain.

### 3.4. Inhibition of Glial Cell Activation

**Figure 4:**
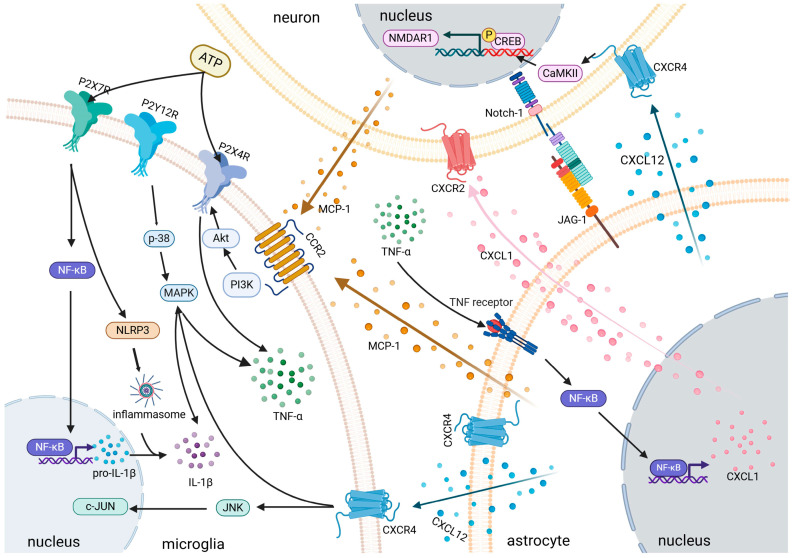
Crosstalk between the microglia, astrocytes, and neurons in the spinal cord. The microglia and astrocytes release pro-inflammatory factors or cytokines that act on the neurons, causing electrophysiological and biochemical changes within the neuron to facilitate nociceptive signaling. Neurons also communicate with the microglia and astrocytes through cytokines secretion and cell-to-cell interactions. Reproduced with permission from reference. *Journal of pain research* vol. 18 315–326. 20 January 2025 [147]. Creative Commons Attribution 3.0 International License. Copyright © 2025 Wang et al.

The TME releases a large number of molecular signals that directly activate glial cells. Activated glial cells enhance nociception by releasing inflammatory mediators, which can either directly increase neuronal excitability or structurally alter pain transmission pathways (Figure 4).

Among these, microglia serve as crucial immune and defense cells within the nervous system. When tumor-associated biomolecules cause neuronal damage or inflammation, and microglia become activated, they release large quantities of neuroactive and inflammatory mediators. These substances include ROS, IL-1β, IL-6, TNF-α, MMPs, chemokines, ATP, and NO. On one hand, these active substances act on corresponding receptors on neurons, causing pain. On the other hand, they activate more microglia and astrocytes, creating a positive feedback loop and ultimately participating in the induction and maintenance of chronic pain [148]. By upregulating surface C-X chemokine receptors (CXCRs) such as CXCR3 and CXCR4, which mediate signaling between neurons and microglia via chemokines secreted by spinal neurons, the excitability and sensitivity of sensory neurons are enhanced [149]. This leads to the continuous synthesis of nociceptive proteins, maintaining the pain perception of nociceptors and sustaining cancer pain [150]. In BCP models, tumor-mediated bone destruction generates lactic acid, PGE2, extracellular ATP, and high-mobility group box 1 (HMGB1), which stimulate purinergic receptors (purinergic receptor p2x ligand-gated ion channel 4/purinergic receptor p2x ligand-gated ion channel 7, P2X4/P2X7) and Toll-like receptor 4 (TLR4) in microglia, inducing a proinflammatory M1 phenotype [151].

Astrocytes are the most abundant cells in the CNS. Their structure is closely associated with synaptic architecture, facilitating the formation of neuronal circuits and maintaining synaptic integrity [152]. Activation of astrocytes releases various inflammatory cytokines, inducing peripheral and central sensitization, thereby contributing to cancer pain. In the BCP model, neuronal chemokines (C-X3-C motif chemokine ligand 1, CX3CL1) or IL-1β and TNF-α activate astrocytes, leading to increased glial fibrillary acidic protein (GFAP) expression and cellular hypertrophy. Additionally, astrocytes participate in crosstalk with neurons by releasing Chemokine C-X-C Ligand 1 (CXCL1) and Chemokine C-X-C Ligand 12 (CXCL12), which act on the CXCR2 and CXCR4 receptors on neurons, respectively.

Research has found that the analgesic effect of morphine is associated with a reduction in pro-inflammatory factors such as IL-1β and TNF-α secreted by M1 microglia, along with an increase in the secretion of anti-inflammatory factors like IL-10 and TGF-β, thereby promoting the transition of M1 microglia to the M2 phenotype [91]. Hydrocodone maintains long-lasting and potent analgesic effects by inhibiting the activation of astrocytes and microglia in the spinal cord [153]. Huachansu alleviates pain by reducing inflammatory responses through suppression of astrocyte and microglial activation [104]. Peony and Licorice Decoction alleviates neuropathic pain by regulating the interaction between microglia and astrocytes via the HSP90AA1/HMGB1 pathway [154]. Thus, inhibiting glial cell activation significantly alleviates pain.

### 3.5. Inhibition of Osteoclast Activation

Osteoclasts are the only cells in the body capable of bone resorption. They degrade bone matrix by secreting acidic substances and proteases, thereby maintaining bone metabolic balance. When tumors metastasize to bone, osteoclast stimulatory factors secreted by the bone microenvironment—such as parathyroid hormone-related protein (PTHrP), TNF-α, IL-1β, IL-6, etc.—stimulate the synthesis of receptor activator of NF-κB ligand (RANKL). RANKL competitively binds to osteoprotegerin (OPG), activating the RANKL/RANK pathway to induce osteoclast differentiation and maturation, leading to bone destruction [155]. Under the influence of bone tumors, osteoblasts can induce inflammatory responses, thereby reducing OPG levels and enhancing osteoclast secretion of acidic substances and bone-dissolving enzymes. When the OPG/RANKL/RANK system is activated, osteoclasts significantly intensify their osteolytic destruction of the bone matrix, leading to the development of BCP. Bone-tissue immune cells within the bone microenvironment of metastatic tumors—such as monocytes, macrophages, dendritic cells (DCs), and myeloid-derived suppressor cells—can also differentiate into osteoclasts [156]. Upon activation, osteoclasts increase bone resorption, releasing large amounts of H^+^ that acidify the bone microenvironment, sensitizing and activating nociceptors in sensory neurons. When osteoclasts become excessively activated, spinal neurons become abnormally activated, and the inflammatory factors they secrete further exacerbate pain and bone destruction. Therefore, inhibiting osteoclast activation is an effective means of alleviating cancer pain.

In pharmacological treatment, bisphosphonates bind to calcium in bones, forming insoluble calcium salt precipitates that inhibit osteoclast activity. This reduces acid-induced activation of primary afferent nociceptors and suppresses phenyldiphasic phosphodiesterase synthase (FPPS), exerting anti-inflammatory and analgesic effects [101]. Quercetin in Peony and Licorice Decoction inhibits osteoclast activation and reduces pain by modulating the RANKL/RANK/OPG signaling pathway and inflammatory responses [105]. Huachansu alleviates bone metastatic cancer pain by inhibiting osteoclast formation through regulating the OPG/RANK/RANKL pathway [103]. Bulleyaconitine A inhibits osteoclast differentiation and mediates bone resorption by downregulating the transcription of osteoclast activation T cell nuclear factor 1 (NFATc1) during the differentiation of BMMs, exerting analgesic effects [108]. Yishen Gukang Formula inhibits osteoclast activation by regulating the OPG/RANK/RANKL pathway, thereby suppressing bone destruction in rats with osteosarcoma-induced pain and alleviating pain symptoms [111].

## 4. Application of Drug Delivery Systems in Cancer Pain Management

**Figure 5:**
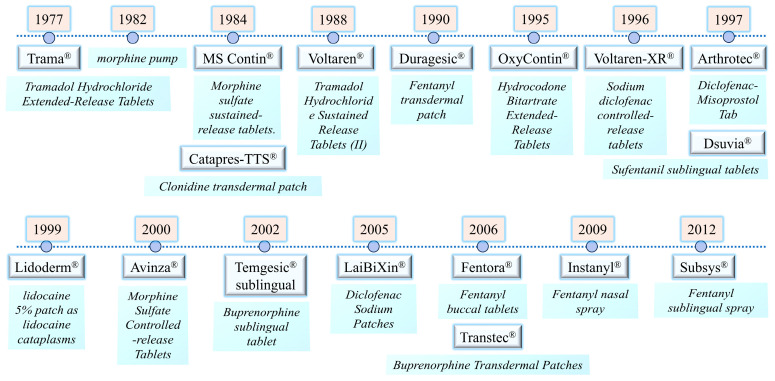
The development timeline of cancer pain medications based on delivery systems since their initial market launch. This is an original figure created by the authors using Microsoft PowerPoint.

The complexity of cancer pain management stems from its diverse pathological mechanisms, varied sites of occurrence, and distinct drug targets. Traditional administration methods often result in inadequate analgesia, significant side effects, and poor patient compliance due to pharmacokinetic fluctuations, systemic exposure, and delayed effects. To overcome these limitations, DDS have emerged. By developing controlled-release formulations to achieve steady-state plasma concentrations, designing mucosal and transdermal delivery systems to enhance convenience and onset speed, and innovating intrathecal delivery pathways for central-targeted analgesia, these precise, controllable delivery strategies are driving cancer pain management from traditional models toward personalized, high-efficacy treatment paradigms. See Table 4 and Figure 5 for details.

### 4.1. Oral Controlled-Release Drug Delivery Systems

Cancer pain, as a persistent complication throughout cancer treatment, requires long-term, controllable therapeutic approaches. While conventional oral formulations offer rapid onset of action, they present issues such as significant fluctuations in blood drug concentrations and the need for frequent dosing. Oral sustained-release formulations achieve therapeutic effects by prolonging drug release, reducing dosing frequency, and maintaining stable plasma concentrations [157]. However, release rates are susceptible to gastrointestinal conditions and carry risks of interindividual variability. Oral controlled-release formulations further optimize this system by maintaining drug concentrations within the therapeutic window, offering the advantage of prolonged release while reducing dosage and improving patient compliance [158]. Clinically available oral sustained- and controlled-release formulations include tramadol sustained-release tablets, morphine sustained- and controlled-release tablets, and oxycodone sustained-release tablets.

In 1977, Germany’s Grünenthal GmbH Pharmaceutical Co., Ltd. first introduced tramadol hydrochloride sustained-release tablets (Trama^®^) [159]. Following a 100 mg dose, peak plasma concentration (C_max_) of 141 ± 40 ng/mL was reached after 4.9 h; after a 200 mg dose, C_max_ of 260 ± 62 ng/mL was achieved after 4.8 h. As a weak opioid analgesic, the introduction of tramadol sustained-release formulations extended the drug’s duration of action, demonstrating good efficacy in treating moderate to severe cancer pain. However, its relatively low peak concentration may also imply insufficient control for breakthrough pain. To improve the pharmacokinetic profile of oral tramadol, Nippon Zoki developed a novel formulation: a dual-layer sustained-release (SR) tablet. The upper layer delivers 35% of the immediate-release (IR) dose, while the lower layer provides 65%. As an SR formulation, it is administered twice daily (Twotram^®^ tablets; Nippon Zoki Pharmaceutical Co., Ltd.). To date, Phase III clinical studies have demonstrated the efficacy of the dual-layer tramadol tablet in treating cancer pain; however, current research limitations prevent assessment of tramadol’s long-term effectiveness [160].

On 18 September 1984, USA’s Hikma Pharmaceuticals Co., Ltd. first marketed morphine sulfate sustained-release tablets (MS Contin^®^) [161] utilizing the fully dispersed permeation dissolution sustained-release technology prevalent in the 1980s. In 1988, China Southwest Pharmaceutical Co., Ltd. developed China’s first 24-h long-acting analgesic, Tramadol Hydrochloride Sustained Release Tablets (II), by adopting the skeleton-film dual-controlled-release system, and in 1994, the company developed Morphine Hydrochloride Sustained-release Tablets by adopting the new technology of solid-dispersed fusion granulation, which could realize 12-h sustained analgesia through the principle of skeleton sustained-release [162]. Morphine sulfate extended-release tablets utilize salts with sulfate anions, while morphine hydrochloride extended-release tablets employ salts with hydrochloride anions. Compared to sulfate salts, hydrochloride salts exhibit faster dissolution rates. Compared to conventional morphine tablets, morphine sustained-release tablets typically reach peak plasma concentrations 2–3 h post-administration with slightly lower peak levels. Steady-state plasma concentrations exhibit minimal fluctuation, making both formulations suitable for managing severe cancer pain. In 1995, USA’s Purdue Pharmaceuticals Co., Ltd. first launched the extended-release hydrocodone bitartrate tablet (OxyContin^®^) [163], which provides sustained release for 12 h. It has an oral bioavailability of 60~87%, is well absorbed, and reaches steady-state plasma concentrations within 24~36 h. It is indicated for the relief of persistent moderate to severe cancer pain. However, it was precisely its highly efficient controlled-release technology and potent analgesic effects that laid the groundwork for the severe abuse and addiction crisis it would later trigger in the United States and other regions.

In 2000, the USA FDA approved controlled-release morphine sulfate tablets (Avinza^®^). Based on slow release, they maintain stable blood concentrations and reduce gastrointestinal irritation. Twice-daily dosing provided excellent pain control with minimal need for rescue medication [164]. This formulation enabled a reduction of over 20% in daily morphine dosage for advanced cancer patients. Most patients reported that controlled-release formulations offered superior convenience and adequacy of relief compared to standard oral morphine tablets [165]. They provide more sustained and stable analgesic effects, allowing adjustment of release rates and doses to individual patient needs. This approach reduced the risk of addiction and enabled personalized treatment regimens.

### 4.2. Mucosal Drug Delivery System

MDDS is a novel drug delivery system that primarily involves applying drugs with suitable carrier materials to mucosal surfaces to achieve localized effects or systemic absorption into the bloodstream for systemic therapeutic action [166]. Mucosal tissues possess a rich network of capillaries and lymphatic vessels. Certain mucosal sites, such as the nasal cavity and oral cavity, can bypass the first-pass effect, enhancing drug bioavailability. Furthermore, the limited variety and quantity of enzymes within mucosal tissues result in relatively low enzymatic degradation of drugs. Local administration thus improves bioavailability and patient compliance [167].

#### 4.2.1. Oral Mucosal Drug Delivery

Oral mucosal DDS refer to a method where drugs are absorbed through the oral mucosa and enter the systemic circulation to exert their therapeutic effects. Compared to traditional oral administration, the oral mucosal delivery system allows drugs of specific molecular sizes to pass through the oral mucosa, directly entering the jugular vein via oral veins before entering systemic circulation. This bypasses degradation by gastrointestinal pH and enzyme systems and avoids hepatic first-pass metabolism, significantly enhancing drug bioavailability [168]. Clinically available formulations for oral mucosal administration include fentanyl sublingual tablets, orally disintegrating films, and sublingual sprays.

Fentanyl (SFC), a highly lipophilic opioid, exhibits rapid mucosal absorption with high bioavailability. The sublingual tablet and orally disintegrating film developed by Cephalon Co., Ltd., received FDA approval in 1997 and 2006, respectively. The fentanyl sublingual tablet is particularly indicated for BTP. Jordi [169] treated 127 elderly BtCP cancer patients with sublingual fentanyl, which significantly reduced VAS scores at 30 min after administration, with effectiveness in terms of overall and maximal pain relief of 34.7% and 30.6%, respectively. Fentanyl Buccal Soluble Film (FBSF) consists of a double-layer polymer membrane comprising an adhesive layer containing fentanyl and an inactive layer that prevents the diffusion of fentanyl. The adhesive layer can adhere to moist mucosal surfaces within seconds. When placed in the oral cavity, the drug is absorbed through the oral mucosa into the bloodstream, representing a novel delivery form for rapid relief of BtCP [170]. Yi-Hao Chiang [171] conducted a 14-day observation using FBSF. Among 63 BtCP episodes, 47.6%, 73.0%, 84.1%, and 92.0% of BtCP episodes showed significant relief within 5, 10, 15, and 30 min after FBSF administration, respectively. with BtCP intensity reduced by 37.8%, 47.6%, 73.0%, and 84.1%, respectively, and pain scores decreased by 2.3 points, 3.3 points, 4.1 points, and 4.9 points, respectively, demonstrating rapid and effective reduction in BtCP pain intensity. Fentanyl sublingual spray (Subsys^®^), marketed in the United States in 2012 for improving the rate and extent of fentanyl absorption through the sublingual mucosa [172], provides rapid pain relief for opioid-tolerant cancer patients and helps with acute or postoperative pain relief [173]. It reduces pain intensity within 4 min after administration and lasts up to 60 min, with significant efficacy in BtCP treatment [174].

#### 4.2.2. Nasal Mucosal Drug Delivery

Nasal mucosal drug delivery refers to a noninvasive drug delivery method where medications are absorbed through the nasal mucosa, characterized by rapid absorption, fast onset of action, and high drug concentration, enabling both local and systemic therapeutic effects [175]. Opioid drugs administered nasally exert rapid effects on the CNS, providing swift analgesia. Fentanyl nasal spray (Instanyl^®^), developed by Nycomed in 2009 and approved by the European Medicines Agency, is indicated for BTP [176]. The nasal spray delivery device atomizes fentanyl into a fine mist that forms a gel on the mucosa, allowing the active ingredient to be absorbed through the mucosa into the bloodstream. Compared to oral morphine or oral fentanyl formulations, it provides more effective pain relief lasting 15~45 min [177]. Plasma concentrations are significantly higher than with oral mucosal administration, achieving a bioavailability of up to 89%, with analgesic effects occurring within approximately 5 min [178]. Not only does it provide rapid analgesic effects, but it also avoids the gastrointestinal side effects associated with oral administration, making it another effective option for treating BTP.

### 4.3. Transdermal Drug Delivery System

TDDS represents an innovative drug delivery method that administers medications at a constant rate through the skin into the systemic circulation, enabling targeted effects for local or systemic therapy. This approach offers advantages such as circumventing the first-pass effect of oral medications, enhancing and sustaining blood drug concentrations, preserving drug potency, and improving patient compliance [179]. Therapeutic agents accumulate in specific skin layers where they permeate and enter the bloodstream during absorption. Compared to traditional systems, this approach permits the storage of small quantities of nanotechnology-developed drugs within transport and delivery structures [20].

**Figure 6:**
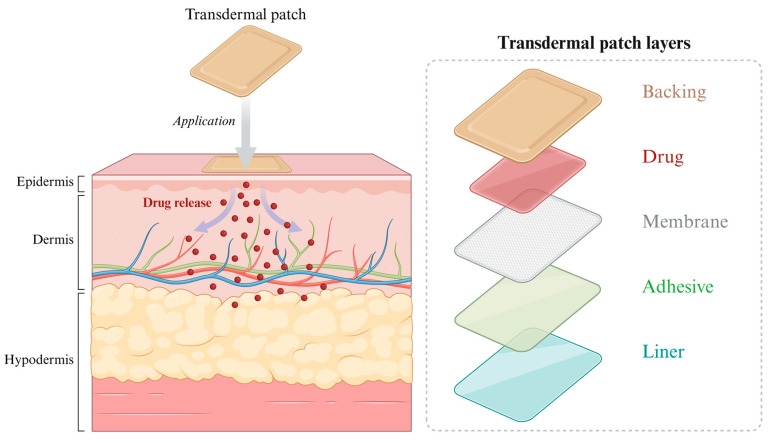
Transdermal Patch Therapy Pathway. Created in BioRender. Li, B. (2025) https://BioRender.com/84xkqvh.

Transdermal patches (Figure 6) serve as the preferred treatment for patients unable to swallow or those with poor tolerance or compliance to oral medications. They offer advantages including avoidance of hepatic first-pass metabolism, bypassing gastrointestinal metabolism, prolonged duration of action, and stable blood drug concentrations [180]. In the field of cancer pain management, transdermal patches containing opioids (such as fentanyl, buprenorphine, diclofenac sodium, lidocaine, and clonidine) represent the most clinically mature and widely adopted technology.

As early as 1984, the Clonidine adhesive patch (Catapres-TTS^®^) was first launched in the United States. A randomized controlled trial confirmed its analgesic efficacy for cancer pain, though it is more suitable for specific patient populations [181]. Fentanyl-based drugs exhibit excellent membrane permeability due to their low molecular weight and high lipophilicity. In 1990, the U.S. FDA first approved the fentanyl transdermal patch (Duragesic^®^), with current technologies including reservoir-type and matrix-type formulations [182]. Yong Liu [183] treated 267 patients with moderate-to-severe cancer pain using a low-dose fentanyl transdermal patch (TDF) at 12.5 μg/h. The mean effective therapeutic dose was 6.3 ± 6.08 mg, achieving a total pain relief rate of 98.12% with mild adverse reactions. Shigeki Yamaguchi [184] found TDF achieved an 87.0% efficacy rate in opioid-naive cancer pain patients, with a mean VAS pain score change of −34.5 mm and no serious respiratory depression events.

The 5% lidocaine transdermal patch (Lidoderm^®^), developed and manufactured by the US subsidiary of Japan’s Imperial Pharmaceutical Co., Ltd. was launched in the U S in March 1999 for use in various forms of local anesthetic pain relief [185,186]. Jui-Hung Tsai [187] applied it to 96 advanced cancer patients already receiving opioid therapy. On the first and second days, 51 patients experienced reduced NRS pain intensity, indicating its efficacy in alleviating cancer-related local and superficial neuropathic pain. In 2005, China’s Bengbu Fengyuan Tushan Pharmaceutical Co., Ltd. launched a patch containing 50 mg of sodium diclofenac (Laixin^®^) for local pain relief [188] However, cancer pain management requires stable blood concentrations and systemic effects.

In 2006, Luye Pharma’s German subsidiary first launched the transdermal transect patch (Transtec^®^). As a potent opioid analgesic, it prolongs analgesic effects by activating μ-opioid receptors, with sustained release lasting up to 7 days. It is excreted via feces and exhibits no dose-dependent upper limit for analgesic efficacy, though respiratory depression has an upper limit. Its clinical use remains relatively limited.

### 4.4. Intrathecal Targeted Drug Delivery

**Figure 7:**
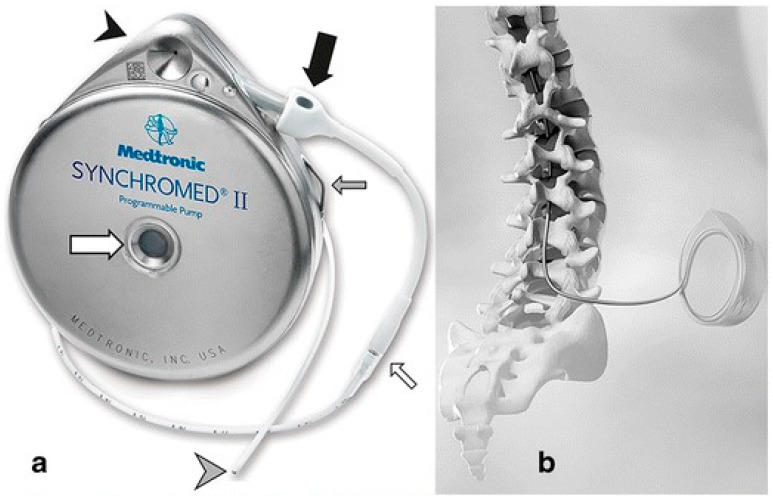
Implanted, programmable pump system. External view (**a**) and drawing of implanted pump and intrathecal catheter 8731SC (**b**). Pump with the catheter access port (black arrowhead), pump catheter connection (thick black arrow), refill membrane (thick white arrow) and suture loops for fixation (thin grey arrow), catheter-catheter segment connection (thin white arrow) and titanium catheter end (grey arrowhead). Reproduced with permission from reference. *Insights into imaging* vol. 8,5 (2017) [191]. Creative Commons Attribution 4.0 International License. Copyright © The Author(s) 2017.

ITDS proves effective for various neuropathic, nociceptive, and mixed pain conditions in cancer patients. By injecting analgesic drugs into the subarachnoid space via microcatheters, the cerebrospinal fluid circulation enables direct action of the medication on the CNS, treating chronic cancer pain and refractory cancer pain [189]. Compared to conventional administration methods, ITDD has been demonstrated to be superior to comprehensive medical management in pain control and reducing drug-related toxicity [190]. Intrathecal drug delivery system (IDDS) was employed as early as the 1980s for refractory pain management. Commonly used delivery devices include semi-implantable and fully implantable systems. Semi-implantable systems feature a catheter connected to an infusion port, with medication delivery controlled via a dedicated external medication reservoir and a patient-controlled analgesia (PCA) pump. Fully implantable systems connect catheters to programmable implantable pumps. The entire system is buried subcutaneously, with medication stored in the subcutaneous pump. Infusion parameters can be remotely adjusted via a programmer, making it the most rigorously tested and widely used implantable programmable device globally to date (Figure 7) [191].

Depending on the location and extent of the patient’s pain, the IDDS catheter can be placed within the spinal canal, the greater occipital cistern, or even the ventricles. Positioning the tip of the intrathecal morphine pump catheter in the anterior pontine cistern effectively relieves refractory head and facial cancer pain with extremely low morphine doses [192], reducing opioid usage in patients with refractory cancer pain [193]. Placing medication within the subarachnoid space allows for delivery near the trigeminal nerve, treating craniofacial cancer pain while reducing opioid intake and alleviating side effects [194].

Clinically, pain management often involves combining different medications such as morphine, hydromorphone, ropivacaine, fentanyl, and dexmedetomidine based on the patient’s analgesic goals. Among these, morphine is the preferred opioid for chronic pain IDDS. Its high hydrophilicity, slow onset, and prolonged duration of action allow it to diffuse more rapidly than other opioids after injection, providing broader analgesic coverage. It was the earliest IDDS drug clinically used for cancer pain management. Typically, 1 mg of morphine via IDDS is equivalent to 10 mg epidural, 100 mg intravenous, or 300 mg oral administration. Furthermore, different combination therapies can reduce morphine dosage while enhancing analgesic efficacy [195].

## 5. The Development Prospects of Drug Delivery Systems in Cancer Pain Management

### 5.1. Transdermal Drug Delivery System

#### 5.1.1. New Transdermal Patch

Traditional transdermal patch delivery is limited by the barrier function of the skin’s stratum corneum, resulting in low transdermal absorption efficiency [196]. In the development of novel transdermal patches, research focuses on optimizing drug release and enhancing transdermal efficiency.

For example, in 2020, Chen Shen [197] developed a 5% lidocaine gel patch by uniformly mixing lidocaine with a hydrophilic polymer gel matrix and coating it onto a backing material. This formulation offers advantages including ease of use, rapid onset of action, and high safety.

In 2021, Japan’s Hisamitsu Pharmaceutical Co., Ltd. launched a novel diclofenac sodium patch (Zicthoru Tapes^®^) containing 75 mg of active ingredient, delivering systemic analgesia upon application [198]. In a phase III multicenter, randomized, double-blind, placebo-controlled comparative study, once-daily diclofenac sodium patches were initiated at 150 mg/day (2 patches) and could be increased to 225 mg/day (3 patches). At steady state, repeated administration of 225 mg yielded C_max_ and AUC values of 294.16 ng/mL and 3052.2 ng·h/mL, respectively, representing 76% of the relative bioavailability of oral diclofenac sodium 100 mg/day. This demonstrates sustained efficacy of the novel diclofenac sodium patch in cancer pain management. All adverse events were consistent with those observed with existing diclofenac sodium formulations during cancer treatment [199].

In 2024, Sourav Adhikary [200] developed a novel clonidine hydrochloride/montmorillonite sodium (CH/Na-MMT) composite patch. Na-MMT enables sustained drug release over four weeks, with clonidine achieving 82% release at pH 7.4. In rats, it demonstrated sustained analgesia. The CH/Na-MMT patch effectively penetrated skin, reaching effective blood concentrations of 100 mg/kg. Drug loading efficiency was 84.98 ± 0.31%, loading capacity was 6.78 ± 0.17%, and transdermal flux (Jss) was 0.76 ± 0.11 μg/cm^2^·h. CH/Na-MMT prolongs drug release duration, showing promise for cancer pain management by reducing dosing frequency, enhancing therapeutic efficacy, and improving patient compliance.

#### 5.1.2. Microneedle

As a third-generation transdermal delivery system, microneedles (MNs) utilize micron-sized needles to penetrate the skin’s stratum corneum, creating microchannels that allow analgesic drugs to directly enter the epidermis or dermis. This bypasses the barrier function of the stratum corneum, delivering drugs directly to pain sites such as tumor peripheries or areas of nerve compression. By establishing high local drug concentrations, MNs achieve more effective analgesia [201]. Since the drug acts directly at the local site, it reduces systemic distribution, avoids first-pass hepatic metabolism and gastrointestinal side effects, and improves drug utilization.

**Figure 8:**
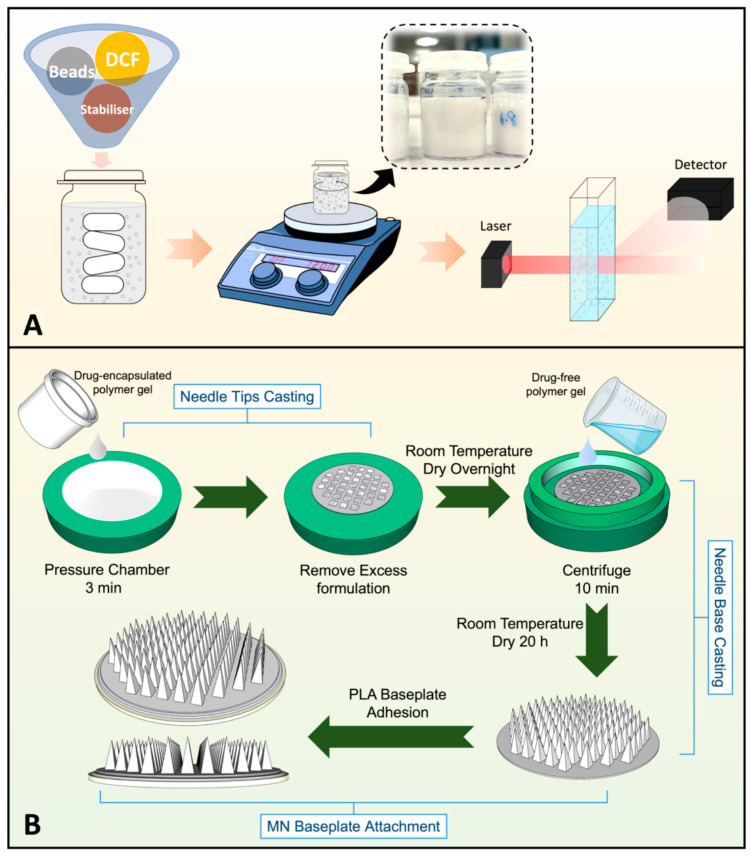
(**A**) Fabrication of DCF nanosuspensions using a wet bead milling approach. (**B**) Manufacture of trilayer MN patches loaded with drug. Reproduced with permission from reference. *Biomaterials advances* vol. 161 (2024) [202]. Creative Commons Attribution 4.0 International License. Copyright © 2024 The Author(s). Published by Elsevier B.V. All rights reserved.

For example, Mingshan Li [202] prepared diclofenac sodium (DCF) NPs with an average diameter of 202.63 ± 2.02 nm via wet milling. These were stabilized using polyvinyl alcohol (PVA) and polyvinylpyrrolidone (PVP) (35% *w*/*w*, PVA: PVP = 1:2) to stabilize the suspension. The nanoparticle suspension was then freeze-dried to ensure nanoparticle stability and high drug loading capacity. The MN patches were produced using a mold-casting method (Figure 8). Skin penetration capabilities of different MNs were validated through mechanical strength testing (compression experiments) and in vitro insertion experiments (Parafilm^®^ model). Results showed that the 600-needle array with longer needles achieved a drug loading capacity of 2.33 mg, demonstrated superior skin insertion depth, and significantly increased drug deposition. Finally, combined in vivo and in vitro evaluations demonstrated that DCF-NPs MNs exhibited a significantly longer in vivo half-life (T_1_/_2_ = 8.7 h) compared to oral administration (T_1_/_2_ = 1.9 h) with drug delivery efficiency (34%) superior to that of conventional transdermal DCF gel (6%). The current study showed that a single application of DCF-NP MNs could provide up to 48 h of systemic delivery and up to 72 h of localized and targeted drug concentrations. This fully demonstrates the tremendous potential of MNs, particularly those incorporating nanotechnology, in enhancing transdermal drug delivery efficiency and prolonging therapeutic duration.

Additionally, Ze Qiang Zhao [203] designed a hydrogel microneedle (MN) system made of gelatin-methacrylate (GelMA) for sustained delivery of lidocaine hydrochloride (LiH). The use of a backing layer reservoir significantly enhanced the drug loading capacity of GelMA MNs, increasing it substantially from 50–300 μg to 2927.168 ± 174.408 μg. In vivo and in vitro drug release experiments compared LiH/GelMA MNs with commercial LiH patches, demonstrating that MNs substantially enhanced drug absorption rates. Additionally, a rat SNI model was established to assess behavioral pain sensitivity to mechanical stimulation. Twenty-four hours post-treatment, rats exhibited a more than threefold increase in mechanical pain threshold compared to controls, indicating the excellent sustained analgesic efficacy of LiH/GelMA MNs. Furthermore, biosafety assessments in rats revealed that the MNs’ application site returned to normal appearance within hours post-treatment, with no dermatological side effects or behavioral abnormalities observed throughout the study. The findings demonstrate that MNs not only enhance the transdermal absorption efficiency of lidocaine but also enable sustained analgesia with high lidocaine loading, offering a convenient, safe, and effective novel approach for treating localized chronic pain.

### 5.2. Disposable Intrathecal Drug Delivery

Given the multifaceted mechanisms underlying cancer pain, there has been a surge in the development of intrathecal targeted drugs addressing multiple levels, including pain-related receptors, associated enzyme activities, chemokines in pain transmission, protein-coding genes, and targeted natural small molecules.

#### 5.2.1. Targeting Relevant Receptors

These substances directly bind to specific receptors on cell membranes or nuclei, thereby activating or deactivating downstream signaling pathways to alleviate cancer pain. For example, the neuromediator U receptor 2 (NMUR2) is widely distributed throughout the CNS, with the highest concentrations in the cerebral cortex and hypothalamus, playing a crucial role in nociception and inflammation [204]. Intrathecal injection of si-NMUR2 significantly reduces the expression level of the target NMUR2, participating in pain regulation through the PKC/ERK and PI3K/AKT signaling pathways [205]. Peroxisome proliferator-activated receptor gamma (PPAR-γ) is a key receptor involved in regulating nuclear factor kappa-B (NFκB) [206]. Intrathecal injection of rosiglitazone (a PPAR-γ agonist) and GW9662 (a PPAR-γ antagonist) activates PPAR-γ to inhibit the NF-κB/NLRP3 inflammatory axis in spinal neurons, thereby alleviating cancer bone pain [207].

#### 5.2.2. Targeted Enzymes

These agents modulate intracellular biochemical reactions and signaling pathways by enhancing or inhibiting specific enzyme activities, thereby alleviating cancer pain. For instance, histone deacetylases (HDACs) play a crucial role in regulating glial cell-mediated immune responses; suppressing HDAC expression in the spinal cord alleviates BCP. Intrathecal injection of the HDAC inhibitor SAHA suppresses HDAC and GSK3β activity, thereby alleviating BCP [208]. SIRT1, an NAD+-dependent HDAC, has been reported to play a key role in BCP and is important in neuropathic pain by regulating inflammatory responses, oxidative stress, the immune system, and epigenetic modifications [209]. Intrathecal injection of the SIRT1 activator SRT1720 suppresses mGluR1/5 expression and activates SIRT1 [210], thereby alleviating BCP via the CREB/CRTC1 signaling pathway [211].

#### 5.2.3. Targeted Chemokines

These substances precisely regulate the migration and localization of immune cells within the body, representing a hotspot in immunology and cancer research. Monocyte chemotactic protein-1 (formerly MCP-1, now designated CCL2) and its primary receptor, C-C motif chemokine receptor 2 (CCR2), constitute one of the most characteristic neuroactive chemokine pairs regulating nociception. They have been demonstrated to play critical roles in nociceptive processing under chronic pain conditions, mediating neuroinflammation, neuron-glia interactions, and enhancing synaptic transmission in the dorsal spinal cord horn [212]. Intrathecal injection of the CCR2-selective agonist PP101 reduces T-cell infiltration and neuronal activation in DRG, effectively alleviating neuropathic pain and BCP by inhibiting the CCL2/CCR2 signaling pathway [213]. The chemokine C-X-C motif ligand 12 (CXCL12), belonging to the C-X-C subfamily, exerts its effects by binding its specific receptor, C-X-C motif receptor 4 (CXCR4) [214]. Increased expression of the CXCR4 signaling pathway in spinal neurons activates the RhoA/ROCK2 signaling pathway, promoting pain hypersensitivity. Intrathecal injection of the CXCR4 inhibitor Plerixafor (AMD3100) and the ROCK2 inhibitor Fasudil blocks the CXCR4-RhoA/ROCK2 pathway [215].

#### 5.2.4. Targeted Protein-Coding Genes

MicroRNAs (miRNAs) play a crucial role in regulating the expression of pain-related mRNAs and proteins. Pain can induce the upregulation or downregulation of multiple miRNAs, participating in the regulatory mechanisms of chronic pain and the acute nociception process [216]. Among these, miR-195 is a key member of the microRNA-15/16/195/424/497 family [217]. Intrathecal injection of a miR-195 inhibitor reduces miR-195 expression while elevating MPT and cold stimulus thresholds, increasing expression of autophagy-related proteins in mice, suppressing neuroinflammation, and alleviating neuropathic pain [218]. In recent years, bone marrow-derived mesenchymal stem cells (BMSCs) have emerged as a promising cell therapy due to their well-established properties, including differentiation into neural cells, migration to injury sites, and immunosuppressive functions [219]. Studies indicate that genetically modified BMSCs significantly suppress pain perception [220]. Molecular alterations induced by miR-9-5p overexpression confer potent analgesic properties to BMSCs. Thus, intrathecal injection of miR-9-5p-modified mouse BMSCs modulates MOR expression by targeting REST, thereby suppressing inflammatory responses and alleviating BCP [221]. Additionally, intrathecal injection of miR-199a-3p agomir directly targets DNMT3A to influence Nrf2 expression, thereby exerting therapeutic effects against BCP [222].

#### 5.2.5. Targeted Natural Small Molecules

Liquiritin, derived from the TCM licorice, exhibits broad-spectrum effects unlike synthetic drugs with single targets. It exerts anti-inflammatory and antioxidant actions through multiple pathways, reflecting the multi-target synergistic characteristics of natural products. Intrathecal injection of different doses of glycyrrhizin effectively suppressed activation of the CXCL1/CXCR2 signaling pathway and production of IL-1β and IL-17 in BCP rats. It also inhibited activation of CXCL1 in spinal astrocytes and CXCR2p in neurons, alleviating BCP in rats [223].

### 5.3. Nanodrug Delivery Systems

NDDS utilizes nanomaterials to encapsulate drugs and deliver them to target sites, enabling precise targeting and controlled release. This approach enhances local drug concentration, reduces systemic side effects, prolongs sustained drug release [224], and mitigates adverse reactions caused by analgesics [225]. Compared to existing clinical pain medications, it achieves more sensitive and targeted treatment with fewer side effects [226]. Although no related drugs have been marketed, nanomedicine delivery systems such as liposomal NPs, polymeric NPs, magnetic NPs, co-delivery NPs, and carrier-free self-assembled NPs offer novel management approaches for cancer pain treatment. Specific details are provided in Table 5.

#### 5.3.1. Liposome Nanoparticles

Local anesthetics are widely used in the treatment of acute and chronic pain. However, their analgesic effects last only a few hours due to their short half-life, limiting their application in cancer pain management [236]. Nevertheless, encapsulating local anesthetics in lipid-based nanocarriers offers advantages such as slow release, prolonged duration of action, and low toxicity to the central nervous and cardiovascular systems [237]. Jiqian Zhang [227] prepared ropivacaine-loaded liposomes (Rop-DPRL) via the thin-film hydration method, achieving a cumulative release rate of 71.4% over 24 h. When administered via intraperitoneal injection concurrently with caloric restriction (CR), this formulation exhibited dual inhibition of STAT3 phosphorylation and reduced vascular endothelial growth factor A (VEGF-A) levels. Repeated administration of Rop-DPRL combined with CR effectively suppressed tumor-induced cancer pain in a mouse model with tumors implanted near the sciatic nerve, achieving rapid analgesia. Additionally, Bahrami MA [228] encapsulated citrate fentanyl within nanostructured lipid carriers and synthesized NPs via thermal homogenization, achieving an 82% encapsulation rate and enabling 72-h sustained release. Under identical conditions, the lipid nanocarriers reduced the required drug dose by 50% while providing analgesic effects, potentially lowering the risk of citrate fentanyl overdose. However, further work is needed to define the therapeutic margin of this drug.

#### 5.3.2. Polymeric Nanoparticles

Nano-controlled release microspheres represent a specific structural and functional type within polymeric NPs. They enable precise regulation of drug release, enhance drug targeting, improve the permeability of hydrophobic drug molecules across cell membranes, enhance drug stability, and modify delivery routes [238]. Encapsulating drugs within microspheres prolongs therapeutic effects. By modulating analgesic release rates, therapeutic blood concentrations can be maintained within therapeutic ranges while reducing fluctuations. Yan Zhou [229] prepared morphine sulfate nano-controlled release microspheres via an emulsification-crosslinking method. At a crosslinker ratio of 8:1, the highest drug loading capacity and encapsulation efficiency were achieved, reaching 15.1% and 54.0%, respectively. with drug release rates of 74.6% and 99.8% at 4 h and 72 h, respectively. These microspheres demonstrated excellent in vitro drug release characteristics, reduced NE levels in the hypothalamus of viscerally painful mice, promoted 5-HT synthesis and secretion, effectively decreased CNS pain sensitivity, alleviated tumor-induced visceral pain, elevated pain thresholds, prolonged pain relief duration, and exhibited favorable safety profiles. Additionally, protease-activated receptor 2 (PAR2) is a G protein-coupled receptor (GPCR) mediating oral cancer pain. Divya Bhansali [230] developed a polymeric nanoparticle encapsulating the PAR2 inhibitor AZ3451, with encapsulation rates ranging from 95.8% to 99.6%, enabling sustained release over 24 h. These NPs simultaneously target endosomal signaling in the TME and neurons. These findings indicate that PAR2 on oral cancer cells and neurons participates in oral cancer pain. Compared to free drugs, NPs loaded with PAR2 antagonists provide higher analgesic effects and improved oral function.

#### 5.3.3. Magnetic Nanoparticles

In recent years, magnetic NPs (MNPs) have emerged as a research hotspot in targeted pain relief due to their controllable magnetic responsiveness, high drug loading capacity, and biocompatibility [239]. However, most MNPs studies still rely on non-green synthesis processes, such as chemical reduction methods, which may pose biosafety risks [240]. Xiaoli Lv [231] pioneered the integration of green synthesis strategies with transdermal delivery systems for magnetic NPs (MNPs-TDDS), developing folic acid-targeted magnetic nanocomposites (catHEC·FA@SPIO) using aqueous coprecipitation and a biocompatible polymer coating (hydroxyethyl cellulose), demonstrating its significant advantages in nasopharyngeal cancer pain management through multidimensional clinical metrics. The study showed a cumulative release rate of 82.4 ± 3.1% at pH 5.5 over 72 h, exceeding traditional liposomes by 60%. At a magnetic field strength of 0.5 Tesla (T), the drug penetration depth in tumors was only 0.006 cm; however, at magnetic field strengths of 1.5 T and 2.5 T, the penetration depth increased to 0.071 cm and 0.192 cm, representing 11.8-fold and 32-fold enhancements, respectively. Furthermore, the average drug concentration within tumors significantly increased with magnetic field strength, indicating that high-intensity magnetic fields enhance drug accumulation and efficacy in deep-seated tumors. This not only offers a novel strategy for cancer pain management but also lays the foundation for the application of MNPs in chronic diseases.

#### 5.3.4. Size-Tunable Nanoparticles

The absolute quantity and size of NPs significantly influence drug diffusion and retention properties within tumor tissues. These factors not only regulate drug distribution within tumors but are also directly correlated with local tumor ablation efficacy. Yu Tan [232] synthesized size-controlled HA-As_2_S_3_ NPs using sodium hyaluronate (HA) of varying molecular weights as a supramolecular scaffold. HA first coordinates with arsenic ions (As^3+^) to form HA-As^3+^ framework intermediates of varying sizes. These HA-As^3+^ intermediates subsequently reacted with sulfide ions to form size-controlled HA-As_2_S_3_ particles (HA-As_2_S_3_ NPs). This approach enables precise control over As_2_S_3_ particle size and provides a simple, environmentally friendly method for synthesizing size-controlled metal sulfide NPs. The synthesized HA-As_2_S_3_ NPs exhibited excellent stability. The combination of hydrogen sulfide (H_2_S) gas therapy and locally targeted interventional ablation may represent a promising approach for eradicating localized cancer pain. This study employed ultrasound-guided interventional therapy to directly inject HA-As_2_S_3_ NPs in situ into mouse tumors, ensuring precise nanoparticle delivery. This achieved safe tumor ablation and effective cancer pain relief. The released H_2_S gas and As^3+^ achieve targeted tumor cell treatment within the acidic TME, significantly enhancing mitochondrial damage and ROS accumulation. This effectively induces tumor cell apoptosis while markedly alleviating cancer pain, demonstrating favorable biosafety.

#### 5.3.5. Co-Delivery Nanoparticles

**Figure 9:**
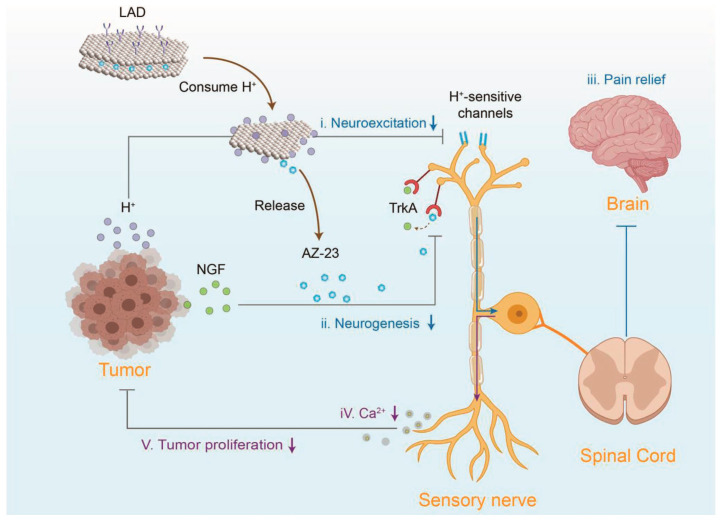
Schematic diagram of nerve–cancer crosstalk blocking strategy induced by LAD for metastatic bone cancer pain treatment. (**i**) LAD consumed the excessive H^+^ to inhibit the activation of H^+^-sensitive channels, thus decreasing the excitation of sensory nerves. (**ii**) AZ-23 was released from the degraded LAD that blocked the NGF/TrkA pathway to avoid neurogenesis and subsequent pain sensitization. (**iii**) Suppressing neuroexcitation and neurogenesis prevented pain formation, resulting in pain relief. (**iv**) As positive feedback of pain relief, the Ca^2+^ concentration was downregulated, (**v**) further restraining the Ca^2+^-crosstalk-induced cancerous cell cycle change, and finally inhibiting the tumor proliferation. Reproduced with permission from reference. *Advanced materials (Deerfield Beach, Fla.)* vol. 34,17 (2022) [233]. Creative Commons Attribution 4.0 International License. Copyright © 2022 Wiley-VCH GmbH.

The treatment of cancer pain requires multi-targeted interventions. Co-delivery NPs serve as nanoscale carriers capable of simultaneously loading and delivering two or more distinct therapeutic or diagnostic agents, achieving synergistic effects. Xu Chu [233] loaded the antagonist AZ-23 and the external modulator alendronate sodium (ALD) within a layered double hydroxide (LDH/AZ-ALD, abbreviated as LAD) nanoscale carrier. This Mg/Al layered double hydroxide nanoscale shell (Mg/Al-LDH) achieved neuro-antagonistic effects by simultaneously inhibiting the N-acetylaspartate (NAA) transporter and modulating the activity of the N-acetylaspartate receptor. AZ-23 and externally modifying Mg/Al layered double hydroxide nanocapsules (Mg/Al-LDH) with ALD achieved a neuro-cancer crosstalk blockade strategy for metastatic BCP. As anticipated, LAD precisely targeted bone cancer sites under ALD guidance. H^+^, the primary pain-inducing factor in BCP (pH ≈ 4.5). Concurrently, Mg^2+^’s role in promoting osteoblastic differentiation protects bone from further damage. Furthermore, AZ-23 released upon LDH degradation fundamentally inhibits cancer-induced neurogenesis by blocking the NGF/TrkA pathway, thereby preventing the development of hyperalgesia and allodynia. All these actions collectively reduce neuronal excitability and alleviate pain. As a positive feedback loop, decreased neuronal excitability directly diminishes neural influence on cancer progression. It significantly inhibits tumor growth through Ca^2+^-dependent cell cycle events, suppresses activation of TRPV1/ASIC channels in pain-sensing neurons, releases NGF/TrkA pathway antagonists, and blocks nerve hyperplasia and tumor proliferation, thereby treating bone metastatic cancer pain (Figure 9).

**Figure 10:**
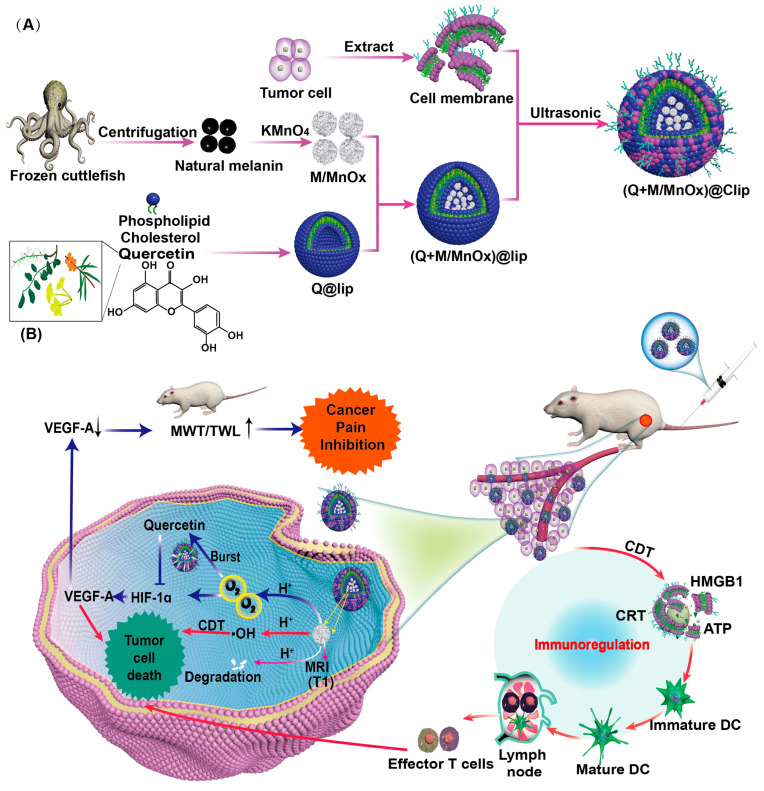
Schematic illustration of this system. (**A**) Synthesis diagram of oxygen-producing biomimetic nano-herb delivery system (Q+M/MnOx)@Clip. (**B**) Schematic of (Q+M/MnOx)@Clip for potentiated cancer therapy and pain mitigation in vivo. Reproduced with permission from reference. *International journal of nanomedicine* vol. 20 7113–7132. 31 May. 2025 [234] Creative Commons Attribution 3.0 International License. Copyright © 2025 Cao et al.

The development of co-delivery bionic nanotechnology provides insights and momentum for the modernization of TCM, offering new opportunities for cancer pain management [241]. Dian-Chao Cao [234] developed an oxygen-producing bionic nano-herbal delivery system named (Q+M/MnOx)@Clip. This system fuses membranes from TNBC 4T1 cells targeted for tumor delivery with liposome membranes, synthesizing natural melanin—which induces immunogenic cell death (ICD)—via one-step biomineralization with potassium permanganate to produce melanin/MnOx (M/MnOx) hybrids. Quercetin (Q), used for tumor suppression and pain relief, is encapsulated within the lipid bilayer of the nano-herbal delivery system, with M/MnOx integrated into its hydrophilic core. Following cancer cell recognition and internalization, the nanodelivery system interacts with endogenous H_2_O_2_/H^+^ to generate O_2_. This oxygen production induces internal pressure and oxidative stress, leading to disruption of the (Q+M/MnOx)@Clip nanocarrier’s biomimetic cancer cell membrane coating. This disruption facilitates subsequent release of MnOx and other therapeutic agents from the system. This process alleviates hypoxic and acidic conditions within the immunosuppressive TME and rapidly triggers the rupture of the nanosystem within cancer cells, ensuring efficient release of the loaded drugs (Figure 10). This innovative system adeptly performs CDT and triggers DC activation while enhancing the body’s immune system, ultimately leading to cancer cell death. Furthermore, the T1-weighted imaging capability of (Q+M/MnOx)@Clip facilitates innovative imaging-guided precision cancer therapy. The system also significantly reduces VEGF-A levels, paving the way for alleviating cancer-related pain.

#### 5.3.6. Carrier-Free Self-Assembled Nanoparticles

The self-assembly of molecular components is emerging as a common strategy for designing and synthesizing nanomaterials in biomedical applications. Molecular synergy is becoming a key indicator in both natural and synthetic self-assembled nanostructures [242]. Pengfei Zhang [235] developed a nanomaterial-mediated microknife (NP-MK) ablation for interventional ablation therapy (IAT), aimed at locally eliminating cancer pain and tumors. The IAT system employs nanoprecipitation to self-assemble Paclitaxel (PTX) and Hematoporphyrin (HP) at a 4:1 mass ratio into a carrier-free nanomedicine (PTX-HP nanomedicine) with 63% drug loading. Combining PTX and HP via self-assembly addresses their poor water solubility and generates potent synergistic anticancer and analgesic effects. Combining infusion catheters, puncture needles, syringe pumps, and empirical tumor ablation formulations, the PTX-HP nanodrug is directly infused into tumors under imaging guidance via puncture needles and syringe pumps. Assisted by the targeting ligand HP, the NP-MK system eliminates localized cancer pain. Following IAT treatment, pain-related symptoms in mice completely resolved. Thus, NP-MK technology offers an alternative pathway for expanding the application scope of nanomedicines. By adopting approaches similar to the NP-MK system, various nanocarriers will flourish in the field of precise intra-tumoral therapy.

## 6. Discussion

Cancer pain is one of the most common and dreaded symptoms experienced by patients with malignant tumors, severely impacting their quality of life and adherence to anticancer treatments. Since the WHO introduced the “Three-Step Pain Management Guidelines for Cancer” in 1986, this protocol has become the cornerstone of global cancer pain management. However, its limitations have become apparent during long-term use, particularly the tolerance and addiction associated with opioid medications, along with a range of adverse effects such as constipation, nausea and vomiting, and respiratory depression, which limit its clinical utility [243].

Against this backdrop, the integrated Chinese and Western medicine approach to cancer pain management demonstrates immense potential and unique advantages. The combination of multiple Chinese herbal formulas and proprietary Chinese medicines with standard opioid medications can maintain or even enhance overall analgesic efficacy while significantly reducing the average daily opioid dosage, decreasing the frequency of breakthrough pain episodes, and alleviating adverse drug reactions such as dizziness, constipation, nausea, and vomiting. Furthermore, the integrated strategy—combining Western medicine’s blockade of core pain pathways with Chinese medicine’s regulation of the TME, suppression of neuroimmune inflammation, and restoration of bone homeostasis—provides the pharmacological basis for achieving “enhanced efficacy with reduced toxicity” in combined TCM-Western medicine treatment. However, many current clinical studies suffer from small sample sizes, inadequate research design rigor, and short follow-up periods, resulting in low-grade evidence that struggles to gain widespread adoption in international guidelines. Additionally, the “multi-component, multi-target” nature of TCM, while an advantage, also poses significant research challenges. Most current studies on the mechanisms of action of compound formulas remain confined to whole-animal experiments and limited pathway validation. Systematic and in-depth elucidation of how these formulas precisely regulate the neuro-immune-TME network, and which specific active components play key roles, remains lacking. Furthermore, standardized guidelines for clinical application are absent. Therefore, it is crucial to design and conduct large-scale, multicenter, randomized, double-blind, placebo-controlled clinical trials. By integrating cutting-edge technologies such as systems pharmacology, network pharmacology, and metabolomics, we must deeply analyze the effective component groups, target networks, and synergistic mechanisms of TCM formulas. This will enable us to explain their “synergistic efficacy and reduced toxicity” principles using modern scientific language. Establishing standardized clinical pathways and expert consensus/guidelines is particularly important [244].

The pathogenesis of cancer pain involves complex regulation between the peripheral and CNS. Modern research reveals that its mechanisms primarily include tumor cells and infiltrating immune cells releasing large amounts of inflammatory mediators and nociceptive substances such as IL-1β, IL-6, TNF-α, PGE2, NGF, and ATP, which continuously stimulate and sensitize peripheral nociceptors, leading to peripheral sensitization; Upregulation or abnormal activation of TRPV1, VGSCs, and calcium channels significantly lowers neuronal excitation thresholds, resulting in spontaneous pain and hyperalgesia; Persistent nociceptive signals transmitted to the CNS cause excessive activation of the glutamate-NMDA receptor system, while concurrent relative insufficiency in descending inhibitory systems collectively exacerbates pain perception and maintenance; Activated microglia and astrocytes in the spinal cord release large amounts of proinflammatory cytokines, forming positive feedback loops with neurons to drive and sustain chronic pain states; Tumors disrupt the bone microenvironment by overactivating osteoclasts through the OPG/RANKL/RANK pathway, causing bone destruction and local microenvironment acidification that directly stimulates nerve endings. These parallel and interacting networks of mechanisms determine the limitations of single-target drugs and underscore the necessity of developing multi-mechanism synergistic intervention strategies. However, cancer pain represents a dynamically evolving process. Existing research predominantly focuses on identifying the role of specific molecules or cells in cancer pain, lacking a framework capable of dynamically guiding multimodal treatment combinations tailored to different stages and subtypes of cancer pain. Consequently, the future of cancer pain management will inevitably rely on integrated strategies that synergistically target multiple pathways and maximize therapeutic windows [245]. Subsequent research must follow a timeline to reveal how the dominant mechanism of pain transitions from primarily “peripheral sensitization” in early-stage tumors to “central sensitization” and “neuroimmune dysregulation” in advanced stages. Only through a profound understanding of these mechanisms can next-generation therapies be developed to directly interrupt the vicious cycle of cancer pain. This will also provide clearer modern scientific evidence and more precise combined treatment targets for integrated Chinese and Western medicine therapies.

Although integrated Chinese and Western medicine therapies demonstrate significant potential in synergistic mechanisms and clinical efficacy enhancement with reduced toxicity, the ultimate realization of therapeutic effects—whether from Western or traditional Chinese medicines—remains highly dependent on the efficiency of drug delivery within the body. Traditional administration methods (such as oral tablets or injections) often encounter issues like significant pharmacokinetic variability, high systemic exposure, and poor targeting. This leads to inconsistent analgesic effects, increased adverse reactions, and reduced patient compliance. To overcome these bottlenecks, advanced DDS have emerged, propelling cancer pain management from the traditional “drug selection” model toward a modern paradigm of “precision delivery.”

Oral controlled-release and sustained-release systems achieve steady, sustained drug release within the gastrointestinal tract through technological optimization, effectively avoiding the “peak-trough” phenomenon in blood drug concentrations. This not only reduces dosing frequency and improves patient compliance but, more crucially, provides stable background analgesia, laying the foundation for managing chronic cancer pain. However, such systems are unsuitable for advanced cancer pain patients with dysphagia. They require time to reach steady-state concentrations in the body. Crushing, chewing, or co-administration with alcohol poses risks of dose bursts, increasing adverse reaction rates. Individual variations in gastrointestinal function and pathological states may also affect drug absorption, leading to inconsistent efficacy or adverse reactions. To overcome these limitations, the development of “rapid-release + sustained-release” combination formulations offers the dual advantages of rapid onset and prolonged analgesia. For patients with swallowing difficulties, the microencapsulation system allows capsules to be opened, enabling patients to swallow intact sustained-release microparticles with soft food. Orally disintegrating tablets provide a convenient alternative that requires no water. Both approaches offer new perspectives for cancer pain management. In the realm of safety, integrating abuse-deterrent technologies is a key direction. The use of physical barriers, added antagonists, or aversive agents can effectively curb the risks of drug abuse and misuse.

Mucosal delivery systems and transdermal delivery systems represent significant advances in non-invasive, rapid-acting, or sustained-release drug administration. Mucosal delivery utilizes the rich vascular and lymphatic networks of oral and nasal mucosa to bypass hepatic first-pass metabolism, enabling rapid drug absorption. This provides breakthrough solutions for cancer pain with onset times measured in minutes. However, current research on the mechanisms of mucosal drug delivery remains insufficient, and numerous technical challenges persist. These include the mucus layer on mucosal surfaces potentially hindering drug penetration, proteases or cytochromes present in mucosa potentially degrading drugs, drugs being readily cleared, certain mucosal sites being prone to triggering immune responses, and mucosal delivery potentially being limited by local irritation or tolerance issues affecting long-term use [246]. Future applications could integrate functional nanocarriers from MDDS into existing formulations [247], leveraging their advantages in targeted positioning and prolonged drug release. Another approach involves loading phospholipid surfactants and octadecylamine into solid lipid NPs, enabling targeted drug delivery to tissues expressing phosphatase. This facilitates penetration through the mucus layer while maintaining close proximity to the absorption membrane, thereby achieving targeted, site-specific mucosal drug delivery [248]. With advances in modern pharmaceutical technology and the ongoing refinement of mucosal delivery theory, more rational and effective mucosal delivery systems are expected to achieve clinical translation, offering additional treatment options for cancer pain patients.

Transdermal patches deliver sustained and stable drug penetration through the skin’s stratum corneum, providing continuous analgesia for up to several days. They are particularly suitable for patients with swallowing difficulties or gastrointestinal dysfunction. While this system significantly expands clinical administration pathways to meet diverse therapeutic needs, transdermal drug absorption is susceptible to skin barrier integrity and environmental factors [249]. Additionally, skin irritation or allergic reactions may occur at the application site. From a safety perspective, localized skin reactions remain a significant factor limiting long-term use. Regarding scalability, challenges persist in the mass production and quality control of patches, while variations in skin permeability among individuals further restrict their standardized application. Advancements in skin permeation strategies now enable transdermal delivery of diverse anticancer therapies—from lipophilic small molecules to hydrophilic biomolecules—promising to overcome limitations of existing transdermal technologies and enhance cancer pain relief efficacy [250]. Beyond this, the advent of MN technology offers an innovative solution for cancer pain management. MN arrays, composed of hundreds of micrometer-scale MNs, physically penetrate the skin’s outermost stratum corneum layer in an instant while stopping just above the dermis, avoiding contact with nerve endings. This enables painless, bloodless, and minimally invasive drug delivery. This characteristic offers significant clinical advantages for cancer pain patients requiring long-term, frequent dosing who may be debilitated or cachectic. It not only enhances transdermal absorption rates and reduces pain perception but also enables convenient self-administration, providing an ideal platform for precise analgesia with high local concentrations and low systemic exposure. Despite its promising prospects, translating MN technology from the laboratory to large-scale clinical application in cancer pain management faces several challenges. For instance, the drug loading capacity per MN is extremely low. For potent opioids with high daily requirements, this may necessitate an exceptionally large patch area. Additionally, manufacturing processes demand high precision and stability to ensure the mechanical strength of needle tips for successful skin penetration while preserving drug bioactivity during production and storage. Finally, long-term safety requires thorough evaluation, including potential risks of skin irritation and possible local immune responses. To address these issues, research should develop novel materials and structures with high drug-loading capacity or construct “NP@MN” composite delivery systems to overcome loading limitations. Production processes must be optimized to ensure mechanical strength and drug stability, while prioritizing materials with excellent biocompatibility and degradability to enhance long-term safety.

For refractory cancer pain unresponsive to conventional treatments, intrathecal targeted drug delivery represents a significant breakthrough. By delivering microdoses of opioids directly into the subarachnoid space via an implantable pump, the medication acts directly on opioid receptors in the dorsal horn of the spinal cord. The required dosage is only one-three-hundredth of an oral dose. This “central-targeted” administration achieves potent analgesia while minimizing systemic side effects, representing one of the highest levels of intervention in cancer pain management. Currently, this technology is primarily applied to patients with moderate-to-severe refractory cancer pain [251]. Although more effective than standard care, its higher cost [252] limits its adoption in primary healthcare settings. Additionally, intrathecal administration is an invasive procedure that may increase infection risk, requires strict technical conditions, and has associated adverse reaction reports [253]. Therefore, clinicians require enhanced training to reduce surgical infection risks and should promptly update infusion techniques to optimize efficacy and lower adverse event rates. Concurrently, increased public funding is urgently needed to expand its application in clinical treatment, thereby benefiting more patients. Beyond this, the application of single-use intrathecal targeted drugs has progressively expanded, now covering diverse targets from cell membranes to nuclei and from neurons to immune glial cells. The intrathecal administration of naturally occurring small molecules offers novel delivery approaches for integrated Chinese and Western medicine pain management. However, translating these cutting-edge targets from basic research into routine clinical practice remains challenging. Long-term drug safety, optimal dosage and administration cycles, and potential off-target effects require systematic evaluation in larger animal models and future human trials. Furthermore, developing agonists/antagonists capable of crossing the blood–brain barrier with higher receptor selectivity is crucial for enhancing the efficacy and safety of such therapies.

Cutting-edge nanotechnology is also paving new paths for next-generation cancer pain treatment. Nanocarrier systems achieve precise drug targeting and controlled release by encapsulating analgesics, enabling extended circulation times and passive or active targeting to sites of inflammation or neuropathy, thereby enhancing efficacy while reducing toxicity [254]. The advancement of bionic nanotechnology also provides insights and momentum for the modernization of TCM, offering new opportunities for cancer pain management [241]. However, nanocarriers themselves may raise new safety concerns, such as immunogenicity, organ accumulation toxicity, and unpredictable biodegradability. Their complex manufacturing processes and inconsistent quality control standards also hinder large-scale clinical application. Furthermore, frequent injection administration may reduce patient compliance, necessitating the development of more convenient dosage forms. For instance, designing nanocarriers that specifically respond to TME characteristics to achieve controlled drug release could enhance efficacy while further reducing off-target effects. Second, deepening the integration of TCM and modern medicine at the nanoscale is crucial. Successful examples of co-delivery bionic nanosystems (Q+M/MnOx)@Clip) demonstrate that combining natural TCM active ingredients like quercetin with modern nanotechnology can synergistically regulate multiple targets in the TME and pain signaling pathways. This offers a highly promising pathway for the modernization and internationalization of TCM. Finally, advancing interdisciplinary collaboration to optimize delivery strategies is paramount. This entails exploring non-invasive delivery methods to partially replace injection routes, integrating NDDS with interventional therapies for “targeted clearance,” and conducting large-scale animal studies alongside rigorous clinical translation research. Ultimately, these promising laboratory findings must be transformed into clinical solutions that genuinely benefit cancer pain patients.

## 7. Summary and Outlook

This paper systematically expounds the synergistic and attenuated advantages of the combination of traditional Chinese and Western medicine in the treatment of cancer pain for the first time. By combining TCM compound preparations with the effects of tonifying kidney and filling marrow, promoting blood circulation and removing blood stasis, clearing heat and detoxifying or warming yang and dredging stagnation with opioids, it can achieve multi-mechanism synergistic analgesic effect, reduce the dosage of opioids and the incidence of adverse reactions, and achieve better efficacy than western medicine alone in pain management. Through the summary of the therapeutic mechanism of analgesic drugs, it is found that the analgesic system can play a therapeutic role by intervening from peripheral sensitization, neuronal excitability changes, central sensitization to bone destruction. In addition, this paper summarizes the application of DDS in the treatment of cancer pain for the first time. The steady-state control of back pain can be achieved by oral sustained and controlled release. Mucosal administration can provide rapid rescue for breakthrough pain. Transdermal administration provides patients with non-invasive and long-term drug delivery options. The ultimate minimally invasive intervention for refractory pain can be achieved by intrathecal targeted delivery technology. The emergence of DDS shows the precision and individualization of analgesic therapy. The cutting-edge research of new DDS in cancer pain management such as MNs, one-time intrathecal injection and NDDS is committed to achieving targeted delivery, intelligent release and long-term sustained release, or will break through the limitations of existing therapies.

Based on the above findings, the evolution of cancer pain management reveals the limitations of single therapy and highlights the inevitable trend of integrating multi-mode strategies. The traditional WHO three-step principle and opioid-led model still have bottlenecks in dealing with complex neuropathic pain and long-term drug side effects. Although the combination of traditional Chinese and Western medicine has shown the potential of “increasing efficiency and reducing toxicity”, its application is mostly based on experience, lacking collaborative theoretical guidance and standardized programs based on precise mechanisms. Advanced DDS provide tools for achieving precise treatment, but their design is often insufficiently combined with the deep pathological mechanism of pain and the needs of specific drug combinations. The multi-mechanism approach of the dynamic evolution of cancer pain determines that the future cancer pain management is not only a simple superposition of drugs or technologies, but also a fine assessment of the type of pain in patients, and a clear understanding of its dominant pain mechanism. Based on this mechanism, a synergistic combination of Chinese and Western medications should be selected, paired with the most suitable delivery system capable of precisely and controllably delivering this combination to the target site. Looking ahead, achieving this transformation requires research to focus on key directions: Utilizing multi-omics technologies and artificial intelligence to establish mapping between different clinical stages of cancer pain and their molecular/cellular characteristics, providing objective, quantifiable standards for mechanism-based classification; Employing systems pharmacology and cutting-edge experimental models to elucidate synergistic networks of Chinese and Western medicines within key signaling pathways, thereby providing theoretical foundations for drug combinations; Advancing delivery systems toward intelligent upgrades enabling responsiveness to specific pain microenvironments, achieving on-demand drug release, and integrating with interventional therapies for precision treatment; Conducting prospective integrated clinical studies to validate the superiority of specific drug combinations paired with tailored delivery methods, gradually establishing clinical guidelines.

In summary, deepening our understanding of cancer pain mechanisms is the cornerstone for advancing treatment. The integration of Chinese and Western medicine with advanced drug delivery technologies will propel cancer pain management toward a new era of enhanced efficacy and safety. Through multidisciplinary collaboration and innovative integration, we can progress beyond current symptom control to achieve disease treatment based on pathological mechanisms, ultimately leading to a fundamental improvement in patients’ quality of life.

## Figures and Tables

**Table 2:** Clinical application of combined TCM and Western medicine in the treatment of cancer pain.

Combination Drugs	Control Drugs	Patients	Treatment Method	Observation Indicators	References
Oxycodone SR + Yishen Gukang Formula	Oxycodone SR + Herbal Placebo	50 cases per group, moderate-severe cancer pain	Control: Oxycodone SR + 10% Yishen Qutong Granules 10 g/tid, 7 d; Observation: Add Yishen Gukang Formula, 2 w	Total efficacy 94%, significant efficacy 70% (*p* < 0.01); NRS score ↓ (*p* < 0.05); Breakthrough pain frequency ↓ (*p* < 0.01); Oxycodone SR dosage ↓ (*p* < 0.05); Adverse reactions (dizziness, drowsiness, constipation, nausea/vomiting) ↓ (*p* < 0.05)	[60]
				Total efficacy 94%, significant efficacy 70% (*p* < 0.01); Oxycodone SR dosage ↓ (*p* < 0.05); Adverse reactions (dizziness, drowsiness, constipation, nausea/vomiting) ↓ (*p* < 0.05)	[61]
Oxycodone SR + Yishen Qutong Granules	Oxycodone SR + Herbal Placebo	Control: 74 cases; Observation: 79 cases, moderate-severe cancer pain	Control: Oxycodone SR + 10% Yishen Qutong Granules 10 g/tid, 28 d; Observation: Add Yishen Qutong Granules 10 g/tid, 28 d	NRS score ↓ (*p* < 0.05); Breakthrough pain frequency ↓ (*p* < 0.01); Oxycodone SR dosage ↓	[62]
Oxycodone SR + Yuanhu Zhitong Tablets	Oxycodone SR	50 cases per group, moderate-severe cancer pain	Control: Oxycodone CR, moderate 10 mg, q 12 h, severe 20 mg, q 12 h, titrated per TIME principle, 7 d; Observation: Add Yuanhu Zhitong Tablets 4–6 tablets/tid, 7 d	Total efficacy > 90% (*p* > 0.05); Breakthrough pain frequency ↓ (*p* < 0.05); Oxycodone SR dosage ↓ (*p* < 0.05); Adverse reactions (nausea, vomiting, constipation) ↓ (*p* < 0.05)	[63]
Morphine SR + Xuefu Zhuyu Decoction	Morphine SR	54 cases per group, cancer pain with blood stasis	Control: 10 mg, q 12 h; Observation: Add Xuefu Zhuyu Decoction, 4 w	Total efficacy 88.89% (*p* < 0.05); NRS score ↓ (*p* < 0.05); PGE2, NO ↓ (*p* < 0.05)	[64]
WHO 3-step analgesia + Xuefu Zhuyu Decoction	WHO 3-step analgesia	Control: 113 cases; Observation: 115 cases, cancer pain with blood stasis	Control: Standard analgesia; Observation: Add Xuefu Zhuyu Decoction	Total efficacy 94.8% (*p* < 0.05); NRS score ↓ (*p* < 0.01); Oral morphine equivalent ↓ (*p* < 0.05); RBC, HB, WBC, PLT, ALT, AST, TBil, Urea, Crea (*p* > 0.05)	[65]
Oxycodone SR + Xuefu Zhuyu Decoction	Oxycodone SR	31 cases per group, cancer pain with Qi deficiency & blood stasis	Control: 10 mg/bid; Observation: Add Xuefu Zhuyu Decoction, 30 d	NRS score ↓ (*p* < 0.05); Adverse reactions (dizziness, nausea, vomiting, constipation, anorexia) ↓ (*p* < 0.05)	[66]
Oxycodone SR + Gutong Plaster		30 cases per group, bone metastasis pain (cold coagulation)	Control: 10 mg/bid; Observation: Add Gutong Plaster topical, once daily	Significant efficacy 93.3% (*p* < 0.05); NRS score ↓ (*p* < 0.05); Breakthrough pain frequency (*p* > 0.05); Oxycodone dosage (*p* > 0.05)	[67]
Zoledronic Acid + Yanghe Decoction with Modifications	Zoledronic Acid	30 cases per group, breast cancer bone metastasis (cold coagulation)	Control: 4 mg IV, q 4 w, total 4 courses; Observation: Add Yanghe Decoction with Modifications, 16 w	Total efficacy 73.4% (*p* < 0.05); NRS score ↓ (*p* < 0.05 or *p* < 0.01); Serum calcium, ALP, NTX ↓ (*p* < 0.01); Adverse reactions (myelosuppression, fatigue, fever) (*p* > 0.05)	[68]
Zoledronic Acid + Yanghe Decoction		42 cases per group, lung cancer bone metastasis pain	Control: 4 mg IV, q 4 w, total 4 courses; Observation: Add Yanghe Decoction, 4 w	Total efficacy 78.5% (*p* < 0.05); Serum NTX, ALP ↓; Adverse reactions (fatigue, myelosuppression, hypokalemia) (*p* > 0.05)	[69]
Oxycodone SR + Peony and Licorice Decoction	Oxycodone SR	50 cases per group, moderate-severe cancer pain	Control: 10 mg, q 12 h; Observation: Add Peony and Licorice Decoction, 14 d	Total efficacy 86.00% (*p* < 0.05); NRS score ↓ (*p* < 0.05); Breakthrough pain frequency ↓ (*p* < 0.05); Adverse reactions (vomiting, constipation, low fever) ↓ (*p* < 0.05)	[70]
		45 cases per group, ovarian cancer pain	Control: 20 mg/bid; Observation: Add Peony and Licorice Decoction, 4 w	Average dosage ↓, onset time ↓, duration ↑ (*p* < 0.05); NRS score ↓ (*p* < 0.05); 5-HT, PGE2, Substance *p* ↓ (*p* < 0.05); TNF-α, IL-1β, IL-6 ↓ (*p* < 0.05); Adverse reactions (nausea, vomiting, dizziness, constipation) (*p* > 0.05)	[71]
Oxycodone SR + Xihuang Pills		Observation: 80 cases; Control: 78 cases, moderate-severe cancer pain	Control: 10 mg, q 12 h; Observation: Add Xihuang Pills 3 g/bid	Total efficacy 95.00% (*p* < 0.05); CD3+, CD4+, CD4+/CD8+ ↑ (*p* < 0.05); Adverse reactions (constipation, nausea/vomiting, dysuria, dizziness, drowsiness) ↓ (*p* < 0.05)	[72]
		50 cases per group, lung cancer pain		Total efficacy 96.00% (*p* < 0.05); NRS score ↓ (*p* < 0.05); Adverse reactions (dizziness/drowsiness, constipation, nausea/vomiting, dysuria, anorexia) (*p* > 0.05)	[73]
Oxycodone SR + Huachansu capsules		39 cases per group, cancer pain	Control: 10 mg/bid, 2 w; Observation: Add Huachansu Capsules 2 pills/tid, 30 d	Total efficacy 89.74% (*p* < 0.05); Adverse reactions (nausea, vomiting, respiratory depression, constipation) ↓ (*p* < 0.05)	[74]
		50 cases per group, moderate-severe cancer pain	Control: 10–20 mg, q 12 h; Observation: Add Huachansu Capsules 500 mg/tid, 2 w	Total efficacy 88.00% (*p* < 0.05); NRS score ↓ (*p* < 0.05); Adverse reactions (constipation, nausea/vomiting, hiccups, dysuria) ↓ (*p* < 0.05)	[75]
Morphine SR + Huachansu capsules	Morphine SR	20 cases per group, malignant tumors	Control: 10 mg, q 12 h; Observation: Add Huachansu Capsules 500 mg/tid, 30 d	Total efficacy 80.00% (*p* < 0.05); NRS score ↓ (*p* < 0.01); Analgesic onset time ↓ (*p* < 0.01); Duration ↑ (*p* < 0.01); Morphine SR dosage ↓ (*p* < 0.01); Adverse reactions (constipation, nausea, vomiting, dizziness, anorexia, dysuria) (*p* > 0.05)	[76]
Fentanyl Transdermal + Huachansu Capsules	Fentanyl Transdermal	Observation: 147 cases; Control: 151 cases, bone metastasis pain	Control: 1 patch, q 72 h; Observation: Add Huachansu Capsules 500 mg/tid, 28 d	Total efficacy 72.10% (*p* < 0.001); Adverse reactions (GI reactions, constipation, dizziness, drowsiness) (*p* > 0.05)	[77]
Zoledronic Acid + Huachansu Capsules	Zoledronic Acid	32 cases per group, prostate cancer bone metastasis	Control: 4 mg IV, q 4 w; Observation: Add Huachansu Capsules 500 mg/tid	Total efficacy 81.25% (*p* < 0.05); Bone density ↓; Serum calcium, phosphorus ↑ (*p* < 0.05)	[78]
Morphine SR + Compound Kushen Injection	Morphine SR	Observation: 43 cases; Control: 30 cases, malignant tumors	Control: 1 dose, q 12 h; Observation: Add Compound Kushen Injection 20 mL/d IV	Total efficacy 62.79% (*p* < 0.05); NRS score ↓ (*p* < 0.01); Adverse reactions (nausea, vomiting, constipation, drowsiness) ↓ (*p* < 0.05)	[79]
Pregabalin + Oxycodone SR + Compound Kushen Injection	A: Oxycodone SR; B: + Pregabalin	22 cases per group, cancer pain	A: 30 mg, q 12 h; B: Add Pregabalin 75 mg, q 12 h; C: Add Compound Kushen Injection 20 ML, qd IV, 4 w	Total efficacy 90.30% (*p* > 0.05); Adverse reactions (dizziness, liver dysfunction, GI dysfunction) ↓	[80]
Morphine SR + Compound Kushen Injection	Morphine SR	Observation: 60 cases; Control: 61 cases, moderate-severe cancer pain	Control: 20 mg, q 12 h; Observation: Add Compound Kushen Injection 20 ML, qd IV, 4 w	Total efficacy 95.00% (*p* < 0.05); NRS score ↓ (*p* < 0.01); Adverse reactions (nausea/vomiting, respiratory depression, dizziness, drowsiness, constipation) ↓ (*p* < 0.05)	[81]
Fentanyl Transdermal + Compound Kushen Injection	Fentanyl Transdermal	50 cases per group, moderate-severe pain	Control: 2.5 mg, q 2 d; Observation: Add Compound Kushen Injection 20 mL, qd IV, 10 d	Total efficacy 84.00% (*p* < 0.01); NRS score ↓ (*p* < 0.01); IL-6, IL-8, CRP ↓ (*p* < 0.01); GI reaction incidence ↓ (*p* < 0.05)	[82]
Morphine SR + Bulleyaconitine A Tablets	Morphine SR	20 cases per group, moderate-severe cancer pain	Control: q 2 d; Observation: Add Bulleyaconitine A Tablets 0.4 mg, q 2 d	Total efficacy 95.00%; Morphine SR dosage ↓ (*p* < 0.05); PGE2, TNF-α ↓ (*p* < 0.05); Adverse reactions (nausea/vomiting, constipation, respiratory depression, drowsiness/fatigue) ↓ (*p* < 0.05)	[83]

↓: reduce; ↑: rise; SR: Sustained Release; tid: three times a day; q 12 h: every 12 h; NRS: Numerical Rating Scale; *p*: *p*-value; GI: gastrointestinal; IV: intravenous; q 4 w: every 4 weeks; q 2 d: every 2 days; CD3+: CD3 positive T cells; CD4+: CD4 positive T cells; CD8+: CD8 positive T cells; CD4+/CD8+: CD4 to CD8 ratio; 5-HT: serotonin; PGE2: prostaglandin E2; TNF-α: tumor necrosis factor-alpha; IL-1β: interleukin-1 beta; IL-6: interleukin-6; IL-8: interleukin-8; CRP: C-reactive protein; ALP: alkaline phosphatase; NTX: N-telopeptide; RBC: red blood cell; HB: hemoglobin; WBC: white blood cell; PLT: platelet; ALT: alanine aminotransferase; AST: aspartate aminotransferase; bid: twice a day; qd: once a day; d: day; w: week; h: hour.

**Table 3:** Therapeutic Mechanisms of Selected Drugs.

Drug Category	Representative Drugs	Core Mechanism of Action	Main Indications for Cancer Pain	References
NSAIDs	Ibuprofen, Indomethacin	Inhibits COX-1/COX-2, reducing prostaglandins and other inflammatory mediators; alleviates inflammation and pain sensitization	Inflammatory Cancer Pain	[87,88]
COX-2 Selective Inhibitor	Celecoxib	Specifically inhibits COX-2, reducing prostaglandin synthesis at inflammatory sites, thereby decreasing inflammation and pain	Inflammatory Cancer Pain	[89]
Opioid Analgesics	Morphine, Fentanyl	Activates μ, κ, δ opioid receptors, inhibiting release of nociceptive neurotransmitters; enhances descending pain inhibitory pathways; modulates microglia to reduce pro-inflammatory factors	Various Cancer Pains	[90,91]
Analgesic and antipyretic agent	Acetaminophen	Inhibits peripheral and central COX, reducing prostaglandin synthesis and weakening pain signal transmission	Mild-Moderate Inflammatory Cancer Pain, Central Cancer Pain	[87,92]
Glucocorticoids	Dexamethasone, Prednisone	Inhibits synthesis of inflammatory mediators; reduces tumor compression; alleviates inflammatory response	Compression Pain, Inflammatory Cancer Pain, Bone Metastasis Pain	[93]
NMDA receptor antagonist	Ketamine	Blocks NMDA receptors, reducing neuronal excitability; modulates neurotransmitters; inhibits neuroinflammation	Refractory Neuropathic Cancer Pain, Opioid-Induced Hyperalgesia, Opioid-Tolerant Pain	[15,94]
TCA	Amitriptyline	Inhibits 5-HT/NE reuptake, enhancing descending pain inhibition; reduces inflammatory factors	Inflammatory Cancer Pain, Neuropathic Cancer Pain	[95]
Antidepressants (SSRIs/SNRIs)	Fluoxetine (SSRI), Venlafaxine (SNRI)	Inhibits 5-HT/NE reuptake, boosting analgesic signaling; at high concentrations, blocks sodium channels	Neuropathic Cancer Pain	[96]
α-2 Adrenergic Agonists	Dexmedetomidine, Clonidine	Enhances descending pain inhibition; promotes release of endogenous opioids, blocking pain signals	Neuropathic Cancer Pain, Visceral Pain	[97]
Anticonvulsants	Phenytoin, Lamotrigine, Carbamazepine, Oxcarbazepine	Inhibits voltage-gated sodium channels, stabilizing hyperexcitable neuronal membranes; some drugs modulate calcium channels.	Neuropathic Cancer Pain	[98,99]
Local Anesthetic	Lidocaine	Blocks voltage-gated sodium channels, inhibiting pain signal conduction	Localized Neuropathic Cancer Pain	[100]
Bisphosphonates	Zoledronic Acid, Pamidronate	Inhibits osteoclast activity, reducing bone resorption; provides anti-inflammatory and analgesic effects	Bone Metastasis Pain	[101]
TCM Formulations	Xihuang Pill	Downregulates COX-2 expression, reducing prostaglandin synthesis and inflammation	Inflammatory Cancer Pain	[102]
	Huachansu	Inhibits microglial/astrocyte activation, reducing inflammation; modulates calcium channels; inhibits osteoclasts	Inflammatory, Neuropathic, Bone Metastasis Pain	[103,104]
	Peony and Licorice Decoction	Downregulates TRPV1, reduces inflammatory factors; inhibits osteoclast activation	Inflammatory, Bone Metastasis Pain	[105,106]
	Compound Kushen Injection	Reduces Nav1.7 channel expression, inhibits microglial activation, repairs spinal barrier	Cancer-induced Bone Pain	[107]
	Bulleyaconitine A Tablets	Activates descending pain inhibition; induces analgesia via κ-opioid receptors; inhibits osteoclasts	Moderate-Severe Cancer Pain	[15,108,109,110]
	Yishen Gukang Formula	Inhibits osteoclast activation; modulates spinal inflammatory signaling (p38 MAPK)	Bone Metastasis Pain	[111]

NSAIDs: Non-Steroidal Anti-Inflammatory Drugs; COX: Cyclooxygenase; COX-1: Cyclooxygenase-1; COX-2: Cyclooxygenase-2; 5-HT: 5-Hydroxytryptamine; NE: Norepinephrine; TCA: Tricyclic Antidepressant; SSRIs: Selective Serotonin Reuptake Inhibitors; SNRIs: Serotonin and Norepinephrine Reuptake Inhibitors; NMDA: N-Methyl-D-Aspartate; α-2: Alpha-2 Adrenergic; TRPV1: Transient Receptor Potential Vanilloid 1; Nav1.7: Voltage-Gated Sodium Channel 1.7; p38 MAPK: p38 Mitogen-Activated Protein Kinase; TCM: Traditional Chinese Medicine.

**Table 4:** Cancer Pain Management with marketed DDS.

Drug Category	Representative Drug	Delivery Systems	First Approved (Year, Country)	Advantages of Delivery System
NSAIDs	Diclofenac sodium	Controlled-Release Tablet	1996 (US)	Steady blood concentration, prolonged duration of action, improved gastrointestinal safety and patient compliance
		Sustained-Release Tablet	1988 (US)	Extended therapeutic effect, reduced dosing frequency, stable blood concentration
		Transdermal patch	2005 (China)	Avoids first-pass metabolism, continuous release, reduces gastrointestinal irritation
Weak opioids	Buprenorphine	Sublingual Tablet	2002 (US)	Rapid absorption, avoids first-pass effect, suitable for acute pain management
		Transdermal patch	2006 (Germany)	Long-acting release (7 days), improves compliance, reduces abuse potential
	Tramadol	Sustained-Release Tablet	1977 (Germany)	Once-daily dosing, provides steady blood concentration, reduces peak-trough fluctuation and dosing frequency
Strong opioids	Fentanyl	Transdermal patch	1990 (US)	72-h continuous analgesia, suitable for patients unable to take oral medication
		Oral Film	2006 (US)	Rapid onset (typically within 15–30 min), for breakthrough pain
		Sublingual Tablet	1997 (US)	Bypassing liver metabolism, highly bioavailable
		Sublingual spray	2012 (US)	Dual-route absorption via buccal and sublingual mucosa, prolonged analgesia, onset in 15–30 min
		Nasal spray	2009 (Europe)	Bypasses first-pass metabolism, rapid onset (5–15 min), precise dosing
	Morphine	Controlled-Release Tablet	2000 (US)	12 h continuous release
		Sustained-Release Tablet	1984 (US)	Once or twice-daily dosing, provides steady and prolonged blood concentration for effective background pain control, reduces dosing frequency
		Intrathecal Pump	1982 (US)	Direct action on spinal cord receptors, dose is only 1/300 of the oral dose, significantly reduces systemic side effects
	Oxycodone	Sustained-Release Tablet	1995 (US)	12-h sustained release, oral bioavailability 60%~87%
Local anesthetic	Lidocaine	Transdermal patch	1999 (US)	High local concentration, low systemic exposure, continuous analgesia (12 h)
α-2 adrenergic agonists	Clonidine	Transdermal patch	1984 (US)	Once-weekly dosing, stable blood pressure/analgesia, avoids sedative effects associated with oral administration

**Table 5:** Nano-Drug Delivery Systems for Cancer Pain Management.

Drug	Delivery Systems	Administration Route	Core Materials and Characterization	Delivery Advantag	Clinical Advantages	References
Ropivacaine	LNPs	IP	Lecithin 66%, Cholesterol 30%, DSPE-PEG2000-RGD 4%, Ropivacaine 30 mg/mL; Size 127.3 nm, Zeta −27.8 mV	24 h release 71.4%	Inhibited mTORC1/JAK2/STAT3 pathway, VEGF-A ↓	[227]
Fentanyl citrate			GMS & Pumpkin seed oil (3:1 *w*/*w*), Poloxamer 407/Tween 80 9 mg/mL; Avg. size 90.7 nm (DLS)/80 nm (TME), PDI 0.2, Zeta −25.05 ± 4.01 mV, EE 82%	72 h SR	50% dosage reduction, low side effects	[228]
Morphine Sulfate	PNPs		Chitosan, Morphine salt, Soybean oil, Span 80, TPP; DL 15.1%, EE 57.0%	72 h cumulative release 99.8% (pH 7.4)	Pain threshold ↑ (*p* < 0.05), 5-HT ↑ (*p* < 0.05), NE ↓ (*p* < 0.05)	[229]
AZ3451		IT	PAMAM-G3-Chol cationic NPs; Size 200 nm, PDI < 0.2, DL 20%	High drug loading capacity (>40%); high efficiency (>99%) 24 h SR	The pain threshold increased to 102% of the baseline level at 6 h and remained elevated for 24 h	[230]
catHEC·FA@SPIO	MNPs	Transdermal	Size 10 nm	72 h cumulative release 82.4 ± 3.1% (pH 5.5)	NRS score in EG (2.35 ± 4.47) (*p* < 0.05)	[231]
HA-AS_2_S_3_	Size-Tunable NPs	IT	HA/As^3+^ molar ratio 1:50; Size 97 nm, PDI < 0.2	Targeted delivery of tumor cells	Good biosafety, eliminates local cancer pain	[232]
Mg/Al-LDH	Co-delivery NPs	IV	Mg/Al LDH, NGF antagonist AZ23, ALD modification; Size 160 nm	48 h release 92% AZ23	Blocked cancer-nerve crosstalk	[233]
(Q+M/MnOx)@Clip	Co-delivery NPs		Soybean lecithin, Cholesterol, Q & M/MnOx (36.1:9.5:4.5:4.5:4.5); Size 270 nm	Homologous targeting	Inhibited HIF-1α/VEGF-A pathway, suppressed VEGF, synergistic therapy & analgesia	[234]
PTX-HP	Carrier-free NPs	IT	PTX:HP = 4:1; High DLE > 60%, Size ~95 nm, PDI < 0.2	SR of PTX in vitro for 3 weeks, long-term retention	Relieve localized cancer pain	[235]

↓: reduce; ↑: rise; LNPs: Lipid Nanoparticles; IP: Intraperitoneal Injection; mTORC1: Mechanistic Target of Rapamycin Complex 1; JAK2: Janus Kinase 2; STAT3: Signal Transducer and Activator of Transcription 3; VEGF-A: Vascular Endothelial Growth Factor A; GMS: Glycerol Monostearate; DLS: Dynamic Light Scattering; TEM: Transmission Electron Microscopy; PDI: Polydispersity Index; EE: Encapsulation Efficiency; SR: Sustained Release; PNPs: Polymer Nanoparticles; TPP: Tripolyphosphate; DL: Drug Loading; 5-HT: 5-Hydroxytryptamine (Serotonin); NE: Norepinephrine; PAMAM-G3: Poly (amidoamine) Dendrimer Generation 3; Chol: Cholesterol; NPs: Nanoparticles; IT: Intratumoral Injection; TME: Tumor Microenvironment; MNPs: Magnetic Nanoparticles; NRS: Numerical Rating Scale; EG: Experimental Group; HA: Hyaluronic Acid; LDH: Layered Double Hydroxide; NGF: Nerve Growth Factor; ALD: Alendronate; IV: Intravenous Injection; HIF-1α: Hypoxia-Inducible Factor 1-alpha; VEGF: Vascular Endothelial Growth Factor; PTX: Paclitaxel; DLE: Drug Loading Efficiency.

## Data Availability

No new data were created or analyzed in this study.

## Abbreviations

WHO	World Health Organization
DDS	Drug Delivery Systems
MDDS	Mucosal Drug Delivery System
TDDS	Transdermal Drug Delivery System
ITDD	Intrathecal Targeted Drug Delivery
NDDS	Nanodrug Delivery Systems
IARC	International Agency for Research on Cancer
NSAIDs	nonsteroidal anti-inflammatory drugs
TCAs	Tricyclic Antidepressants
TCM	traditional Chinese medicine
APAP	Acetaminophen
5-HT	5-hydroxytryptophan
PGE2	prostaglandin E2
PSQI	Pittsburgh Sleep Quality Index
TME	tumor microenvironment
CNS	Central Nervous System
PGs	prostaglandins
IL-1β	interleukin-1β
IL-6	interleukin-6
NGF	nerve growth factor
COX-2	cyclooxygenase
TNF-α	tumor necrosis factor-alpha
AA	arachidonic acid
PGI2	prostacyclin
TRPV1	transient receptor potential vanilloid
DRG	dorsal root ganglion
VGSCs	voltage-gated sodium channels
ASICS	acid-sensing ion channels
TRP	transient receptor potential channels
VGCC	voltage-gated calcium channels
WDR	wide dynamic range
RVM	rostral ventromedial area
NE	Noradrenaline
PAG	periaqueductal gray matter
CXCR	C-X chemokine receptors
CCL2/CCR2	chemokine ligand 2/CC receptor 2
TAK1/JNK/c-Jun	transforming growth factor kinase 1/amino-terminal kinase/c-Jun
PTHrP	parathyroid hormone-related protein
FPPS	farnesyl diphosphate synthase
NFATc1	nuclear factor 1
ADME	Absorption, distribution, metabolism, and excretion
FDA	Food and Drug Administration
BTP	Breakthrough Cancer Pain
FBSF	Fentanyl Buccal Soluble Film
CH/Na-MMT	clonidine hydrochloride/montmorillonite sodium
DCF	Diclofenac sodium
PVA	polyvinyl alcohol
PVP	povidone
IDDS	Intrathecal drug delivery system
PCA	patient-controlled analgesia
NMUR2	neurotransmitter U receptor
HDAC	Histone deacetylase
BCP	bone cancer pain
CCL2	C-C ligand 2
GPCR	G protein-coupled receptor
PPAR-γ	peroxisome proliferator-activated receptor γ
BMSCs	bone marrow mesenchymal stem cells
TPP	triphosphate
GMS	Glycerol monostearate
LDH	layered double hydroxide
ALD	alendronic acid
NPs	nanoparticles
PTX	Paclitaxel
HP	Hematoporphyrin
HA	hyaluronic acid

## References

[1] Liu D., Weng J.-S., Ke X., Wu X.-Y., Huang S.-T. (2023). The Relationship between Cancer-Related Fatigue, Quality of Life and Pain among Cancer Patients. Int. J. Nurs. Sci..

[2] van den Beuken-van Everdingen M.H.J., de Rijke J.M., Kessels A.G., Schouten H.C., van Kleef M., Patijn J. (2007). Prevalence of Pain in Patients with Cancer: A Systematic Review of the Past 40 Years. Ann. Oncol..

[3] Gebhart G.F., Bielefeldt K. (2016). Physiology of Visceral Pain. Compr. Physiol..

[4] Wang G. (2017). Mechanisms of cancer pain. Chin. J. Cancer Prev. Control..

[5] Joshy G., Khalatbari-Soltani S., Soga K., Butow P., Laidsaar-Powell R., Koczwara B., Rankin N.M., Brown S., Weber M., Mazariego C. (2023). Pain and Its Interference with Daily Living in Relation to Cancer: A Comparative Population-Based Study of 16,053 Cancer Survivors and 106,345 People without Cancer. BMC Cancer.

[6] World Health Organization (2018). WHO Guidelines for the Pharmacological and Radiotherapeutic Management of Cancer Pain in Adults and Adolescents; WHO Guidelines Approved by the Guidelines Review Committee.

[7] Shkodra M., Caraceni A. (2022). Treatment of Neuropathic Pain Directly Due to Cancer: An Update. Cancers.

[8] Fallon M., Giusti R., Aielli F., Hoskin P., Rolke R., Sharma M., Ripamonti C.I., ESMO Guidelines Committee (2018). Management of Cancer Pain in Adult Patients: ESMO Clinical Practice Guidelines. Ann. Oncol..

[9] Zhang W., Xu S., Wang C. (2023). Study on the medication rules of Traditional Chinese Medicine for cancer pain treatment. Chin. J. Emerg. Tradit. Chin. Med..

[10] Chi L.T., Zheng Y. (2025). Research progress on integrated Chinese and Western Medicine for cancer pain treatment. Guangming J. Chin. Med..

[11] Zhang W., Wu C., Liu J. (2023). Schwann Cells as a Target Cell for the Treatment of Cancer Pain. Glia.

[12] Ramos-Inza S., Ruberte A.C., Sanmartín C., Sharma A.K., Plano D. (2021). NSAIDs: Old Acquaintance in the Pipeline for Cancer Treatment and Prevention─structural Modulation, Mechanisms of Action, and Bright Future. J. Med. Chem..

[13] Paulsen Ø., Aass N., Kaasa S., Dale O. (2013). Do Corticosteroids Provide Analgesic Effects in Cancer Patients? A Systematic Literature Review. J. Pain Symptom Manag..

[14] Wang L., Zheng G., Lu S., Ren J., Li C. (2025). Research Advances in Traditional Chinese Medicine for Cancer Pain Management. Chin. J. Exp. Tradit. Med. Formulae.

[15] Vardy J., Agar M. (2014). Nonopioid Drugs in the Treatment of Cancer Pain. J. Clin. Oncol..

[16] Tanaka R., Vecchio S.L., Aliotta G.E., Arendt-Nielsen L. (2025). The Role of Peripheral N-methyl-D-asparate (NMDA) Receptors in Itch and Pain: A Narrative Review. Eur. J. Pain.

[17] Zuo Q., Xu D., Yue S., Fu R., Tang Y. (2024). Chemical Composition, Pharmacological Effects and Clinical Applications of Cinobufacini. Chin. J. Integr. Med..

[18] Hanna M., Thipphawong J. (2008). A Randomized, Double-Blind Comparison of OROS(R) Hydromorphone and Controlled-Release Morphine for the Control of Chronic Cancer Pain. BMC Palliat. Care.

[19] Mercadante S., Radbruch L., Popper L., Korsholm L., Davies A. (2009). 685 Efficacy of Intranasal Fentanyl Spray (Infs) versus Oral Transmucosal Fentanyl Citrate (Otfc) for Breakthrough Cancer Pain: Open-Label Crossover Trial. Eur. J. Pain.

[20] Peña-Juárez M.C., Guadarrama-Escobar O.R., Escobar-Chávez J.J. (2022). Transdermal Delivery Systems for Biomolecules. J. Pharm. Innov..

[21] Bhatia G., Lau M.E., Gulur P. (2013). Intrathecal Drug Delivery (ITDD) Systems for Cancer Pain. F1000Research.

[22] Mercadante S. (1998). Predictive Factors and Opioid Responsiveness in Cancer Pain1. Eur. J. Cancer.

[23] Wiffen P.J., Derry S., Moore R.A., McNicol E.D., Bell R.F., Carr D.B., McIntyre M., Wee B. (2017). Oral Paracetamol (Acetaminophen) for Cancer Pain. Cochrane Database Syst. Rev..

[24] Brune K., Patrignani P. (2015). New Insights into the Use of Currently Available Non-Steroidal Anti-Inflammatory Drugs. J. Pain Res..

[25] Strawson J. (2018). Nonsteroidal Anti-Inflammatory Drugs and Cancer Pain. Curr. Opin. Support. Palliat. Care.

[26] Adler J., Mallick-Searle T., Garofoli M., Zimmerman A. (2024). Frontline Perspectives on Buprenorphine for the Management of Chronic Pain. J. Multidiscip. Healthc..

[27] Cox A.R., Ferner R. (2021). Tramadol: Repeated Prescriptions and Repeated Warnings. BMJ Evid.-Based Med..

[28] National Health Commission of The People’s Republic of China (2018). Standard diagnosis and treatment of cancer pain (version 2018). J. Clin. Oncol..

[29] Paul A.K., Smith C.M., Rahmatullah M., Nissapatorn V., Wilairatana P., Spetea M., Gueven N., Dietis N. (2021). Opioid Analgesia and Opioid-Induced Adverse Effects: A Review. Pharmaceuticals.

[30] Zhang X., Dang D.-S., Sun X., Kang Y. (2025). The Role of Combined Adjuvant Therapeutic Drugs in Modulating Opioid Dosage and Pain Status in Cancer Patients: A Systematic Review and Meta-Analysis. Support. Care Cancer.

[31] Jóźwiak-Bebenista M., Nowak J.Z. (2014). Paracetamol: Mechanism of Action, Applications and Safety Concern. Acta Pol. Pharm..

[32] Moore A.R., Derry S., Straube S., Ireson-Paine J., Wiffen P.J. (2014). Faster, Higher, Stronger? Evidence for Formulation and Efficacy for Ibuprofen in Acute Pain. Pain.

[33] Brogden R.N., Heel R.C., Pakes G.E., Speight T.M., Avery G.S. (1980). Diclofenac Sodium: A Review of Its Pharmacological Properties and Therapeutic Use in Rheumatic Diseases and Pain of Varying Origin. Drugs.

[34] Clissold S.P. (1986). Aspirin and Related Derivatives of Salicylic Acid. Drugs.

[35] Brogden R.N., Heel R.C., Speight T.M., Avery G.S. (1979). Naproxen up to Date: A Review of Its Pharmacological Properties and Therapeutic Efficacy and Use in Rheumatic Diseases and Pain States. Drugs.

[36] Saxena P., Sharma P.K., Purohit P. (2020). A Journey of Celecoxib from Pain to Cancer. Prostaglandins Other Lipid Mediat..

[37] Fleischmann R., Iqbal I., Slobodin G. (2002). Meloxicam. Expert Opin. Pharmacother..

[38] Katz I.M. (1981). Indomethacin. Ophthalmology.

[39] Motov S., Yasavolian M., Likourezos A., Pushkar I., Hossain R., Drapkin J., Cohen V., Filk N., Smith A., Huang F. (2017). Comparison of Intravenous Ketorolac at Three Single-Dose Regimens for Treating Acute Pain in the Emergency Department: A Randomized Controlled Trial. Ann. Emerg. Med..

[40] Barakat A. (2019). Revisiting Tramadol: A Multi-Modal Agent for Pain Management. CNS Drugs.

[41] Heel R.C., Brogden R.N., Speight T.M., Avery G.S. (1979). Buprenorphine: A Review of Its Pharmacological Properties and Therapeutic Efficacy. Drugs.

[42] Wiffen P.J., Wee B., Moore R.A. (2016). Oral Morphine for Cancer Pain. Cochrane Database Syst. Rev..

[43] Schmidt-Hansen M., Bennett M.I., Arnold S., Bromham N., Hilgart J.S. (2015). Oxycodone for Cancer-Related Pain. Cochrane Database Syst. Rev..

[44] Stanley T.H. (2014). The Fentanyl Story. J. Pain.

[45] Sarhill N., Walsh D., Nelson K.A. (2001). Hydromorphone: Pharmacology and Clinical Applications in Cancer Patients. Support. Care Cancer.

[46] Kreutzwiser D., Tawfic Q.A. (2020). Methadone for Pain Management: A Pharmacotherapeutic Review. CNS Drugs.

[47] Bar Ad V. (2010). Gabapentin for the Treatment of Cancer-Related Pain Syndromes. Rev. Recent Clin. Trials.

[48] Bennett M.I., Laird B., van Litsenburg C., Nimour M. (2013). Pregabalin for the Management of Neuropathic Pain in Adults with Cancer: A Systematic Review of the Literature. Pain Med..

[49] Birkinshaw H., Friedrich C.M., Cole P., Eccleston C., Serfaty M., Stewart G., White S., Moore R.A., Phillippo D., Pincus T. (2023). Antidepressants for Pain Management in Adults with Chronic Pain: A Network Meta-Analysis. Cochrane Database Syst. Rev..

[50] Saarto T., Wiffen P.J. (2005). Antidepressants for Neuropathic Pain. Cochrane Database Syst. Rev..

[51] Lee J.T., Sanderson C.R., Xuan W., Agar M. (2019). Lidocaine for Cancer Pain in Adults: A Systematic Review and Meta-Analysis. J. Palliat. Med..

[52] Mercadante S., Calderone L., Barresi L. (1998). Intrathecal Ropivacaine in Cancer Pain. Reg. Anesth. Pain Med..

[53] Weinfurt K.P., Anstrom K.J., Castel L.D., Schulman K.A., Saad F. (2006). Effect of Zoledronic Acid on Pain Associated with Bone Metastasis in Patients with Prostate Cancer. Ann. Oncol..

[54] Eastman P., Currow D.C., Fazekas B., Brown L., Le B. (2019). Oral Dexamethasone in the Management of Cancer-Related Pain: A Feasibility Study. Palliat. Med..

[55] Tannock I., Gospodarowicz M., Meakin W., Panzarella T., Stewart L., Rider W. (1989). Treatment of Metastatic Prostatic Cancer with Low-Dose Prednisone: Evaluation of Pain and Quality of Life as Pragmatic Indices of Response. J. Clin. Oncol..

[56] Kumar A., Maitra S., Khanna P., Baidya D.K. (2014). Clonidine for Management of Chronic Pain: A Brief Review of the Current Evidences. Saudi J. Anaesth..

[57] Liu H.-J., Gao X.-Z., Liu X.-M., Xia M., Li W.-Y., Jin Y. (2014). Effect of Intrathecal Dexmedetomidine on Spinal Morphine Analgesia in Patients with Refractory Cancer Pain. J. Palliat. Med..

[58] Ghanavatian S., Derian A. (2025). Tizanidine. StatPearls.

[59] Tawfic Q.A. (2013). A Review of the Use of Ketamine in Pain Management. J. Opioid Manag..

[60] Hongli S., Yukun Y., Lei Z., Li F. (2018). Randomized Controlled Double-blind Clinical Study on Yishen Gukang Recipe Combined with Oxycodone Hydrochloride Sustained-release Tablets in the Treatment of Moderate to Severe Cancerous Pain with Kidney Deficiency and Blood Stasis Syndrome. J. Tradit. Chin. Med..

[61] Song L., Yin Y., Wang H., Liu L., Zhou L., Li F. (2018). Effect and Safety of Yishen Gukang Decoction Combined with Oxycontin in Treating Cancer Somatic Pain. Chin. J. Exp. Tradit. Med. Formulae.

[62] Zang Q., Li H., Cui Y., Cui X., Wang L., Cang J., Feng L. (2025). Clinical effect of Yishen Qutong Granules in alleviating bone metastasis cancer pain associated with kidney deficiency and blood stasis and its impact on gut microbiota. China J. Tradit. Chin. Med. Pharm..

[63] Tan D., Zeng X., Bao Q., Chen J. (2020). Clinical observation of Yuanhu Zhitong tablets combined with strong opioids in treatment of patients with moderate to severe pain after lung cancer surgery. J. Reg. Anat. Oper. Surg..

[64] Zang H., Lin Y., Lu F. (2023). Clinical study on modified Xuefu Zhuyu decoction supplemented with morphine hydrochloride sustained-release tablets intreatmen to fliver cancer pain of Yuxue Neizu syndrome. Shaanxi J. Tradit. Chin. Med..

[65] Zheng Q., Zhou T., Xiang S. (2022). Clinical Study of Xuefu Zhuyu Decoction in the Treatment of Pain from Advanced Lung Cancer with Yuxuezuluo Syndrome. Pharmacol. Clin. Chin. Mater. Medica.

[66] Xu Z., Ma W. (2019). Study on the curative effect of Xuefu Zhuyu Decoction combined with routine western medicine on cancer pain with Qi deficiency and blood stasis. J. Community Med..

[67] Hou Z. (2022). A Clinical Exploratory Study on the External Application of Bone Pain Patches for Treating Bone Metastatic Cancer Pain of the Yin Cold Stagnation Pattern. Master’s Thesis.

[68] Lei F., Ling L., Yang L., Xiao Y., Dan M. (2022). Clinical effect of Yanghe decoction combined with zoledronic acid in treatment of breast cancer bone metastasis with Yang deficiency and cold congelation: An analysis of 30 cases. Hunan J. Tradit. Chin. Med..

[69] Chunmei Z., Minli T., Yuxuan G., Qian X., Huifang C., Shuai H., Lihuai W., Yuantao W. (2021). Therapeutic effect of Yanghe decoction combined with zoledronic acid on bone metastasis of lung cancer and changes of serum NTX and ALP levels. Anti-Tumor Pharm..

[70] Feng X., Wang N., Zhou L., Li J. (2023). Clinical efficacy and prognostic impact of Shaoyao Gancao Decoction combined with oxycodone extended-release tablets in the treatment of moderate to severe cancer pain. Clin. J. Chin. Med..

[71] Xu B., Chen H., Wang R. (2025). Clinical observation of Shaoyao Gancao Decoction combined with oxycodone hydrochloride in the treatment of ovarian cancer pain. Chin. J. Emerg. Tradit. Chin. Med..

[72] You H. (2018). Xihuang Pills Combined with Oxycodone Hydrochloride Prolonged—Release Tablets Treatment in Patients with Cancer Pain and Its Clinical Effect on Immune Function and Quality of Life. Chin. Arch. Tradit. Chin. Med..

[73] Yan Q., Yang W., Xiong W. (2021). Evaluation of the efficacy and analysis of the mechanism of action of Xihuangwan combined with oxycodone extended-release tablets in the treatment of cancer pain in lung cancer. Med. Theory Pract..

[74] Yun F. (2023). The efficacy of huachansu capsule combined with oxycodone hydrochloride extended-release tablets in the treatment of cancer pain. Heilongjiang J. Tradit. Chin. Med..

[75] Zhang Y., Zhang X., Wan L. (2021). The efficacy of huachansu capsule combined with oxycodone hydrochloride extended-release tablets in the treatment of cancer pain. Forum Tradit. Chin. Med..

[76] Dong X., Li Y., Zhang Y., Zhao Y., Wu S., Xiao Y. (2018). Efficacy of morphine sulfate sustained-release tablets combined with cinobufotalin capsule in the treatment of cancer pain. Chin. Community Dr..

[77] Yang S., Zhao H., Wang Z., Ge X., Lu Y., Jin H., Bo X., Zhang H., Ma L., Pan Y. (2019). Efficacy of Cinobufacin Capsule Combined with Fentanyl Transdermal Patch in the Treatment of Moderate and Severe Cancer Pain with Bone Metastasis. Chin. Gen. Pract..

[78] Zhang F., Dong Y., Chen F., Niu M., Liu Z., Wang C. (2024). Cinobufotalin Capsule Combined with Zoledronic Acid in the Treatment of Pain Symptoms and Clinical Efficacy in Prostate Cancer Patients with Bone Metastases: A Retrospective Study. Arch. Españoles Urol..

[79] Xiang P., Chen Y., Wang W. (2022). Clinical Effect of Compound Kushen Injection As An Adjunct in Treatment of Severe Cancer Pain. Liaoning J. Chin. Med..

[80] Zhou L., Wang W. (2023). Clinical Observation of Pregabalin Combined with Oxycodone Hydrochloride Sustained-release Tablets and Compound Matrine Injection in the Treatment of Patients with Neuropathic Cancer Pain. Liaoning J. Chin. Med..

[81] Miao J., Wang W. (2023). Clinical Observation of Morphine Hydrochloride Sustained Release Tablets Combined with Compound Kushen Injection in Treatment of Moderate and Severe Cancer Pain. Liaoning J. Chin. Med..

[82] Zhang J., Xiao L., Zhang X. (2025). Therapeutic effect of compound kushen injection combined with fentanyl transdermal patch in the treatment of carcinoma pain. Tianjin Med. J..

[83] Fang Ni J., Wu B., Tang Y. (2014). Clinical study of Bulleyaconitine A assisted to Morphine Sulfate Sustained-release Tablets in the treatment of mid-late cancer pain. J. Chang. Univ. Chin. Med..

[84] He L., Liang M., Lu D., Li F. (2021). Effect of Yishen Gukang Formula on Pain Thresholds and Bone Destruction in Rat Suffering from Bone Metastatic Pain. Liaoning J. Tradit. Chin. Med..

[85] Liang F. (2016). Application of Corydalis and Fentanyl Combination Tablets in Postoperative Pain Management for Elderly Orthopedic Patients. Asia-Pac. Tradit. Med..

[86] Gong Z., Chen T., Deng L., Hu Y., Yu X. (2010). Clinical application progress of cinobufagin to cancer pain relief. Drugs Clin..

[87] Cooper T.E., Heathcote L.C., Anderson B., Grégoire M.-C., Ljungman G., Eccleston C. (2017). Non-Steroidal Anti-Inflammatory Drugs (NSAIDs) for Cancer-Related Pain in Children and Adolescents. Cochrane Database Syst. Rev..

[88] Sisignano M., Geisslinger G. (2023). Rethinking the Use of NSAIDs in Early Acute Pain. Trends Pharmacol. Sci..

[89] Cruz J.V., Rosa J.M.C., Kimani N.M., Giuliatti S., dos Santos C.B.R. (2022). The Role of Celecoxib as a Potential Inhibitor in the Treatment of Inflammatory Diseases—A Review. Curr. Med. Chem..

[90] Fatt M.P., Zhang M., Kupari J., Altınkök M., Yang Y., Hu Y., Svenningsson P., Ernfors P. (2024). Morphine-Responsive Neurons That Regulate Mechanical Antinociception. Science.

[91] Tu H., Chu H., Guan S., Hao F., Xu N., Zhao Z., Liang Y. (2021). The Role of the M1/M2 Microglia in the Process from Cancer Pain to Morphine Tolerance. Tissue Cell.

[92] Spence J.D., Grosser T., FitzGerald G.A. (2022). Acetaminophen, Nonsteroidal Anti-Inflammatory Drugs, and Hypertension. Hypertension.

[93] Brasseur L. (1997). Revue Des Thérapeutiques Pharmacologiques Actuelles de La Douleur. Drugs.

[94] Zhang X., Zhang Y., Du W. (2024). Alleviating Role of Ketamine in Breast Cancer Cell-Induced Osteoclastogenesis and Tumor Bone Metastasis-Induced Bone Cancer Pain through an SRC/EGR1/CST6 Axis. BMC Cancer.

[95] Magni G., Conlon P., Arsie D. (1987). Tricyclic Antidepressants in the Treatment of Cancer Pain: A Review. Pharmacopsychiatry.

[96] Verdu B., Decosterd I., Buclin T., Stiefel F., Berney A. (2008). Antidepressants for the Treatment of Chronic Pain. Drugs.

[97] Kulka P.J. (1996). Alpha 2-Adrenoceptor Agonists for the Treatment of Chronic Pain. Schmerz.

[98] Brito B.E., García M.A., Gouveia Y.M.D., Bolaños P., Devis S., Bernal G., Tortorici-Brito V.A., Baute L., Díaz-Serrano G., Tortorici V. (2021). Concomitant Antihyperalgesic and Antitumor Effects of Gabapentin in a Murine Cancer Pain Model. Int. J. Mol. Sci..

[99] Schaible H.-G., Ebersberger A., Natura G. (2011). Update on Peripheral Mechanisms of Pain: Beyond Prostaglandins and Cytokines. Arthritis Res. Ther..

[100] Luo L., Cheng Y., Wang H., Li L., Niu H., Yang Y., Zhou Q., He J., Xu J. (2025). Lidocaine—A Promising Candidate for the Treatment of Cancer-Induced Bone Pain: A Narrative Review. Adv. Ther..

[101] Tzschentke T.M. (2021). Pharmacology of Bisphosphonates in Pain. Br. J. Pharmacol..

[102] Yang Y., Zeng J., Chen P., Wang M., Yin Z., Li L., Dai Y., Zhao J., Li Y., Wen G. (2022). Anti-tumor Application and Pharmacological Mechanism of Xihuangwan: A Review. Chin. J. Exp. Tradit. Med. Formulae.

[103] Lai G., Wang F., Nie D., Zhou F., An G., Wu Z., Bai Q., Cao J. (2023). Pathogenesis of Bone Metastasis-caused Pain and Its Prevention and Treatment with Traditional Chinese Medicine: A Review. Chin. J. Exp. Tradit. Med. Formulae.

[104] Apryani E., Ali U., Wang Z., Wu H., Mao X., Ahmad K.A., Li X., Wang Y. (2020). The Spinal Microglial IL-10/β-Endorphin Pathway Accounts for Cinobufagin-Induced Mechanical Antiallodynia in Bone Cancer Pain Following Activation of A7-Nicotinic Acetylcholine Receptors. J. Neuroinflamm..

[105] Li Z., Zhang J., Ren X., Liu Q., Yang X. (2018). The Mechanism of Quercetin in Regulating Osteoclast Activation and the PAR2/TRPV1 Signaling Pathway in the Treatment of Bone Cancer Pain. Int. J. Clin. Exp. Pathol..

[106] Sui F., Zhou H.-Y., Meng J., Du X.-L., Sui Y.-P., Zhou Z.-K., Dong C., Wang Z.-J., Wang W.-H., Dai L. (2016). A Chinese Herbal Decoction, Shaoyao-Gancao Tang, Exerts Analgesic Effect by Down-Regulating the TRPV1 Channel in a Rat Model of Arthritic Pain. Am. J. Chin. Med..

[107] Gao L., Li Q.-W., Zhang X.-Y., You R.-L., Qin X.-M., Qin W.-J. (2025). The Therapeutic Mechanisms of Compound Kushen Injection in Relieving Cancer-Induced Bone Pain by Targeting Nav1.7 and Microglial Activation. J. Ethnopharmacol..

[108] Guo Y., Huang W., Liang L., Fan Y., Yuan C., Lei X. (2021). Effects of bulleyaconitine A on osteoclast differentiation and its mechanism. Zhejiang Med. J..

[109] Wei J., Wang Y., Kou S. (2020). Mechanisms of Bulleyaconitine A Relieving Pain in Rats with Neuropathic Pain by Regulating TRPV1 Receptor Function. Hebei Med..

[110] Xie M.-X., Zhu H.-Q., Pang R.-P., Wen B.-T., Liu X.-G. (2018). Mechanisms for Therapeutic Effect of Bulleyaconitine A on Chronic Pain. Mol. Pain.

[111] Shen H. (2019). Mechanism of Action and Drug Development Potential of Yishen Gukang Formula in Inhibiting Bone Cancer Pain. Ph.D. Thesis.

[112] Mantyh P.W., Clohisy D.R., Koltzenburg M., Hunt S.P. (2002). Molecular Mechanisms of Cancer Pain. Nat. Rev. Cancer.

[113] Shrihari T. (2017). Dual Role of Inflammatory Mediators in Cancer. Ecancermedicalscience.

[114] Wang Y., Wang Q., Yang T.W., Yin J.M., Wei F., Liu H., Yang P.X., Li J., Liu N., Zhu Y. (2023). Analysis of Immune and Inflammatory Microenvironment Characteristics of Noncancer End-Stage Liver Disease. J. Interferon Cytokine Res..

[115] Zhang S., Chen W., Zhou J., Liang Q., Zhang Y., Su M., Zhang Z., Qu J. (2025). The Benefits and Safety of Monoclonal Antibodies: Implications for Cancer Immunotherapy. J. Inflamm. Res..

[116] Sen N., Tanwar S., Jain A., Sharma J., Gokhroo R.K., Mehta A., Kalra B. (2019). P6293Assessment of Testosterone/Estradiol Ratio, DHEA-S Level and Correlation with Coronary Inflammatory Markers IL-1 & 6, TNF-1 and hsCRP Predict 5 Years Risk of Cardiovascular Disease in Men. Eur. Heart J..

[117] Sabino M.A.C., Ghilardi J.R., Jongen J.L.M., Keyser C.P., Luger N.M., Mach D.B., Peters C.M., Rogers S.D., Schwei M.J., de Felipe C. (2002). Simultaneous Reduction in Cancer Pain, Bone Destruction, and Tumor Growth by Selective Inhibition of Cyclooxygenase-2. Cancer Res..

[118] Zhang F., Wang Y., Liu Y., Han H., Zhang D., Fan X., Du X., Gamper N., Zhang H. (2019). Transcriptional Regulation of Voltage-Gated Sodium Channels Contributes to GM-CSF-Induced Pain. J. Neurosci..

[119] Schmidt B.L., Hamamoto D.T., Simone D.A., Wilcox G.L. (2010). Mechanism of Cancer Pain. Mol. Interv..

[120] Ebersberger A. (2018). The Analgesic Potential of Cytokine Neutralization with Biologicals. Eur. J. Pharmacol..

[121] Burnstock G. (2000). P2X Receptors in Sensory Neurones. Br. J. Anaesth..

[122] Watson J.J., Allen S.J., Dawbarn D. (2008). Targeting Nerve Growth Factor in Pain: What Is the Therapeutic Potential?. BioDrugs.

[123] Smith T.P., Haymond T., Smith S.N., Sweitzer S.M. (2014). Evidence for the Endothelin System as an Emerging Therapeutic Target for the Treatment of Chronic Pain. J. Pain Res..

[124] Fujita M., Andoh T., Ohashi K., Akira A., Saiki I., Kuraishi Y. (2010). Roles of Kinin B1 and B2 Receptors in Skin Cancer Pain Produced by Orthotopic Melanoma Inoculation in Mice. Eur. J. Pain.

[125] Yang P.-P., Yeh G.-C., Huang E.Y.-K., Law P.-Y., Loh H.H., Tao P.-L. (2015). Effects of Dextromethorphan and Oxycodone on Treatment of Neuropathic Pain in Mice. J. Biomed. Sci..

[126] Okura D., Horishita T., Ueno S., Yanagihara N., Sudo Y., Uezono Y., Minami T., Kawasaki T., Sata T. (2015). Lidocaine Preferentially Inhibits the Function of Purinergic P2X7 Receptors Expressed in Xenopus Oocytes. Anesth. Analg..

[127] Chu X., Hou Z., Mao Y., Cai Y., Jiang P., Zhu S. (2019). Network pharmacology study on the mechanism of shaoyao gancao decoction in treating cancer pain. J. Hainan Med. Univ..

[128] Lai L., Feng X., Liu G., Feng Z. (2022). Exploring Mechanism of Cinobutanin in Prevention and Treatment of Bone Cancer Pain Combined with Network Analysis Method. Liaoning J. Tradit. Chin. Med..

[129] Wang W.-L., Hao Y.-H., Pang X., Tang Y.-L. (2025). Cancer Pain: Molecular Mechanisms and Management. Mol. Biomed..

[130] Bujak J.K., Kosmala D., Szopa I.M., Majchrzak K., Bednarczyk P. (2019). Inflammation, Cancer and Immunity—Implication of TRPV1 Channel. Front. Oncol..

[131] Scherer P.C., Zaccor N.W., Neumann N.M., Vasavda C., Barrow R., Ewald A.J., Rao F., Sumner C.J., Snyder S.H. (2017). TRPV1 Is a Physiological Regulator of μ-Opioid Receptors. Proc. Natl. Acad. Sci. USA.

[132] Haroun R., Wood J.N., Sikandar S. (2023). Mechanisms of Cancer Pain. Front. Pain Res..

[133] Sha Y., Shao S., Yang J., Tang B., Quan Y., Liang Q., Wang Z. (2025). Elucidation of the molecular mechanisms of TRPA1 channel protein in pain modulation and advancements in the development of antagonists for cancer pain management. Chin. J. Oncol. Prev. Treat..

[134] Spekker E., Körtési T., Vécsei L. (2022). TRP Channels: Recent Development in Translational Research and Potential Therapeutic Targets in Migraine. Int. J. Mol. Sci..

[135] Basso L., Aboushousha R., Fan C.Y., Iftinca M., Melo H., Flynn R., Agosti F., Hollenberg M.D., Thompson R., Bourinet E. (2019). TRPV1 Promotes Opioid Analgesia during Inflammation. Sci. Signal..

[136] Lampert A., O’Reilly A.O., Reeh P., Leffler A. (2010). Sodium Channelopathies and Pain. Pflügers Arch.-Eur. J. Physiol..

[137] Yang X., Wei X., Mu Y., Li Q., Liu J. (2020). A Review of the Mechanism of the Central Analgesic Effect of Lidocaine. Medicine.

[138] Schmitt L.-I., Leo M., Kleinschnitz C., Hagenacker T. (2018). Oxaliplatin Modulates the Characteristics of Voltage-Gated Calcium Channels and Action Potentials in Small Dorsal Root Ganglion Neurons of Rats. Mol. Neurobiol..

[139] Ismy J., Emril D.R., Khalilullah S.A., Mauny M.P. (2023). Evaluation of Gabapentin as a Treatment of Breakthrough Cancer Pain Caused by Metastatic Prostate Adenocarcinoma. J. Pain Res..

[140] Fazzari J., Lin H., Murphy C., Ungard R., Singh G. (2015). Inhibitors of Glutamate Release from Breast Cancer Cells; New Targets for Cancer-Induced Bone-Pain. Sci. Rep..

[141] Falk S., Dickenson A.H. (2014). Pain and Nociception: Mechanisms of Cancer-Induced Bone Pain. J. Clin. Oncol..

[142] Liu Q., Yao X., Gao S., Li R., Li B.J., Yang W., Cui R. (2020). Role of 5-HT Receptors in Neuropathic Pain: Potential Therapeutic Implications. Pharmacol. Res..

[143] Liu X., Wang G., Ai G., Xu X., Niu X., Zhang M. (2020). Selective Ablation of Descending Serotonin from the Rostral Ventromedial Medulla Unmasks Its Pro-Nociceptive Role in Chemotherapy-Induced Painful Neuropathy. J. Pain Res..

[144] Kim Y.S., Chu Y., Han L., Li M., Li Z., LaVinka P.C., Sun S., Tang Z., Park K., Caterina M.J. (2014). Central Terminal Sensitization of TRPV1 by Descending Serotonergic Facilitation Modulates Chronic Pain. Neuron.

[145] Pertovaara A. (2006). Noradrenergic Pain Modulation. Prog. Neurobiol..

[146] Wang W. (2019). Investigation and Analysis on the Treatment of Cancer Pain in Beijing and Clinical Study on the Treatment of Mild and Moderate Cancer Pain with Bulleyaconitine A. Ph.D. Thesis.

[147] Wang X., Li L., Wang Y. (2025). Mechanisms of Cancer-Induced Bone Pain. J. Pain Res..

[148] Zheng X., Wu Y., Huang J., Wu A. (2022). Neurophysiological Mechanisms of Cancer-Induced Bone Pain. J. Adv. Res..

[149] Lu Y., Li B., Wang R., Wan Q., Zeng J. (2022). Research progress of chemokines and their receptors in cancer pain. Chin. J. Pathophysiol..

[150] Yoneda T., Hiasa M., Okui T., Hata K. (2023). Cancer-Nerve Interplay in Cancer Progression and Cancer-Induced Bone Pain. J. Bone Miner. Metab..

[151] Jin X., Wang L.-N., Zuo J.-L., Yang J.-P., Liu S. (2014). P2X4 Receptor in the Dorsal Horn Partially Contributes to Brain-Derived Neurotrophic Factor Oversecretion and Toll-like Receptor-4 Receptor Activation Associated with Bone Cancer Pain. J. Neurosci. Res..

[152] Lopez-Ortiz A.O., Eyo U.B. (2024). Astrocytes and Microglia in the Coordination of CNS Development and Homeostasis. J. Neurochem..

[153] Ye J., Yan H., Xia Z. (2018). Oxycodone Ameliorates the Inflammatory Response Induced by Lipopolysaccharide in Primary Microglia. J. Pain Res..

[154] Luo F., Zhang J., Miao Y., Wu D., Shen H., Lu M. (2024). Paeoniflorin Regulates Microglia-Astrocyte Crosstalk, Inhibits Inflammatory Response, and Alleviates Neuropathic Pain through HSP90AA1/HMGB1 Signaling Pathway. Int. J. Biochem. Cell Biol..

[155] De Leon-Oliva D., Barrena-Blázquez S., Jiménez-Álvarez L., Fraile-Martinez O., García-Montero C., López-González L., Torres-Carranza D., García-Puente L.M., Carranza S.T., Álvarez-Mon M.Á. (2023). The RANK–RANKL–OPG System: A Multifaceted Regulator of Homeostasis, Immunity, and Cancer. Medicina.

[156] Alsamraae M., Cook L.M. (2021). Emerging Roles for Myeloid Immune Cells in Bone Metastasis. Cancer Metastasis Rev..

[157] Xia H., Zhang Z., Liu D., Yu D., Jia X. (2014). Establishment of Modern Multi-Component Sustained-Release Preparations of Oral Traditional Chinese Medicines. China J. Chin. Mater. Medica.

[158] Tibbitt M.W., Dahlman J.E., Langer R. (2016). Emerging Frontiers in Drug Delivery. J. Am. Chem. Soc..

[159] Sun G., Zhu Y., Liu Y., Wang J., Ping Q. (1996). Preparation and pharmacokinetics of tramadol hydrochloride extended-release tablets. J. China Pharm. Univ..

[160] Shinkai M., Katsumata N., Kawai S., Kuyama S., Sasaki O., Yanagita Y., Yoshida M., Uneda S., Tsuji Y., Harada H. (2024). Phase III Study of Bilayer Sustained-Release Tramadol Tablets in Patients with Cancer Pain: A Double-Blind Parallel-Group, Non-Inferiority Study with Immediate-Release Tramadol Capsules as an Active Comparator. Support. Care Cancer.

[161] Gourlay G.K. (1998). Sustained Relief of Chronic Pain. Pharmacokinetics of Sustained Release Morphine. Clin. Pharmacokinet..

[162] Xian Y., Xu Z., Jiang M., Pu D., Qi Y., Deng M., Wang F., Peng Y., Xiao Q., Yu C. (2020). Manufacture of Fine and Analgesic Drugs Using Slow and Controlled Release Technology and Compounded Double-Layer Preparation Technology.

[163] Schneider J.P., Matthews M., Jamison R.N. (2010). Abuse-Deterrent and Tamper-Resistant Opioid Formulations: What Is Their Role in Addressing Prescription Opioid Abuse?. CNS Drugs.

[164] Warfield C.A. (1998). Controlled-Release Morphine Tablets in Patients with Chronic Cancer Pain: A Narrative Review of Controlled Clinical Trials. Cancer.

[165] Lapin J., Houde R.W., Kaiko R.F., Coyle N., Rogers A., Foley K.M. (1989). Cancer Pain Management with a Controlled-Release Oral Morphine Preparation. J. Pain Symptom Manag..

[166] Jug M., Hafner A., Lovrić J., Kregar M.L., Pepić I., Vanić Ž., Cetina-Čižmek B., Filipović-Grčić J. (2018). An Overview of in Vitro Dissolution/Release Methods for Novel Mucosal Drug Delivery Systems. J. Pharm. Biomed. Anal..

[167] Lü F., Sui L., Liu Z. (2022). Research progress of delivery strategies related mucus barrier in mucosal drug delivery. Acta Pharm. Sin..

[168] Golshani S., Vatanara A., Amin M. (2022). Recent Advances in Oral Mucoadhesive Drug Delivery. J. Pharm. Pharm. Sci..

[169] Guitart-Vela J., Magrone Á., González G., Folch J. (2024). Effectiveness and Safety of Sublingual Fentanyl in the Treatment of Breakthrough Cancer Pain in Older Patients with Cancer: Results from a Retrospective Observational Study. J. Pain Palliat. Care Pharmacother..

[170] Garnock-Jones K.P. (2016). Fentanyl Buccal Soluble Film: A Review in Breakthrough Cancer Pain. Clin. Drug Investig..

[171] Chiang Y., Lien C.-T., Su W., Yen T.-G., Chen Y., Lai Y.-L., Lim K.-H., Dai K.-Y., Chung H.-P., Hung C.-Y. (2024). Effectiveness of Fentanyl Buccal Soluble Film in Cancer Patients with Inadequate Breakthrough Pain Control. BMC Palliat. Care.

[172] Feigal E.G., Parikh N., Kottayil G., Fisher D. (2008). Pharmacokinetic Profile of Fentanyl Sublingual (SL) Spray. J. Clin. Oncol..

[173] Rauck R., Oh D.A., Parikh N., Koch C., Singla N., Yu J., Nalamachu S., Vetticaden S. (2017). Pharmacokinetics and Safety of Fentanyl Sublingual Spray and Fentanyl Citrate Intravenous: A Single Ascending Dose Study in Opioid-Naïve Healthy Volunteers. Curr. Med. Res. Opin..

[174] Lin R., Song B., Li N., Rong B., Bai J., Liu Y., Wang W., Liu A., Luo S., Liu B. (2024). Efficacy and Safety of Fentanyl Inhalant for the Treatment of Breakthrough Cancer Pain: A Multicenter, Randomized, Double-Blind, Placebo-Controlled Trial. BMC Palliat. Care.

[175] Gao M., Shen X., Mao S. (2020). Factors Influencing Drug Deposition in Thenasal Cavity upon Delivery via Nasal Sprays. J. Pharm. Investig..

[176] Grassin-Delyle S., Buenestado A., Naline E., Faisy C., Blouquit-Laye S., Couderc L.-J., Le Guen M., Fischler M., Devillier P. (2012). Intranasal Drug Delivery: An Efficient and Non-Invasive Route for Systemic Administration: Focus on Opioids. Pharmacol. Ther..

[177] Dietrich E., Gums J.G. (2012). Intranasal Fentanyl Spray: A Novel Dosage Form for the Treatment of Breakthrough Cancer Pain. Ann. Pharmacother..

[178] Nakhaee S., Saeedi F., Mehrpour O. (2023). Clinical and Pharmacokinetics Overview of Intranasal Administration of Fentanyl. Heliyon.

[179] Phatale V., Vaiphei K.K., Jha S., Patil D., Agrawal M., Alexander A. (2022). Overcoming Skin Barriers through Advanced Transdermal Drug Delivery Approaches. J. Control. Release.

[180] Al Hanbali O.A., Khan H.M.S., Sarfraz M., Arafat M., Ijaz S., Hameed A. (2019). Transdermal Patches: Design and Current Approaches to Painless Drug Delivery. Acta Pharmaceut..

[181] Ulagenthiran A., Howard P., Curtin J. (2024). Transdermal Clonidine for Agitation and Pain. BMJ Support. Palliat. Care.

[182] De Marco I. (2023). Transdermal Patches Containing Opioids in the Treatment of Patients with Chronic Pain. Processes.

[183] Liu Y., Li Q., Yu Y., Wang J.J., Shi H.P. (2020). A Single-Arm, Prospective, Multicenter Study on the Efficacy and Safety of Low-Dose Transdermal Fentanyl Patch in Opioid-Naive Patients with Moderate to Severe Cancer Pain. J. Clin. Oncol..

[184] Yamaguchi S., Uchida E., Terahara T., Okawa K., Hashimoto F., Tanaka Y. (2020). Efficacy and Safety of Fentanyl Citrate Patch, Including a Low-Dose 0.5 Mg Formulation, in Opioid-Naïve Patients with Cancer Pain. Clin. Drug Investig..

[185] Galer B.S., Jensen M.P., Ma T., Davies P.S., Rowbotham M.C. (2002). The Lidocaine Patch 5% Effectively Treats All Neuropathic Pain Qualities: Results of a Randomized, Double-Blind, Vehicle-Controlled, 3-Week Efficacy Study With Use of the Neuropathic Pain Scale. Clin. J. Pain.

[186] Baron R., Mayoral V., Leijon G., Binder A., Steigerwald I., Serpell M. (2009). 5% Lidocaine Medicated Plaster versus Pregabalin in Post-Herpetic Neuralgia and Diabetic Polyneuropathy: An Open-Label, Non-Inferiority Two-Stage RCT Study. Curr. Med. Res. Opin..

[187] Tsai J.-H., Liu I.-T., Su P.-F., Huang Y.-T., Chiu G.-L., Chen Y.-Y., Lai W.-S., Lin P.-C. (2023). Lidocaine Transdermal Patches Reduced Pain Intensity in Neuropathic Cancer Patients Already Receiving Opioid Treatment. BMC Palliat. Care.

[188] Yu Z., Chen W., Ding Y., Jiang L., Gu P., Zhao H. (2010). Diclofenac Sodium Patch.

[189] Chen M., Xiu M., Yang L., Jin H., Deng L., Li Y., Chen P. (2024). Intrathecal Drug-Infusion System for Treating Pain in Advanced Cancer: New Research in Patient Management. Int. J. Clin. Pharmacol. Ther..

[190] Sindt J.E., Odell D.W., Dalley A.P., Brogan S.E. (2020). Initiation of Intrathecal Drug Delivery Dramatically Reduces Systemic Opioid Use in Patients With Advanced Cancer. Neuromodul. Technol. Neural Interface.

[191] Delhaas E.M., Harhangi B.S., Frankema S.P.G., Huygen F.J.P.M., Van Der Lugt A. (2017). Plain Radiography in Patients Treated with Intrathecal Drug Delivery Using an Implantable Pump Device. Insights Imaging.

[192] Long D., Li X., Zhang Y., Luo J., Liu B., Hong B., Yang F., Zou C., Ge F., Zhang A. (2024). Intrathecal Drug Delivery System in Prepontine Cistern for Patients with Intractable Craniofacial Cancer Pain: A Multicenter Retrospective Study. Anesth. Analg..

[193] Li Q., Long Y., He Y., Long H., Xiao Z., Li Y., Yang W., Jiang L., Gao W., Zou C. (2024). Intrathecal Morphine Delivery at Prepontine Cistern to Control Refractory Cancer-Related Pain: A Case Report of Extensive Metastatic and Refractory Cancer Pain. BMC Anesthesiol..

[194] Zhou H., Huang D., Zou D., Hu J., Li X., Wang Y. (2022). Prepontine Cisternal Routine for Intrathecal Targeted Drug Delivery in Craniofacial Cancer Pain Treatment: Technical Note. Drug Deliv..

[195] Huang G., Liu G., Zhou Z., Yang J., Su C. (2020). Successful Treatment of Refractory Cancer Pain and Depression with Continuous Intrathecal Administration of Dexmedetomidine and Morphine: A Case Report. Pain Ther..

[196] Nguyen H.X., Banga A.K. (2024). Advanced Transdermal Drug Delivery System: A Comprehensive Review of Microneedle Technologies, Novel Designs, Diverse Applications, and Critical Challenges. Int. J. Pharm..

[197] Shen C., Meng G., Wang Q., Shi Z., Wang D. (2020). Preparation and quality evaluation of lidocaine cataplasms. Chin. J. New Drugs.

[198] Taguchi T., Yamaguchi S., Terahara T., Okawa K., Inakura H. (2023). Systemically Acting Diclofenac Sodium Patch for Control of Low Back Pain: A Randomized, Double-Blind, Placebo-Controlled Study in Japan. Pain Ther..

[199] Yamaguchi S., Terahara T., Okawa K., Inakura H. (2021). A Multicenter, Randomized, Double-Blind, Placebo-Controlled, Comparative Study to Evaluate the Efficacy and Safety of Newly Developed Diclofenac Patches in Patients with Cancer Pain. Pain.

[200] Adhikary S., Al Hoque A., Ray M., Pal P., Chaudhuri M.G., Dey R. (2024). Tailored Transdermal Drug Delivery System for Pain Management: Development and Evaluation of Clonidine Hydrochloride/Sodium Montmorillonite Composite Patch. BioNanoScience.

[201] Le Z., Yu J., Quek Y.J., Bai B., Li X., Shou Y., Myint B., Xu C., Tay A. (2023). Design Principles of Microneedles for Drug Delivery and Sampling Applications. Mater. Today.

[202] Li M., Vora L.K., Peng K., Sabri A.H.B., Qin N., Abbate M., Paredes A.J., McCarthy H.O., Donnelly R.F. (2024). Novel Nano-in-Micro Fabrication Technique of Diclofenac Nanoparticles Loaded Microneedle Patches for Localised and Systemic Drug Delivery. Biomater. Adv..

[203] Zhao Z.Q., Zhang B.L., Chu H.Q., Liang L., Chen B.Z., Zheng H., Guo X.D. (2022). A High-Dosage Microneedle for Programmable Lidocaine Delivery and Enhanced Local Long-Lasting Analgesia. Biomater. Adv..

[204] Brighton P.J., Szekeres P.G., Willars G.B. (2004). Neuromedin U and Its Receptors: Structure, Function, and Physiological Roles. Pharmacol. Rev..

[205] Peng S., Lu Y., Li P., Liu P., Shi X., Liu C., Zhang Y., Liu S., Wang J. (2019). The Short Interference RNA (siRNA) Targeting NMUR2 Relieves Nociception in a Bone Cancer Pain Model of Rat through PKC-ERK and PI3K-AKT Pathways. Biochem. Biophys. Res. Commun..

[206] de Brito T.V., Júnior G.J.D., Júnior J.S.d.C., Silva R.O., Monteiro C.E.d.S., Franco A.X., Vasconcelos D.F.P., de Oliveira J.S., Costa D.V.d.S., Carneiro T.B. (2020). Gabapentin Attenuates Intestinal Inflammation: Role of PPAR-Gamma Receptor. Eur. J. Pharmacol..

[207] Fu J., Zhao B., Ni C., Ni H., Xu L., He Q., Xu M., Xu C., Luo G., Zhu J. (2021). Rosiglitazone Alleviates Mechanical Allodynia of Rats with Bone Cancer Pain through the Activation of PPAR-γ to Inhibit the NF-κB/NLRP3 Inflammatory Axis in Spinal Cord Neurons. PPAR Res..

[208] He X.-T., Hu X.-F., Zhu C., Zhou K.-X., Zhao W.-J., Zhang C., Han X., Wu C.-L., Wei Y.-Y., Wang W. (2020). Suppression of Histone Deacetylases by SAHA Relieves Bone Cancer Pain in Rats via Inhibiting Activation of Glial Cells in Spinal Dorsal Horn and Dorsal Root Ganglia. J. Neuroinflamm..

[209] Ding Y. (2025). Histone Deacetylases: The Critical Enzymes for Microglial Activation Involved in Neuropathic Pain. Front. Pharmacol..

[210] Yang C., Kang F., Wang S., Han M., Zhang Z., Li J. (2019). SIRT1 Activation Attenuates Bone Cancer Pain by Inhibiting mGluR1/5. Cell Mol. Neurobiol..

[211] Yang C., Huang X., Wang S., Han M., Kang F., Zhang Z., Li J. (2020). Intrathecal Administration of SRT1720 Relieves Bone Cancer Pain by Inhibiting the CREB/CRTC1 Signalling Pathway. Neurosci. Lett..

[212] Dansereau M.-A., Midavaine É., Bégin-Lavallée V., Belkouch M., Beaudet N., Longpré J.-M., Mélik-Parsadaniantz S., Sarret P. (2021). Mechanistic Insights into the Role of the Chemokine CCL2/CCR2 Axis in Dorsal Root Ganglia to Peripheral Inflammation and Pain Hypersensitivity. J. Neuroinflamm..

[213] Midavaine É., Brouillette R.L., Théberge E., Mona C.E., Kashem S.W., Côté J., Zeugin V., Besserer-Offroy É., Longpré J.-M., Marsault É. (2024). Discovery of a CCR2-Targeting Pepducin Therapy for Chronic Pain. Pharmacol. Res..

[214] Chen Z., Xia Y., Liu B., Fang J., Hu Q. (2025). The CXCL12/CXCR4 Axis: An Emerging Therapeutic Target for Chronic Pain. J. Pain Res..

[215] Xu H., Peng C., Chen X.-T., Yao Y.-Y., Chen L.-P., Yin Q., Shen W. (2020). Chemokine Receptor CXCR4 Activates the RhoA/ROCK2 Pathway in Spinal Neurons That Induces Bone Cancer Pain. Mol. Pain..

[216] Lutz B.M., Bekker A., Tao Y.-X. (2014). Noncoding RNAs: New Players in Chronic Pain. Anesthesiology.

[217] Xu S., He L., Chen Y., Lin T., Tang L., Wu Y., He Y., Sun X. (2025). Clinical Implications of miR-195 in Cancer: Mechanisms, Potential Applications, and Therapeutic Strategies. J. Cancer Res. Clin. Oncol..

[218] Shi G., Shi J., Liu K., Liu N., Wang Y., Fu Z., Ding J., Jia L., Yuan W. (2013). Increased miR-195 Aggravates Neuropathic Pain by Inhibiting Autophagy Following Peripheral Nerve Injury. Glia.

[219] D’Oronzo S., Coleman R., Brown J., Silvestris F. (2019). Metastatic Bone Disease: Pathogenesis and Therapeutic Options: Up-Date on Bone Metastasis Management. J. Bone Oncol..

[220] Rossi F., Tortora C., Punzo F., Bellini G., Argenziano M., Di Paola A., Torella M., Perrotta S. (2019). The Endocannabinoid/Endovanilloid System in Bone: From Osteoporosis to Osteosarcoma. Int. J. Mol. Sci..

[221] Zhu C., Wang K., Chen Z., Han Y., Chen H., Li Q., Liu Z., Qian L., Tang J., Shen H. (2020). Antinociceptive Effect of Intrathecal Injection of miR-9-5p Modified Mouse Bone Marrow Mesenchymal Stem Cells on a Mouse Model of Bone Cancer Pain. J. Neuroinflamm..

[222] Kuang J., Xu M., Xu C., Wang Y., Ni C., Wei S., Liu Z., Kong M., Zhou Q., Yao M. (2023). miR-199a-3p Mediates Bone Cancer Pain through Upregulation of Dnmt3a Expression in Spinal Dorsal Horn Neurons. Biochem. Biophys. Res. Commun..

[223] Ni H., Xu M., Xie K., Fei Y., Deng H., He Q., Wang T., Liu S., Zhu J., Xu L. (2020). Liquiritin Alleviates Pain Through Inhibiting CXCL1/CXCR2 Signaling Pathway in Bone Cancer Pain Rat. Front. Pharmacol..

[224] Andreu V., Arruebo M. (2018). Current Progress and Challenges of Nanoparticle-Based Therapeutics in Pain Management. J. Control. Release.

[225] Dash S.R., Kundu C.N. (2023). Cancer-Induced Pain Management by Nanotechnology-Based Approach. Curr. Pharm. Biotechnol..

[226] Satapathy T., Sahu D., Sahu H., Pandey R.K., Shukla S.S., Gidwani B. (2024). Trends on Nanomedicines as Novel Therapeutics Approach in Targeting Nociceptors for Relieving Pain. Curr. Drug Targets.

[227] Zhang J., Zhu S., Tan Q., Cheng D., Dai Q., Yang Z., Zhang L., Li F., Zuo Y., Dai W. (2020). Combination Therapy with Ropivacaine-Loaded Liposomes and Nutrient Deprivation for Simultaneous Cancer Therapy and Cancer Pain Relief. Theranostics.

[228] Bahrami M.A., Farhadian N., Karimi M., Forouzan A., Masoumi K. (2020). Improvement of Pain Relief of Fentanyl Citrate Drug Encapsulated in Nanostructured Lipid Carrier: Drug Formulation, Parameter Optimization, in Vitro and in Vivo Studies. Drug Des. Dev. Ther..

[229] Zhou Y. (2024). Morphine Sulfate Nano-Controlled Release Microspheres Effectively Relieve Visceral Pain Caused by Tumor in Mice. Am. J. Transl. Res..

[230] Bhansali D., Tu N.H., Inoue K., Teng S., Li T., Tran H.D., Kim D.H., Dong J., Peach C.J., Sokrat B. (2025). PAR2 on Oral Cancer Cells and Nociceptors Contributes to Oral Cancer Pain That Can Be Relieved by Nanoparticle-Encapsulated AZ3451. Biomaterials.

[231] Lv X., Wang F., Liu X., Xu T., Zhou X. (2025). Magnetic Nanoparticles-Based Targeted Drug Delivery System in Tumor Pain Management. SLAS Technol..

[232] Tang Y., Zhang J., Yuan Y., Shen K., Luo Z., Jia L., Long X., Peng C., Xie T., Chen X. (2024). Synergistic Gas Therapy and Targeted Interventional Ablation With Size-Controllable Arsenic Sulfide (As_2_S_3_) Nanoparticles for Effective Elimination of Localized Cancer Pain. Small.

[233] Chu X., Zhuang H., Liu Y., Li J., Wang Y., Jiang Y., Zhang H., Zhao P., Chen Y., Jiang X. (2022). Blocking Cancer–Nerve Crosstalk for Treatment of Metastatic Bone Cancer Pain. Adv. Mater..

[234] Cao D.-C., Liang Y., Guo Y., Wu D.-Y., Wang N.-N., Li Y.-M., Sun H.-F., Wang Q., Zhang X., Chi Y.-L. (2025). Oxygen-Generating Biomimetic Nano-Herb System for Synergistic Therapy & Pain Relief in Triple-Negative Breast Cancer via HIF-1α/VEGF Pathway. Int. J. Nanomed..

[235] Zhang P., Yao S., Tang Y., Wan S., Chen X., Ma L. (2023). A Side-Effect-Free Interventional Therapy for Precisely Eliminating Unresectable Cancer Pain. ACS Nano.

[236] Zorzetto L., Brambilla P., Marcello E., Bloise N., De Gregori M., Cobianchi L., Peloso A., Allegri M., Visai L., Petrini P. (2016). From Micro- to Nanostructured Implantable Device for Local Anesthetic Delivery. Int. J. Nanomed..

[237] Hua S., Wu S.Y. (2013). The Use of Lipid-Based Nanocarriers for Targeted Pain Therapies. Front. Pharmacol..

[238] Wu T., Wu H., Wang Q., He X., Shi P., Yu B., Cong H., Shen Y. (2024). Current Status and Future Developments of Biopolymer Microspheres in the Field of Pharmaceutical Preparation. Adv. Colloid. Interface Sci..

[239] Zhu L., Zhou Z., Mao H., Yang L. (2017). Magnetic Nanoparticles for Precision Oncology: Theranostic Magnetic Iron Oxide Nanoparticles for Image-Guided and Targeted Cancer Therapy. Nanomedicine.

[240] Stueber D.D., Villanova J., Aponte I., Xiao Z., Colvin V.L. (2021). Magnetic Nanoparticles in Biology and Medicine: Past, Present, and Future Trends. Pharmaceutics.

[241] Xiao H., Raza F., Li K., Song J., Zafar H., Yang S., Su J., Qiu M. (2025). Cell Membrane Derived Biomimetic Nanomedicine for Precision Delivery of Traditional Chinese Medicine in Cancer Therapy. J. Control. Release.

[242] Li Y., Wang Y., Huang G., Gao J. (2018). Cooperativity Principles in Self-Assembled Nanomedicine. Chem. Rev..

[243] Villanueva M.T. (2017). Designing out Opioid Side Effects. Nat. Rev. Drug Discov..

[244] Jin Y.-H., Wang Y.-P., Xie Y.-L., Tian G.-H., Zhang X.-Y., Shi N.-N., Yang K.-H., Sun X., Chen Y.-L., Wu D.-R. (2023). Research on the Development Methodology for Clinical Practice Guidelines for Organic Integration of Traditional Chinese and Western Medicine. Mil. Med. Res..

[245] Bohren Y., Cachemaille M., Timbolschi I.D., Perruchoud C. (2025). Understanding the Physiopathology of Pain Pathways for a Practical Approach of Cancer Pain Management. Cardiovasc. Intervent Radiol..

[246] Kim J., Jesus O.D. (2023). Medication Routes of Administration. StatPearls.

[247] Sato H., Yamada K., Miyake M., Onoue S. (2023). Recent Advancements in the Development of Nanocarriers for Mucosal Drug Delivery Systems to Control Oral Absorption. Pharmaceutics.

[248] Le-Vinh B., Steinbring C., Wibel R., Friedl J.D., Bernkop-Schnürch A. (2021). Size Shifting of Solid Lipid Nanoparticle System Triggered by Alkaline Phosphatase for Site Specific Mucosal Drug Delivery. Eur. J. Pharm. Biopharm..

[249] Kang Y., Zhang S., Wang G., Yan Z., Wu G., Tang L., Wang W. (2024). Nanocarrier-Based Transdermal Drug Delivery Systems for Dermatological Therapy. Pharmaceutics.

[250] Jiang T., Xu G., Chen G., Zheng Y., He B., Gu Z. (2020). Progress in Transdermal Drug Delivery Systems for Cancer Therapy. Nano Res..

[251] Duarte R., Copley S., Nevitt S., Maden M., Al-Ali A.M., Dupoiron D., Eldabe S. (2023). Effectiveness and Safety of Intrathecal Drug Delivery Systems for the Management of Cancer Pain: A Systematic Review and Meta-Analysis. Neuromodul. Technol. Neural Interface.

[252] Health Quality Ontario (2024). Intrathecal Drug Delivery Systems for Cancer Pain: A Health Technology Assessment. Ont. Health Technol. Assess. Ser..

[253] Goel V., Kumar V., Blaes A., Gulati A. (2023). Intrathecal Drug Delivery Systems for Cancer Pain Control: Insights on Current Contemporary Practices in the US. Neuromodul. Technol. Neural Interface.

[254] Ciftci F., Özarslan A.C., Kantarci İ.C., Yelkenci A., Tavukcuoglu O., Ghorbanpour M. (2025). Advances in Drug Targeting, Drug Delivery, and Nanotechnology Applications: Therapeutic Significance in Cancer Treatment. Pharmaceutics.

